# Correspondence between white matter hyperintensities and regional grey matter volumes in Alzheimer's disease

**DOI:** 10.3389/fnagi.2024.1429098

**Published:** 2024-09-16

**Authors:** Fangyuan Yi, Jirui Wang, Meiqing Lin, Baizhu Li, Shiyu Han, Shan Wang, Yingbin Jin, Ning Hu, Yutong Chen, Xiuli Shang

**Affiliations:** ^1^Department of Neurology, The First Affiliated Hospital of China Medical University, Shenyang, China; ^2^Department of Neurology, Jin Qiu Hospital of Liaoning Province (Geriatric Hospital of Liaoning Province), Shenyang, China

**Keywords:** Alzheimer’s disease, white matter fiber tracts, white matter hyperintensity, grey matter atrophy, cognition

## Abstract

**Objective:**

White matter hyperintensities (WMH) are the most common neuroimaging manifestation of cerebral small vessel disease, and is frequently observed in Alzheimer’s disease (AD). This study aimed to investigate the relationship between WMH and cognition and to verify the mediation of grey matter atrophy in this relationship.

**Methods:**

The diffusion tensor imaging (DTI) technique analyses white matter fiber tract to assess white matter integrity. Voxel-based morphometry was applied to measure the grey matter volume (GMV). A linear regression model was applied to examine the associations between WMH and GMV, and mediation analyses was performed to determine the mediating role of regional GMV in the effect of WMH on cognitive function.

**Results:**

Compared to the HC group, AD group have 8 fiber tract fractional anisotropy (FA) decreased and 16 fiber tract mean diffusivity (MD) increased. Compared to AD without WMH, AD with high WMH had 9 fiber tracts FA decreased and 13 fiber tracts MD increased. High WMH volume was negatively correlated with GMV in the frontal–parietal region. Low WMH volume was also negatively correlated with GMV except for the three regions (right angular gyrus, right superior frontal gyrus and right middle/inferior parietal gyrus), where GMV was positively correlated. Mediation analysis showed that the association between WMH and executive function or episodic memory were mediated by GMV in the frontal–parietal region.

**Conclusion:**

Damage to white matter integrity was more severe in AD with WMH. Differential changes in DTI metrics may be caused by progressive myelin and axonal damage. There was a negative correlation between WMH and grey matter atrophy in frontal–parietal regions in a volume-dependent manner. This study indicates the correspondence between WMH volume and GMV in cognition, and GMV being a key modulator between WMH and cognition in AD. This result will contribute to understanding the progression of the disease process and applying targeted therapeutic intervention in the earlier stage to delay neurodegenerative changes in frontal–parietal regions to achieve better treatment outcomes and affordability.

## Highlights

- Damage to white matter integrity was more severe in Alzheimer's disease (AD) with white matter hyperintensities (WMH), and mean diffusivity may be an earlier marker than fractional anisotropy in white matter regions.
- WMH is associated with global grey matter volume (GMV), especially frontal-parietal cortical atrophy.
- There were differential effects of WMH on grey matter atrophy in frontal-parietal regions in a volume-dependent manner. Low WMH volume had an unstable relationship with GMV, positively in the following regions (right angular gyrus, right superior frontal gyrus, and right middle and inferior parietal gyrus), but negatively in other frontal and parietal regions.
- We demonstrate the relationship between the interaction of WMH volume with GMV and cognition, with GMV being a key modulator of WMH and cognition in AD.

## 1 Introduction

Alzheimer’s disease (AD) is a progressive neurodegenerative disease associated with the accumulation of neurofibrillary tangles and amyloid-β (Aβ) deposition in the brain parenchyma and blood vessel walls (Salminen et al., 2015), resulting in neuronal damage (Frisoni et al., 2010). AD has commonly been considered a purely neurodegenerative alteration, but there is increasing evidence that this is too restrictive to explain the complex underlying pathological processes (De La Torre, 2002). In addition, vascular factors also put an individual with normal cognition at increased risk of progression along the AD spectrum (Leritz et al., 2011) and there have been links shown between vascular factors and pathological features of AD on autopsy (Erten-Lyons et al., 2013).

White matter hyperintensities (WMH) have been regarded as an important symptom of cerebral small vessel disease (Buckley et al., 2020). WMH is frequently observed in AD and other diseases, such as in the multiple sclerosis patients (Zhou, 2017). Previously, the vascular mechanisms of white matter hyperintensities and the pathological changes in Aβ were considered two different and independent pathways to AD (Benedictus et al., 2013). However, in studies of cerebral small vessel disease, high WMH volume has been associated with an increased risk of progression to medial temporal lobe atrophy and AD-related cognitive decline (Rizvi et al., 2018). WMH severity is intimately associated with substantial Aβ and amyloid precursor protein deposition at the molecular level, and not with traditional degenerative vascular disease such as atherosclerosis or small arteriosclerosis (Chalmers et al., 2005). These pathological changes are characteristic of AD. It is also found that WMH was associated with atrophy of AD pathologically susceptible brain regions, as well as with neuronal functional impairment reflected (Brickman et al., 2012). These findings emphasise the role of WMH in the pathological process of AD and suggest that WMH may be an important indicator for early diagnosis and therapeutic monitoring of AD (Kuller et al., 2003).

Furthermore, higher WMH is associated with reduced network-based grey matter volume (GMV), reduced local efficiency of brain networks (Reijmer et al., 2013) and reduced functional connectivity involving default mode and executive control networks (Tuladhar et al., 2015). Meanwhile, a decreased GMV is an important hallmark of the pathological process of AD, and it is strongly associated with cognitive decline. In pathological states of AD, WMH maybe related to regional GMV reduction, particularly in frontal and parietal regions (Ha et al., 2012). This also hypothesizes a correspondence between WMH and grey matter atrophy in AD. These pathologic processes certainly co-occur but may operate in different brain region.

Diffusion tensor imaging (DTI) is currently the only non-invasive method that can effectively observe and track the white matter (WM) fiber tracts in the human brain *in vivo* (Zhao et al., 2019) to observe WM microstructure and assess WM integrity. These parameters include anisotropy fraction (FA), mean diffusivity (MD), axial diffusivity and radial diffusivity. These changes in these indices are associated with pathological processes such as myelin formation, cell death, axonal damage and demyelination (Garnier-Crussard et al., 2022). The correlation between WM structural connectivity measured with FA. Moreover, the FA measure at baseline predicted longitudinal functional coordination measured with functional magnetic resonance imaging (MRI), indicating a possible initial WM inflammatory factor to the subsequent neurodegenerative processes (Zhou, 2017). Voxel-based morphometry is widely used for quantitative measurements of GMV to measure small volume changes. The greater WMH volume is associated with decreased GMV in some cognition-related brain regions and that decreased GMV is correlated with lower levels of cognitive domains such as episodic memory (Wang et al., 2020; Brugulat-Serrat et al., 2020). These findings suggest that the mechanisms through which WMH results in cognitive disorders might be associated with attenuated conduction in the fasciculus due to the impairment of white matter integrity or brain atrophy due to decreased GMV.

Current research has only focused on the effects of WM fiber tracts or cortical atrophy single factors on cognition. We consider cognition as a broad, multidimensional concept. It is not dependent on single regions but on the connectivity between different regions and their networks (Reijmer et al., 2013). The underlying mechanisms mediating the association between WMH and GMV on cognitive function also need to be further investigated. In the current study, we first examined the relationships between cognitive function as measured by a range of cognitive domains, WMH volume, the integrity of white matter tracts, and grey matter volume in patients with AD. We then investigated whether the extent of white matter integrity or grey matter atrophy played roles in the influence of WMH on cognitive function. Therefore, we used mediation models to test the hypothesis that GMV mediates the potential associations between WMH and cognitive function.

## 2 Methods

### 2.1 Participants

This study group consisted of 102 right-handed participants: 74 AD and 28 healthy controls (HC) devoid of cognitive impairments and with Minimum Mental State Examination (MMSE) scores ≥28. Additionally, all AD participants were recruited from inpatients or Dementia & Memory Disorders Clinic, whereas the HC individuals were selected from the general population of the same geographical region as the AD individuals. Our research underwent ethical approval by the Medical Research Ethics Committee, and all participating individuals or their representatives provided written informed consent. Participants diagnosed with AD met both the National Institute of Neurological and Communication Disorders and Stroke/Alzheimer’s Disease and Related Disorders Association (NINCDS-ADRDA) probable AD criteria (McKhann et al., 2011) and Diagnostic and Statistical Manual of Mental Disorders (Fourth Edition, DSM-IV) criteria for dementia (American Psychiatric Association, 2000). AD participants were categorized into three groups: AD-nonWMH (AD with non WMH, Fazekas scale 0, *n* = 29), AD-miWMH (AD with mild WMH, Fazekas scale 1–2, *n* = 25), and AD-moWMH (AD with moderate WMH, Fazekas scale 3–4, *n* = 20), based on neuromaging features and Fazekas scale (Fazekas et al., 1987).

Exclusion criteria included: (Salminen et al., 2015) Fazekas scale score ≥ 5 was used to exclude severe WMH; (Frisoni et al., 2010) Hachinski Ischemic Scale score ≥ 4 was used to exclude vascular dementia (Hachinski et al., 1975); (De La Torre, 2002) individuals with secondary causes of white matter lesions, such as demyelinating (multiple sclerosis, neuromyelitis optica spectrum disorders, etc.), metabolic (hypothyroidism, vitamin B12 deficiency, etc.), immunological, toxic, infectious, radiological and other causes; (Leritz et al., 2011) individuals with abnormal brain MRI findings such as head trauma, hemorrhage, infarction (except lacunes), seizures and other space-occupying lesions; (Erten-Lyons et al., 2013) individuals with intracranial/ extracranial artery stenosis >50%; (Buckley et al., 2020) evidence of intracranial calcification in the deep grey matter structures, hydrocephalus, subdural effusion; (Zhou, 2017) history of psychiatric disorders such as severe anxiety or depression (Hamilton anxiety scale ≥7 or Hamilton depression scale ≥8) (Hamilton, 1959; Hamilton, 1960); (Benedictus et al., 2013) individuals with visual or auditory impairments that interfere neuropsychological testing; (Rizvi et al., 2018) individuals with medication, drug dependence or abuse that possible impair cognition; (Chalmers et al., 2005) individuals who were unable to cooperate MRI test.

### 2.2 Neuropsychological measurement

All participants were assessed on standardized scales performed by an experienced neuropsychologist who evaluated global cognition as well as specific cognitive domains. Global cognition was assessed using the MMSE (Folstein et al., 1975) and Montreal Cognitive Assessment (MoCA-BJ) (Nasreddine et al., 2005). Cognitive information collected examined domains of (1) episodic memory, assessed using Wechsler Memory Scale Visual Reproduction-delayed recall (Wechsler, 2009) and auditory verbal learning test-delayed recall (Zhao et al., 2012); (2) executive function and processing speed, assessed using Stroop color and word tests (SCWT) (Weintraub et al., 2012) and trail making test (TMT) (Dubois et al., 2000), whereby SCWT was divided into three parts A, B and C (Stroop A, B, and C), and TMT was divided into two parts A and B (TMT-A and TMT-B); (3) language, assessed using categorical verbal fluency (Zhou et al., 2019) and Boston naming test (Ivanova et al., 2013); (4) visuospatial function, assessed using the Alzheimer’s Disease Assessment Scale-Cognitive constructional praxis test (Skinner et al., 2012) and block design test (Weintraub et al., 2012); (5) attention, assessed using coding (Joy et al., 2004) and digit span forward tests (Weintraub et al., 2012). Performance on the individual tasks was transformed into z-scores based on normative scores (Lee et al., 2012). In addition, the SCWT and TMT results were time-based, and the values were reverse-transformed to maintain the consistency of trends.

### 2.3 MRI scanning

All participants were examined on a multi-model Philip 3.0 T-MRI (Philips Medical Systems) with the parallel baseline scan from genu of corpus callosum to splenium, covering the whole brain. The scanning sequences included axial spin-echo T1-weighted imaging, T2-weighted imaging, fluid-attenuated inversion recovery (FLAIR). The acquisition parameters were as follows: FOV = 230 × 181, 230 × 230, 230 × 182 mm2; matrix = 340 × 201, 384 × 384, 352 × 136; slice thickness 6.5 mm, slice gap 1.2 mm, repetition time [TR] = 9,000 ms, echo time [TE] = 125 ms, interval time [TI] = 2,500 ms, in-plane spatial resolution of 0.4688 pixels/mm × 0.4688 pixels/mm. The DTI was performed with a single-shot spin-echo echo planar imaging sequence in 25 non-collinear directions (*b* = 1,000 s/mm2) and one additional image without diffusion weighting (*b* = 0 s/mm2). The acquisition parameters were as follows: TR = 9.154 ms, TE = 55 ms, flip angle = 15°. FOV = 224 mm × 224 mm, matrix = 112 × 112, voxel size 2 × 2 × 2.5 mm3, slice number = 65, slice thickness = 2 mm, and slice gap = 0 mm. The DTI scanning procedure lasted 5 min and 48 s.

#### 2.3.1 WMH volume measure

The axial T2 FLAIR images which were checked with the phase images were processed for the quantification of WMH volume by BIANCA (Brain Intensity Abnormality Classification Algorithm) software (Griffanti et al., 2016). Then the segmented lesions were examined visually and corrected manually by two experienced neuroradiologists who were blinded to all other imaging and clinical data. The manual correction process included: (Salminen et al., 2015) WMH area not properly labeled as WMH or normal appearing white matter falsely labeled as WMH; (Frisoni et al., 2010) Correction of non-white matter area being labeled as WMH. Due to each individual’s intracranial volume (ICV) being different, we measured the corrected WMH (cWMH) for further analysis. The following formula: cWMH volume = (WMH volume x mean ICV)/ICV. Mean ICV is the average ICV of all patients (O’Sullivan et al., 2002).

#### 2.3.2 DTI imaging measure

All DTI data was preprocessed with the FMRIB’s Software Library (Smith et al., 2004) (FSL 6.0; https://fsl.fmrib.ox.ac.uk/fsl/fslwiki/). The topup tool was applied to estimate the distortion of each diffusion-weighted image and b0 image, and the eddy tool was used to apply eddy current and head motion corrections to the images. The dtifit command was also applied to calculate the voxel maps of the tensor matrix and DTI metrics, including fractional anisotropy and mean diffusivity. Then, the voxel maps were registered to the FMRIB58_FA standard-space image in the Montreal Neurological Institute (MNI) standard space using FMRIB’s non-linear image registration tool.

**Figure 1:**
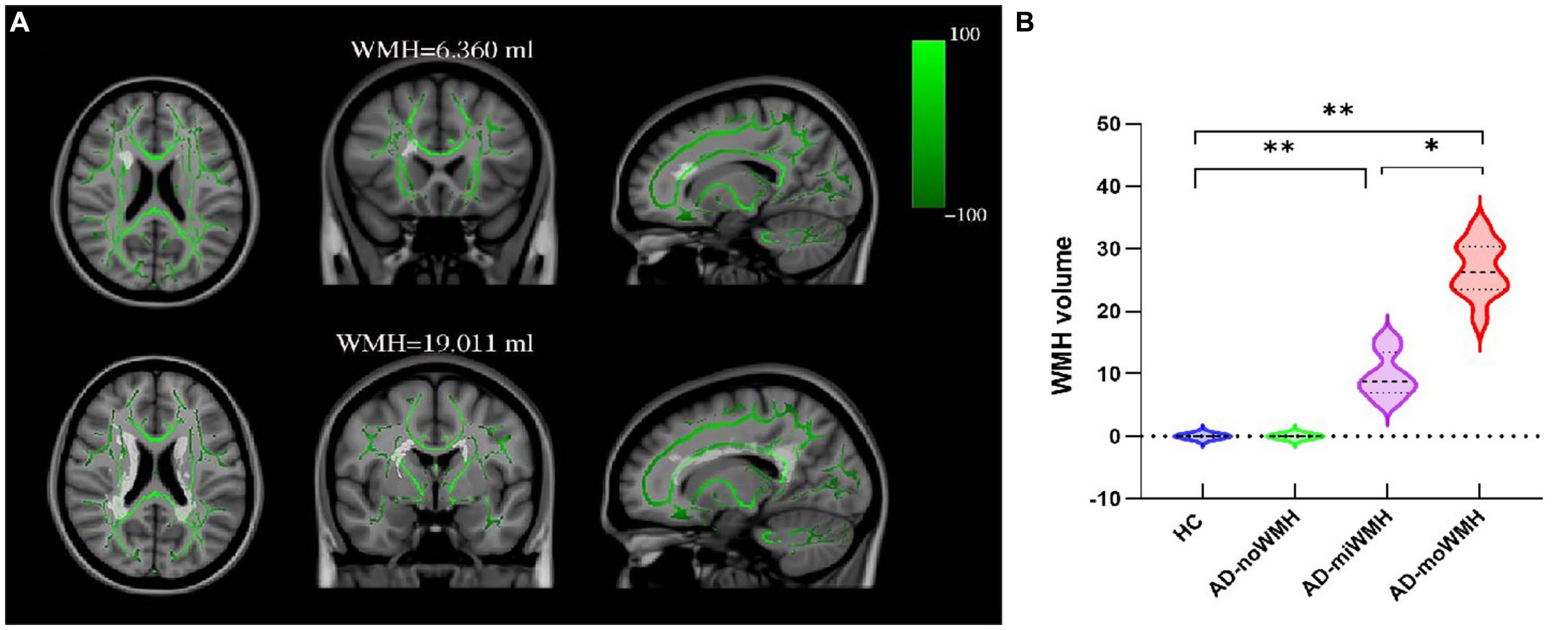
Show the position of WMH in relation to fiber tracts and the comparison of WMH volume in each group. **(A)** Shown was the relationship between WMH and white matter fiber tracts. WMH on FLAIR sequence WMH has superimposed on the TBSS-generated white matter skeleton (green) and the MNI152 1 mm standard space brain (horizontal, coronal and sagittal positions). **(B)** Shown was the alterations of global WMH volume for four groups. (* *p* < 0.05 or ** *p* < 0.001 was considered to indicate a significant difference). WMH, white matter hyperintensities; TBSS, tract-based spatial statistics; MNI, Montreal Neurological Institute.

Voxelwise statistical analysis of FA and MD datasets was carried out using tract-based spatial statistics (Smith et al., 2006) and created a mean FA image and a mean FA skeleton. Then, each participant’s aligned FA data was projected onto the mean FA skeleton as the target, and previously computed warps were successively applied to MD images, thereby transforming all images into MNI space for further analysis as above. Based on the WM atlas (i.e., JHU ICBM-DTI-81 WM labels and JHU White-Matter Tractography Atlas) (Mori et al., 2006), the DTI analysis were applied to assess WM fiber tracts parameters including: anterior thalamic radiation (ATR), corticospinal tract (CST), cingulum of the cingulate cortex (CC), cingulum of the hippocampus (CH), forceps major (FMa), forceps minor (FMi), inferior fronto-occipital fasciculus (IFOF), inferior longitudinal fasciculus (ILF), superior longitudinal fasciculus (SLF), uncinate fasciculus (UF) and superior longitudinal fasciculus-temporal part (tSLF). Shown was the relationship between WMH and fiber tracts, see Figure 1A.

#### 2.3.3 Grey matter volume

Grey matter volume was measured using Voxel-based analysis (Douaud et al., 2007). First, the brain extraction tool and FMRIB’s automated segmentation tool acquired grey matter partial volume was estimated from images of the whole head. Next, all segmented grey matter images were registered to the MNI152 standard space and averaged, and the study-specific grey matter template was created. Then, registration to the study-specific templates were run for all participants’ grey matter images, and modulated by the warp field expansion. Subsequently, the images were combined into 4D images and statistics. Running the “randomise” command and displaying cluster-based thresholding results.

### 2.4 Statistical analysis

All statistical analyses were using SPSS26.0 software (SPSS, version 26.0; IBM, Chicago, IL, USA). Descriptive statistics were generated to illustrate the sample characteristics. Demographic and cognitive scale assessment data were compared using t-test, chi-square test, and one-way analysis of variance (ANOVA) to test for differences between groups. DTI indicators were compared for each WM fiber tracts and corrected for familywise error correction (FWE). We used t-test and non-parametric test to compare the differences of GMV in different brain regions between all groups. Analyses were corrected for voxel-wise multiple comparisons using threshold free cluster enhancement (TFCE) and FWE. *p* < 0.05 was considered statistically significant.

A mediation analysis was conducted to test whether regional GMV mediated the effect of WMH on cognition using SPSS PROCESS. Specifically, each model used log-transformed WMH volume as a predictor, regional GMV as a mediator, and cognitive test score as an outcome. All analyses were corrected for age and gender. The standard error parameters of the mediation models were bootstrapped. The effect size, bootstrap standard error, bootstrap lower limit of confidence interval, bootstrap upper limit of confidence interval, and the proportion of relative effects of the total effect, direct effect, and mediating effect were reported.

## 3 Results

### 3.1 Demographic and clinical characteristics

Demographic and clinical characteristics for the groups are provided in Table 1. There were no significant differences for age, gender, and years of education (*p* > 0.05). The AD subgroups showed poor performances on MMSE, MoCA-BJ and five domain-specific cognition scores (*p* < 0.05), as detailed in Table 1. The WMH volume in the AD-moWMH group was 26.67 ± 4.25 mL, and WMH volume in the AD-miWMH group was 9.81 ± 3.42 mL. The alterations of global WMH volume for four groups were shown in Figure 1B.

**Table 1:** Demographic and neuropsychological data.

	HC (*n* = 28)	AD-nonWMH (*n* = 29)	AD-miWMH (*n* = 25)	AD-moWMH (*n* = 20)	F / χ^2^	*p*-value
Age (Y)	76.68 ± 8.92	74.06 ± 7.92	78.02 ± 6.81	79.78 ± 9.93	−2.311	0.633
Male (M / F)	16/12	14/15	16/9	13/7	2.041	0.721
Years of education (Y)	11.71 ± 2.03	12.01 ± 1.79	11.32 ± 1.96	11.46 ± 2.03	1.306	0.571
Vascular risk factors						
Hypertension (*n*, %)	11 (39.2)	9 (33.3)	11 (47.8)	8 (42.1)	0.827	0.454
Coronary heart disease (*n*, %)	7 (25.0)	5 (18.5)	5 (21.7)	7 (36.8)	0.722	0.603
Diabetes (*n*, %)	5 (17.9)	4 (14.8)	6 (26.1)	6 (21.1)	−1.375	0.366
Hyperlipidemia (*n*, %)	4 (14.3)	6 (22.2)	5 (21.7)	5 (26.3)	2.031	0.447
Smoke (*n*, %)	9 (32.1)	7 (25.9)	7 (30.4)	5 (26.3)	−0.994	0.652
HAMA (x ± s)	2.33 ± 0.19	1.95 ± 0.77	2.63 ± 0.72	2.55 ± 0.44	0.892	0.577
HAMD (x ± s)	2.07 ± 0.27	2.02 ± 0.44	2.37 ± 0.38	2.77 ± 0.72	0.837	0.619
General cognition						
MMSE	29.16 ± 0.31	22.53 ± 0.44	21.49 ± 0.97	18.32 ± 1.88	−1.997	0.011 ^A,B,C,E^
MoCA-BJ	26.77 ± 0.39	21.77 ± 0.52	20.95 ± 0.89	17.63 ± 0.92	−2.031	0.017 ^A,B,C,E^
Composition Z scores of each cognitive domain						
z score Episodic Memory	0.47 ± 0.12	−0.47 ± 0.37	−0.79 ± 0.55	−1.25 ± 0.49	33.712	0.021 ^A,B,C,D,E^
z score Executive Function	0.45 ± 0.21	−0.62 ± 0.33	−0.88 ± 0.27	−1.09 ± 0.34	35.292	0.033 ^A,B,C,D,E^
z score Language	0.29 ± 0.16	−0.82 ± 0.30	−0.77 ± 0.29	−0.89 ± 0.31	28.320	0.037 ^A,B,C^
z score Visuospatial Function	0.19 ± 0.11	−0.52 ± 0.38	−0.57 ± 0.33	−0.55 ± 0.45	16.517	0.029 ^A,B,C^
z score Attention	0.21 ± 0.14	−0.33 ± 0.15	−0.36 ± 0.20	−0.35 ± 0.22	19.776	0.042 ^A,B,C^

A HC vs AD-nonWMH, B HC vs AD-miWMH, C HC vs AD-moWMH, D AD-nonWMH vs AD-miWMH, E AD-nonWMH vs AD-moWMH. Superscript letters indicate whether group mean was significantly different healthy controls and AD-nonWMH (A), between healthy controls and AD-miWMH (B), between healthy controls and AD-moWMH (C), between AD-nonWMH and AD-miWMH (D), and between AD-nonWMH and AD-moWMH (E), based on post-hoc comparisons (p < 0.05) following one-way ANOVA. All values using mean ± standard deviation. HC, healthy control; AD, Alzheimer’s disease; WMH, white matter hyperintensities; MMSE, Mini-Mental State Examination. MoCA-BJ, the Beijing version of the Montreal Cognitive Assessment.

### 3.2 Group difference in WM fiber tract DTI indicator

**Figure 2:**
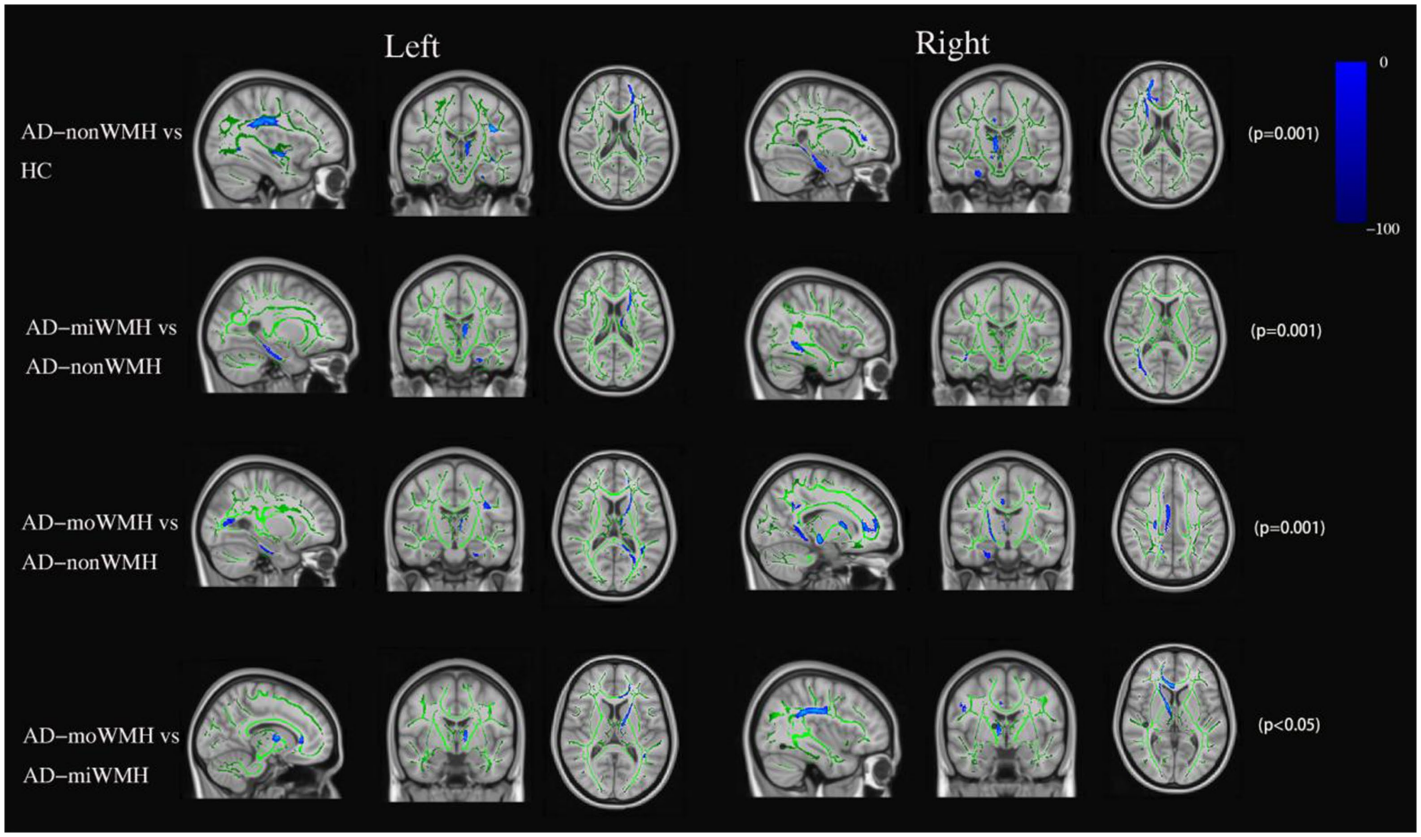
FA data are presented in radiological orientation and superimposed on the TBSS-generated white matter skeleton (green) and the MNI152 1 mm standard space brain. Shown are the results for analysis of the fractional anisotropy value declined (blue) in AD compared to HC and comparison among the AD subgroups using TBSS at pFWE <0.05. TBSS, tract-based spatial statistics; MNI, Montreal Neurological Institute; FWE, familywise error correction.

**Figure 3:**
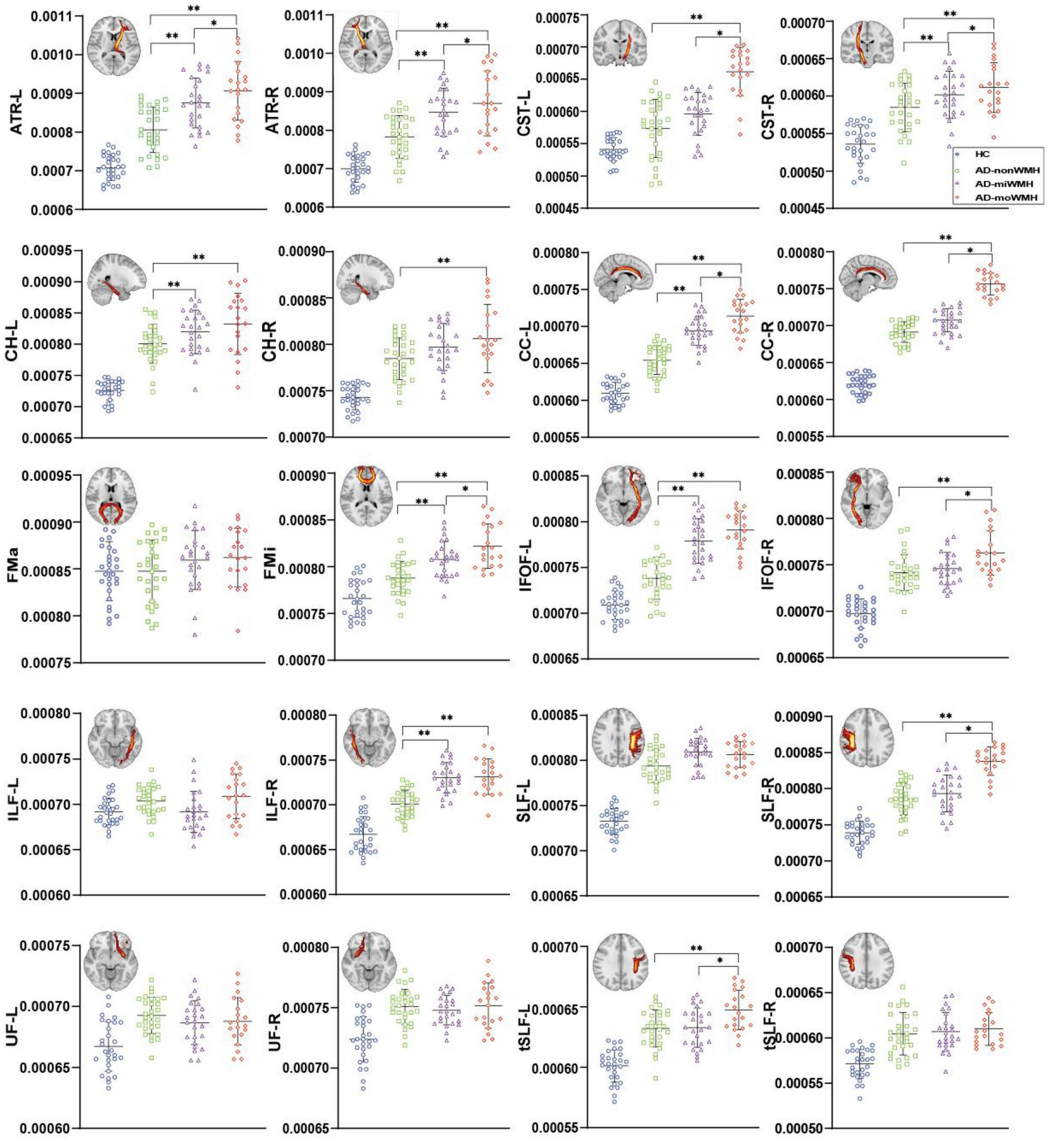
Graph was compared of MD values. Apart from the bilateral UF, FMa, and left ILF, the remaining WM fiber tracts exhibited significant differences between the AD group and the HC group (blue), p all <0.001. The results of comparing white matter fiber tracts in the AD groups are shown (*p < 0.05; **p < 0.001). ART: anterior thalamic radiation; CST: corticospinal tract; CH: cingulum of the hippocampus; CC: cingulum of the cingulate cortex; FMa: forceps major; FMi: forceps minor; IFOF: inferior fronto-occipital fasciculus; ILF: inferior longitudinal fasciculus; SLF: superior longitudinal fasciculus; UF: uncinate fasciculus; tSLF: superior longitudinal fasciculus-temporal part; L: left; R: right; MD, mean diffusivity; AD, Alzheimer’s disease; HC, healthy controls.

Analyses after FWE correction indicated that the AD group showed significantly decreased FA in 8 fiber tracts (bilateral ATR, bilateral CH, right CC, FMi, left IFOF, and left SLF) compared to the HC group. 4 fiber tracts (left ATR, left CH, and right ILF) in the AD-miWMH group had low FA compared to the AD-nonWMH group, whereas 9 fiber tracts (bilateral ATR, right CST, bilateral CH, right CC, FMa, FMi, and left tSLF) in the AD-moWMH group had low FA compared to the AD-nonWMH group (pFWE = 0.001), as shown in Figure 2. Similar to the FA, apart from the bilateral UF, FMa, and left ILF, the remaining WM fiber tracts exhibited significant differences between the AD group and the HC group. Compared to the AD-nonWMH group, the AD-miWMH group had significantly increased MD in 8 fiber tracts and the AD-moWMH group had significantly increased MD in 14 fiber tracts (FWE corrected, all *p* < 0.001), see Figure 3.

### 3.3 Comparison of regional grey matter volume

**Figure 4:**
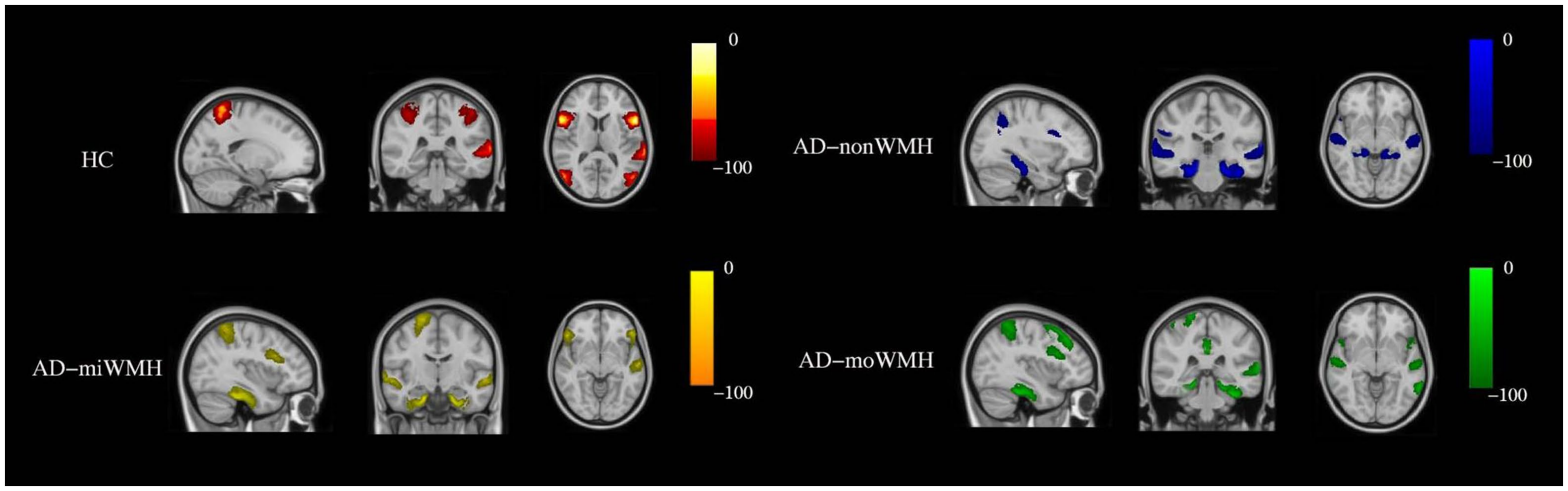
Show the sites of grey matter atrophy in each group. The results are presented in radiological orientation and superimposed on the MNI152 1 mm standard space brain (sagittal, coronal and horizontal positions). Grey matter atrophy was seen in the HC group (red-yellow) in the following regions - left inferior frontal gyrus, bilateral inferior inferior triangular frontal gyrus, left inferior temporal gyrus, bilateral superior parietal gyrus, and bilateral superior occipital gyrus. Grey matter atrophy was seen in the AD-nonWMH group (blue) in the following regions - right inferior frontal gyrus, left middle frontal gyrus, left angular gyrus, bilateral superior temporal gyrus, bilateral inferior temporal gyrus, and bilateral hippocampus. Grey matter atrophy was seen in the AD-miWMH group (yellow) in the following regions - right middle frontal gyrus, left superior frontal gyrus, right parietal gyrus, bilateral hippocampus and bilateral superior temporal gyrus. Grey matter atrophy was seen in the AD-moWMH group (green) in the following regions - right superior frontal gyrus, bilateral middle frontal gyrus, right superior parietal gyrus, left precuneus, bilateral middle temporal gyrus, left superior temporal gyrus, bilateral anterior cingulum and bilateral hippocampus. HC, healthy controls; AD-nonWMH, AD with non WMH; AD-miWMH, AD with mild WMH; AD-moWMH, AD with moderate WMH; MNI, Montreal Neurological Institute.

**Figure 5:**
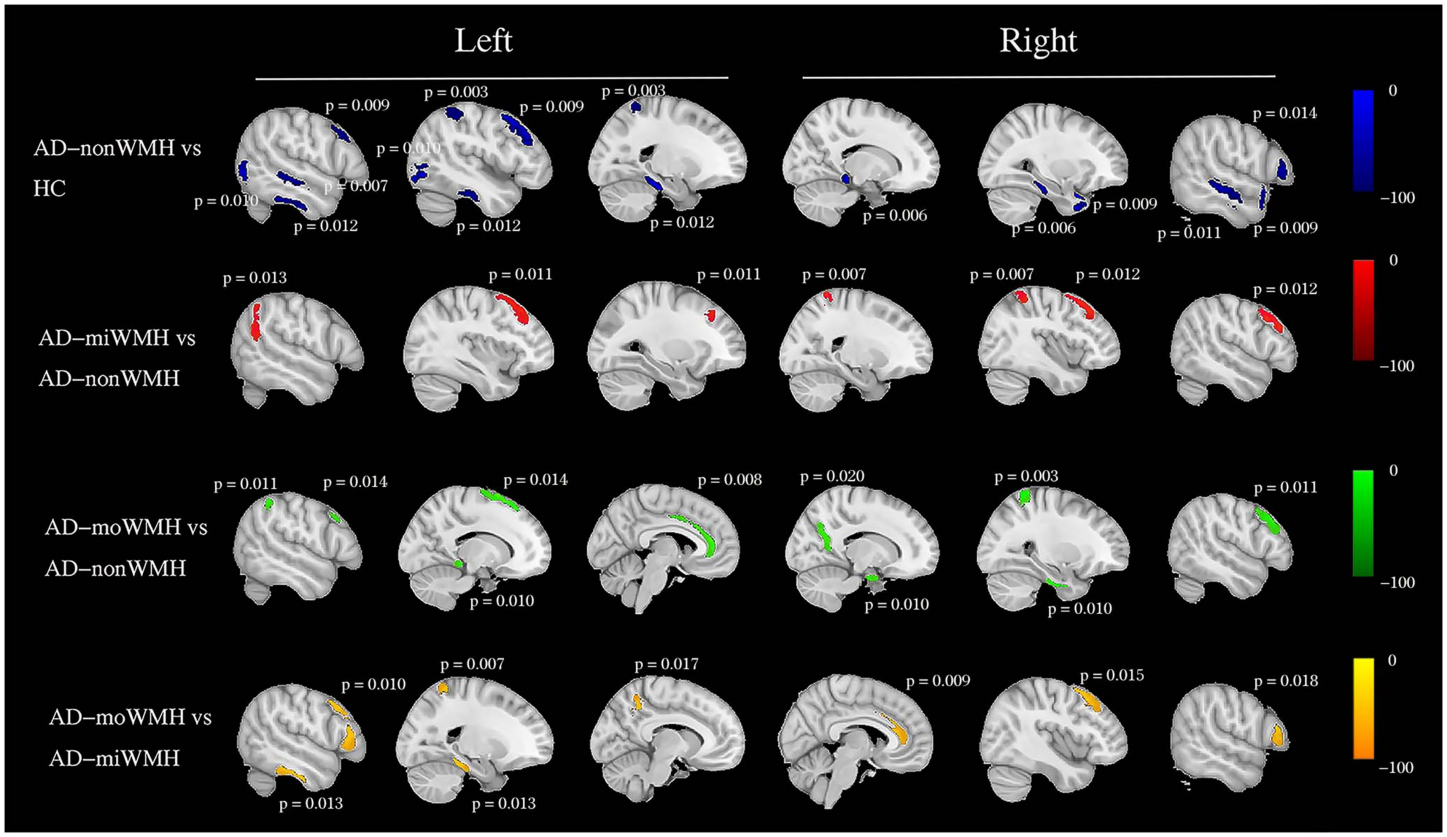
Group comparisons of grey matter volume using voxel-based morphometry. Schematic overview of the cerebral regions showing a statistically significant regional grey matter atrophy (blue) in AD with non WMH group compared to HC group after controlling for age, gender and cognition at pFWE <0.05. We showed a pairwise comparison between AD subgroups after controlling for age, gender, and cognition, with grey matter atrophy indicated in red (AD-miWMH vs. AD-nonWMH), green (AD-moWMH vs. AD-nonWMH) and yellow (AD-moWMH vs. AD-miWMH) respectively. The *p* values for the corresponding sites are marked in the figure (all pFWE <0.05). We showed the most widespread decreases in grey matter volume across the frontal and parietal regions in AD with low WMH volume while AD without WMH showed reduced grey matter volume mainly in temporal regions compared to HC. HC, healthy controls; AD, Alzheimer’s disease; WMH, white matter hyperintensities; FWE, familywise error correction; AD-nonWMH, AD with non WMH; AD-miWMH, AD with mild WMH; AD-moWMH, AD with moderate WMH.

We demonstrate regions of change in grey matter atrophy in each group, see Figure 4. We showed the most widespread GMV decreases across the frontal and parietal regions in AD with low WMH volume while AD without WMH showed reduced grey matter volume mainly in temporal regions compared to HC after further controlling for age, gender and cognition (*p* < 0.05), see Table 2 and Figure 5.

**Table 2:** Regions showing group differences in grey matter volume after controlling for age, gender and cognition.

Contrast	Regions	Peak t-statistics	Cluster size	MNI coordinates
HC and AD	Right superior temporal pole; right middle temporal gyrus	5.34	1,072	45, −16, −16
	Right superior temporal gyrus	3.96	507	62, −28, −4
	Right hippocampus; right parahippocampus	5.75	729	19, −30, −17
	right inferior triangular frontal gyrus	4.77	931	40, 10, 55
	Left hippocampus; left amygdala; left parahippocampus	3.92	1,120	−14, −35, −12
	Left middle temporal gyrus	4.10	320	−51, −36, −11
	Left inferior temporal gyrus	4.63	387	−22, −45, −5
	Left middle occipital gyrus	4.37	281	−30, −82, 8
	Left middle frontal gyrus	4.42	531	−42, 29, 36
	Left superior parietal gyrus	4.84	326	−17, −55, 65
AD subgroup				
AD-miWMH and AD-nonWMH	Right middle frontal gyrus	3.97	382	32, 15, 26
	Right superior parietal gyrus	5.03	429	41, −44, 58
	Left superior/medial frontal gyrus	4.72	693	−12, 44, 20
	Left angular gyrus	4.58	173	−48, −67, 35
AD-moWMH and AD-nonWMH	Right precuneus	5.27	510	28, −55, 60
	Right middle frontal gyrus	4.04	373	33, 17, 29
	Right superior parietal gyrus	4.19	689	19, −47, 72
	Right hippocampus; right amygdala	4.38	287	44, −15,-15
	Left superior/medial frontal gyrus	5.62	330	−10, 42, 24
	Left anterior cingulum	5.25	583	−13, 36, 22
	Left inferior parietal gyrus	4.63	428	−39, −42, 50
	Left hippocampus; left amygdala; left parahippocampus	4.92	309	−18, −10, −17
AD-moWMH and AD-miWMH	Right middle/inferior frontal gyrus	4.21	316	38, 11, 47
	Right superior temporal gyrus; right supramarginal gyrus	5.02	197	55, −17, 16
	Right anterior cingulum	4.29	323	10, 45, 23
	Left hippocampus; left inferior temporal gyrus; left parahippocampus	4.37	206	−38, −30, −7
	Left inferior triangular frontal gyrus; left middle frontal gyrus	3,85	337	−47, 13, 41
	Left superior parietal gyrus	4.76	270	−27, −57, 63
	Left inferior parietal gyrus; left precuneus	4.43	265	−39, −49, 47

Regional atrophy of grey matter volume in AD compared to HC and comparison among the AD subgroups after controlling for age, gender and cognition. WMH, white matter hyperintensity; HC, healthy controls; AD, Alzheimer’s disease; MNI, Montreal Neurological Institute.

### 3.4 Relationship between WMH volume and GMV

In a univariate analysis shown in Table 3, significant Spearman correlations were found between higher WMH volume and lower total GMV (*r* = −0.410, *p* = 0.031). WMH volume had a negative correlation with frontal and parietal GMV in AD patients (*r* = −0.422, −0.372; *p* = 0.022, 0.027; minimum cluster size = 100 voxels). WMH volume had no statistically different with temporal and occipital GMV in AD patients (*r* = −0.259, 0.520; *p* = 0.065, 0.113; minimum cluster size = 100 voxels).

**Table 3:** Spearman correlations between WMH and voxel-wise GMV.

Measure	Coefficient r	*p*
Total GMV	−0.410	0.031*
Frontal GMV	−0.422	0.022*
Parietal GMV	−0.370	0.027*
Temporal GMV	−0.259	0.065
Occipital GMV	0.520	0.113

*p < 0.05 was considered to indicate a significant difference. WMH, white matter hyperintensity volume; GMV, grey matter volume.

**Figure 6:**
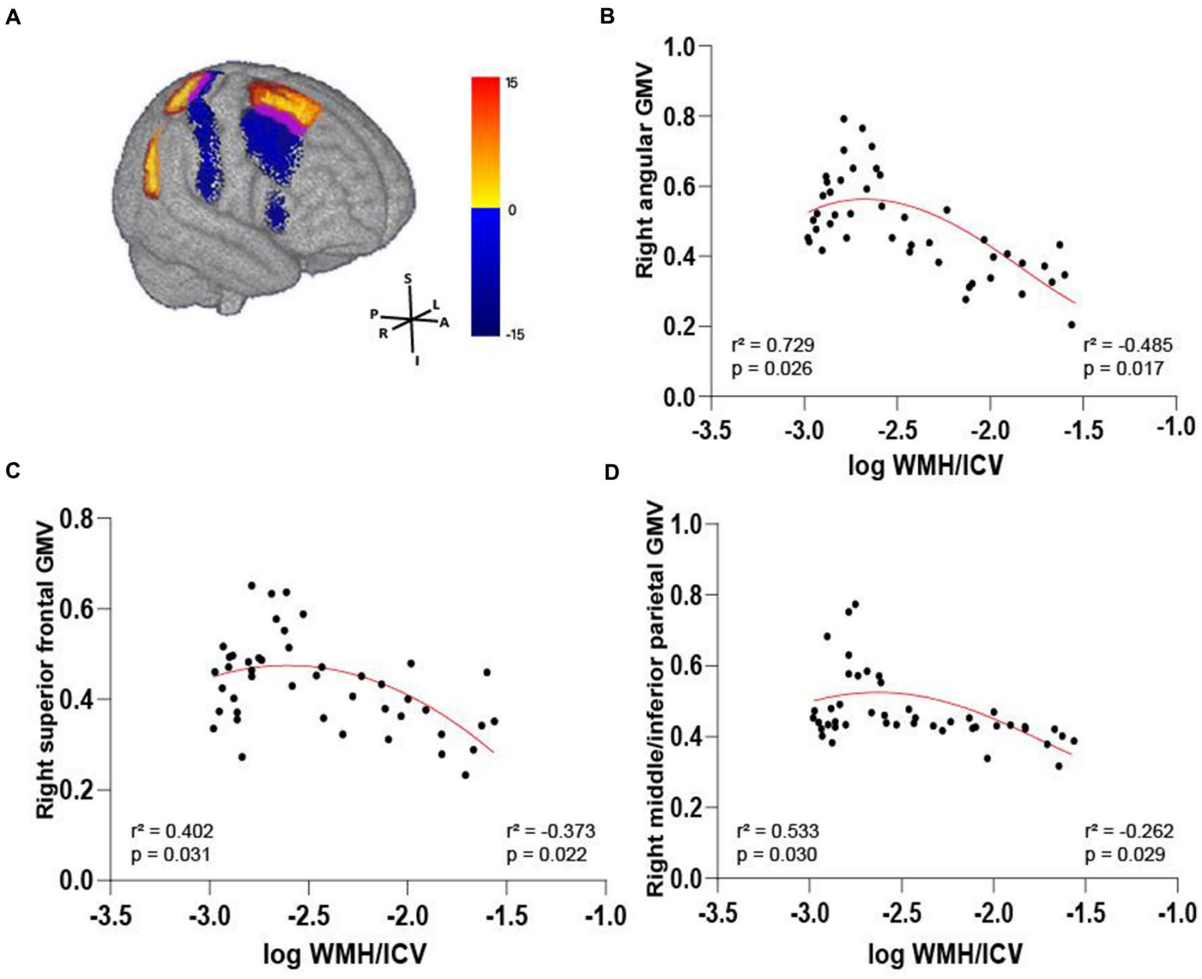
Volume-dependent differential relationship between grey matter and white matter hyperintensity in AD patients. **(A)** The 3D brain slice shows the positive association between voxel-wise GMV and WMH volume at AD patients with mild WMH in the right frontal–parietal cortex (red-yellow) at AD patients with mild WMH, whereas negative association in the right frontal–parietal cortex regions (blue). Overlapping regions are shown in purple. Results are shown on three dimensional renderings of the MNI template brain at pFWE <0.05. **(B–D)** GMV in the right angular cortex **(B)** was positively related to mild WMH volume. On the other hand, the inverse was observed, with increasing WMH volume relating to lower GMV. Similarly, this trend shows in the right superior frontal cortex **(C)** and the right middle/inferior parietal cortex **(D)**. GMV, grey matter volume; WMH, white matter hyperintensity; ICV, intracranial volume; AD, Alzheimer’s disease; MNI, Montreal Neurological Institute; FWE, familywise error correction.

We further compared WMH volume with frontal and parietal GMV. Only in the AD-miWMH group was WMH volume associated with GMV in the following 3 regions: right angular gyrus, right superior frontal gyrus and right middle/inferior parietal gyrus (pFWE <0.01, minimum cluster size = 100 voxels), see Figure 6. Additionally, WMH volume was negatively associated with GMV primarily in 5 areas of frontoparietal regions involving left superior frontal gyrus, bilateral middle frontal gyrus, bilateral inferior frontal gyrus, left angular gyrus, and right superior parietal gyrus in the AD-miWMH group, as shown in Table 4. However, WMH volume was only negatively associated with GMV in frontoparietal regions in the AD-moWMH group (all pFWE <0.05, minimum cluster size = 100 voxels), as shown in Table 4.

**Table 4:** Associations between WMH and voxel-wise regional GMV in AD groups by FWE correction.

Contrast	Regions	Peak t-statistics	Cluster size	MNI coordinates	Pearson r^2^	p
(A) Negative association between WMH volume and GMV						
AD-miWMH group	Left superior frontal gyrus	4.51	531	−12, 42, 24	−0.429	0.037
	Left middle/inferior frontal gyrus	4.82	287	−40, 44, −12	−0.302	0.021
	Left angular gyrus	5.06	232	−50, −64, 31	−0.263	0.011
	Right middle/inferior frontal gyrus	4.41	316	37, 10, 55	−0.405	0.040
	Right superior parietal gyrus	4.59	475	38, −44, 63	−0.677	0.009
AD-moWMH group	Left superior/medial frontal gyrus	3.77	174	−9, 32, 40	−0.311	0.013
	Left middle/inferior frontal gyrus	4.29	231	−45, 30, 16	−0.427	0.011
	Left inferior parietal gyrus	4.60	492	−39, −58, 49	−0.308	0.021
	Left angular gyrus	3.97	142	−44 -64 32	−0.581	0.033
	Left anterior cingulum	4.82	227	−10, 40, 21	−0.323	0.019
	Right superior frontal gyrus	4.42	422	37, 11, 57	−0.372	0.022
	Right inferior frontal gyrus	4.83	202	46, 20, 31	−0.253	0.027
	Right superior parietal gyrus	5.53	423	31, −47, 67	−0.477	0.010
	Right precuneus	4.04	311	28, −55, 60	−0.380	0.021
	Right angular gyrus	4.61	427	51, −53, 42	−0.485	0.017
	Right inferior parietal gyrus	5.07	383	35, −59, 55	−0.262	0.029
(B) Positive association between WMH volume and GMV						
AD-miWMH group	Right angular gyrus	5.20	322	52, −58, 24	0.729	0.026
	Right superior frontal gyrus	4.13	547	41, 10, 47	0.402	0.031
	Right middle/inferior parietal gyrus	4.68	395	44, −26, 49	0.533	0.030

All voxel-wise analyses controlled for age, gender and cognition. WMH, white matter hyperintensity; GMV, grey matter volume; FWE, familywise error correction.

### 3.5 Regional GMV modulates the relationship between WMH and cognition

**Figure 7:**
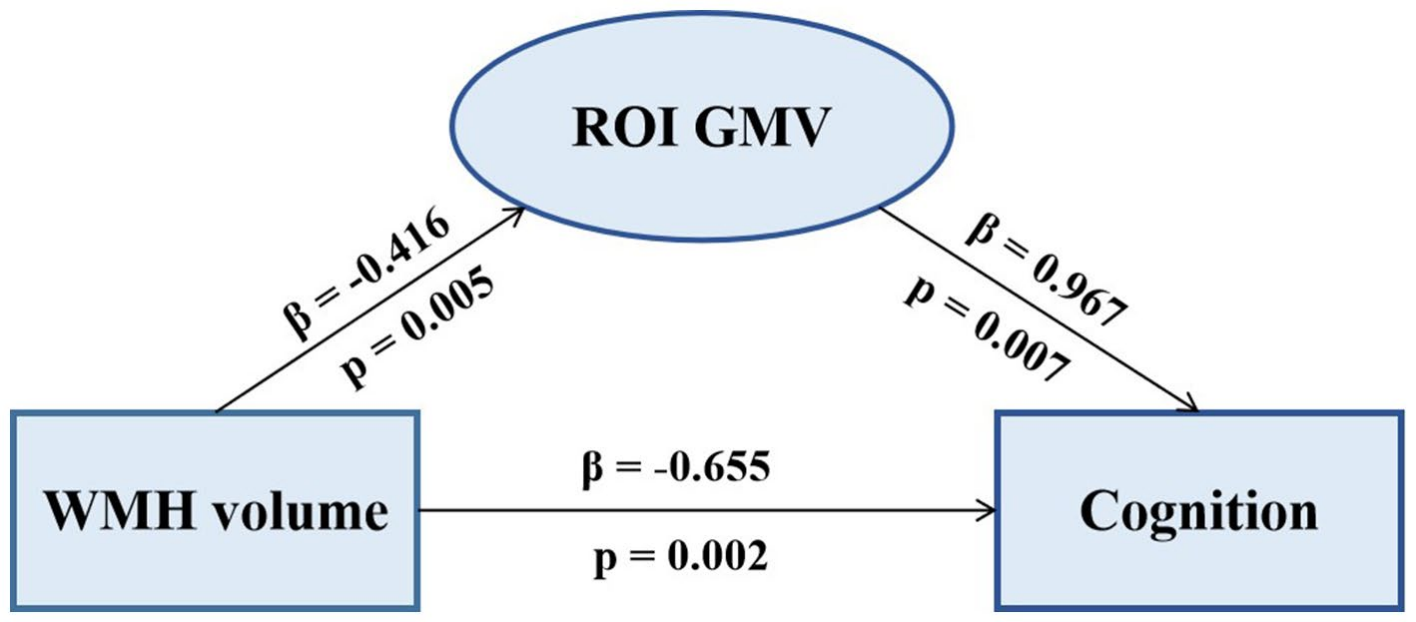
The mediating effect of ROI GMV on the association between WMH volume and cognition. Increased WMH volume was negatively correlated with decreased regional GMV and cognitive dysfunction. Decreased GMV was positively correlated with cognitive dysfunction. ROI, region of interest; GMV, grey matter volume; WMH, white matter hyperintensity.

**Figure 8:**
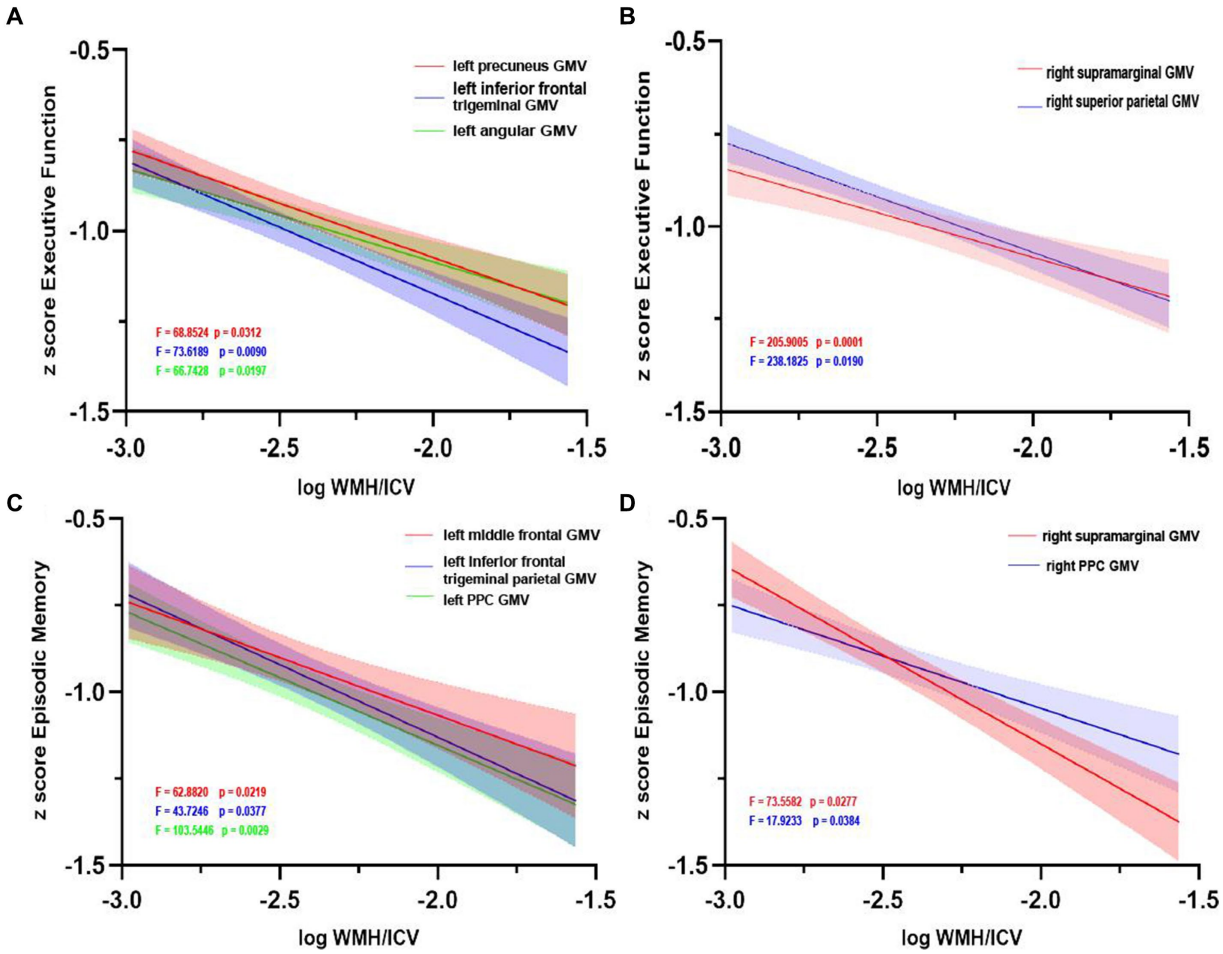
Grey matter volume moderates the relationship between white matter hyperintensity volume and cognition. Left **(A)** and right **(B)** regions of GMV modulated the relationship between executive function impairment and increasing WMH volume. Similarly, left **(C)** and right **(D)** regions of GMV modulated the relationship between episodic memory impairment and increasing WMH volume. GMV, grey matter volume; WMH, white matter hyperintensity; ICV, intracranial volume; PCC, posterior cingulate cortex.

The pattern of mediating effects is illustrated in Figure 7. In the frontoparietal region, moderation analyses after correcting for age and gender revealed that increased WMH volume interaction with these regions of GMV reduction were associated with reduced executive function, i.e., left precuneus (effect size = −0.1638, proportion of relative effect = 66.40%), left inferior frontal triangular gyrus (effect size = −0.1356, proportion of relative effect = 54.96%), left angular gyrus (effect size = −0.1794, proportion of relative effect = 71.11%), right supramarginal gyrus (effect size = −0.1841, proportion of relative effect = 60.38%), and right superior parietal gyrus (effect size = −0.3397, proportion of relative effect = 54.26%), as shown in Figure 8. There were revealed significant moderating effects of these regions of GMV on the association between increased WMH volume and worse episodic memory involving the regions of GMV including: left middle frontal gyrus (effect size = −0.2827, proportion of relative effect = 54.37%), left inferior frontal triangular frontal gyrus (effect size = −0.7714, proportion of relative effect = 59.26%), right supramarginal gyrus (effect size = −0.3593, proportion of relative effect = 78.50%), and bilateral (left/right) posterior parietal cortex (effect size = −0.3121, −0.5237; proportion of relative effect = 56.82, 59.06%), see Figure 8. In comparison of the other cognitive domains, no statistical differences were found between the interaction of increased WMH volume with regional GMV and the cognitive domains. The detailed results of the mediation analysis were listed in Table 5.

**Table 5:** The results of a mediating model test of WMH volume on Z score executive function or episodic memory.

Outcome	R	R^2^	F	Coefficient	T	p
Z score executive function						
Constant	0.6603	0.4360	7.7294	−0.4473	−3.5722	0.0020
Left precuneus GMV	0.9134	0.8344	68.8524	−2.0012	−6.9699	0.0312
Left inferior frontal trigeminal parietal GMV	0.9184	0.8434	73.6189	−1.7483	−4.4204	0.0090
Left angular GMV	0.9134	0.8344	66.7428	−1.9720	−6.8355	0.0197
Right supramarginal GMV	0.9642	0.9378	205.9005	−1.8315	−5.1782	0.0001
Right superior parietal GMV	0.9725	0.9457	238.1825	−1.4434	−3.6072	0.0190
Log WMH				−0.2467	−4.9245	0.0000
Z score episodic memory						
Constant	0.5577	0.3110	4.514 2	−2.2449	−4.3621	0.0001
Left middle frontal GMV	0.4275	0.5044	62.8820	−0.2372	−1.0986	0.0219
Left inferior frontal trigeminal parietal GMV	0.9530	0.7827	43.7246	−2.1839	−7.4019	0.0377
Left PPC GMV	0.7740	0.5079	103.5446	−1.9547	−12.3589	0.0029
Right supramarginal GMV	0.7335	0.6570	73.5582	−1.9032	−14.4238	0.0277
Right PPC GMV	0.7789	0.8328	17.9233	−1.6870	−18.4290	0.0384
Log WMH				−0.2059	−12.3620	0.0187

WMH, white matter hyperintensity volume; GMV, grey matter volume; PPC, posterior parietal cortex.

## 4 Discussion

Our study demonstrates that WMH in Alzheimer’s disease presents with impairment of white matter integrity. The higher WMH volumes in AD indicate more severe damage to WM fiber tracts. We reveal a negative correlation between WMH and grey matter atrophy in frontal–parietal regions in a volume-dependent manner. However, low WMH volume was positively associated with partial regional GMV, and the relationship was not linear. We also demonstrate the correspondence between WMH volume and GMV in cognition. Specifically, the correspondence between increased WMH volume and regional GMV atrophy causes worse episodic memory and execution function. This may have important implications for global or domain-specific cognitive function in AD.

First, at the overall level of fiber bundles, decreased FA in 8 of 20 fiber tracts (bilateral ATR, bilateral CH, right CC, FMi, left IFOF, and left SLF) and 16 fiber tracts apart from the 4 fiber tracts (bilateral UF, FMa, and left ILF) exhibited significantly higher MD in AD patients compared with normal elderly populations. FA decreased in 9 fiber tracts (bilateral ATR, right CST, bilateral CH, right CC, FMa, FMi, and left tSLF) and MD increased in the 13 fiber tracts between AD with higher WMH and AD without WMH. These are predominantly association fiber. The DTI metrics abnormalities of fiber tracts reflect disruption of microstructural WM integrity. We consider WMH to be an external imaging manifestation of subtle microstructural WM tract degeneration. It may suggest the presence of histopathological changes in WM region such as demyelination, Wallerian degeneration, neuron loss, oligodendroglial apoptosis, and gliosis.

Moreover, we observe a mismatch between FA and MD values in the DTI metrics. This may be due to a decrease in tissue density and an increase in water diffusivity while maintaining underlying directional structure, and these outcomes cause an increase in MD when FA remains unchanged (Nir et al., 2013). Excessive accumulation of intracellular and extracellular fluid has been proposed as a possible explanation for the mismatch in DTI metrics changes (Pfefferbaum and Sullivan, 2005). This may be caused by progressive myelin and axonal damage, but also by glial or cellular reactions, for instance, during ongoing inflammatory processes (De et al., 2019). In addition, due to the lack of specificity, the measurement of DTI parameters may be affected by imaging techniques, data processing methods, and individual anatomical differences. Therefore, identifying the underlying neurobiological mechanisms that can explain changes producing differences between DTI metrics is challenging. Further studies are needed to clarify the biological underpinnings of the observations.

Previous studies indicate that total WMH volume was associated with reduced GMV in AD (Vipin et al., 2018). Similarly, we found that negative associations between WMH and GMV have been previously observed. After adjusting for covariates, regional GMV atrophy in the frontal and parietal lobes was observed in AD with combined WMH compared with AD without WMH, whereas hippocampus atrophy was seen in AD patients with higher WMH. This may be the majority of WMH occur in the frontal and parietal subcortical regions (Gouw et al., 2008), causing a reduction in regional grey matter atrophy, especially frontal and parietal-specific structural changes (Ha et al., 2012; Capizzano et al., 2004). We also confirmed that WMH volume was negatively correlated with frontal and parietal GMV and not statistically different with temporal and occipital GMV.

Our study also demonstrates that GMV is decreased in frontal–parietal regions with high WMH in AD, while low WMH volume had an unstable relationship with GMV, positively in the following regions (right angular gyrus, right superior frontal gyrus, and right middle and inferior parietal gyrus), but negatively in other frontal and parietal regions. This positive relationship could be merely stage-dependent, as has been observed not only in mild cognitive impairment patients (Vipin et al., 2021) but also in AD patients. This effect may be a continuous process. The stable and negative relationship between WMH and GMV was observed only after a certain threshold of WMH volume was reached. One possible explanation is that increased GMV indicates the presence of local plasticity (Vipin et al., 2021; Jacobs et al., 2014), i.e., WM damage leads to the WMH increase, which further induced a neuroplastic response by stimulating grey matter adaptations and functional connectivity increases. Wherein the early stage of cortical dysfunction is without no grey matter loss or neurodegeneration, but instead merely compensatory grey matter increase (Reid et al., 2010). Furthermore, increase in regional vasculature could be a compensatory mechanism for ischaemia and increase in the number of synapses comprising synaptogenesis might be a compensatory neuroprotective mechanism underlying increases in grey matter (Zatorre et al., 2012), and such alterations would help the cortex to maintain its function in the presence of cerebrovascular disease (Brickman et al., 2011).

However, there is a stable and widespread negative correlation between WMH and GMV in AD with high WMH volume, which aligns with findings obtained from other studies (Leritz et al., 2011; Tuladhar et al., 2015; Vipin et al., 2018; Habes et al., 2016). One possible mechanism is that on a background of anterograde degeneration (Du et al., 2005), the presence of subcortical WMH leads to structural alterations of the cortex as well as damage to specific white matter tracts connecting these regions (Glickstein and Berlucchi, 2008). Meanwhile, WMH leads to WMH increased impacts on brain structures through increased Aβ deposition or reduced amyloid-beta clearance (Zlokovic, 2011). On the other hand, neurodegenerative alternations such as cortical atrophy as well as tau and amyloid-deposition in the accumulation of WMH via Wallerian degeneration of anatomically connected WM bundles (McAleese et al., 2017; Scott et al., 2016). Our results provide evidence to differential volume-dependent relationships between WMH and brain structure. Thus, studies examining early stages and the disease progression of AD should take into account the presence and influence of WMH as well as their potential interaction with the pathology associated with Aβ deposition and clearance disorders. It may also be possible to track the pattern of cortical atrophy to differentiate between different pathological subtypes of AD (Whitwell et al., 2012).

The influence of WMH on cognition is predominantly considered negative. Previous studies would argue that there is no pair-wise correspondence between WMH and cognition, nor between GMV and cognition. We consider that WMH and GMV co-occur or successive appearance while participate together in the process of cognitive decline. This interaction significantly associated with executive function, and episodic memory. We did not find that WMH moderated effect on GMV existed in cognitive domain involving language, visuospatial, and attention. This effect of WMH on cognition is strengthened by GMV loss in frontoparietal regions (Vipin et al., 2021). Since this correspondence between GMV and WMH on cognition at both low and high WMH volume, GMV may likely be an important moderator. Additionally, the increased WMH volume may cause damage to cholinergic neurotransmitters and interfere with microvascular function due to Aβ deposition (Niwa et al., 2000), further inducing impaired Aβ clearance. The clinical relevance of our study may be that irreversible GMV loss begins when WMH exceeds a certain threshold, and clinical interventions aimed at mitigating GMV loss may be less beneficial. Therefore, monitoring the correspondence between WMH volume, regional GMV, and its impact on cognition during the whole course of AD, as it will provide a window of opportunity for interventions to delay the neurodegenerative process.

Our study has several limitations. First, our study was limited by the cross-sectional study design which prevented us from investigating the effect of WMH on the progression of atrophy patterns in AD progression. In the future, further validation of results using longitudinal datasets is needed. Secondly, we had no information regarding the amyloid and tau status and amyloid positron emission tomography imaging of our participants and were thus unable to assess the influence of these AD biomarkers on the relationship between WMH and brain structure, and between cortical atrophy and WMH volume. Third, due to our moderate sample size, the subjects in our cohort were limited to the elderly Chinese population, and future studies should focus on inclusion of more age groups and ethnicities to evaluate the impact of these variables on the relationship between WMH volume, GMV, and cognition. Fourth, we could only analyze the central portion of WM fibers, so we cannot exclude the possibility of the existence of other portions significantly associated with cognitive decline.

## 5 Conclusion

In conclusion, we confirm WM integrity damage is more in AD patients with higher WMH volume. Differential changes in DTI metrics may be caused by progressive myelin and axonal damage. This changes in DTI metrics need to be further studied. There was a negative correlation between WMH and grey matter atrophy in frontal–parietal regions in a volume-dependent manner. This study indicate the correspondence between WMH volume and GMV in cognition, and GMV being a key modulator between WMH and cognition in AD. Early determination of this relationship and the pattern of cortical atrophy provides an important opportunity to identify subtypes of AD and interventions to delay neurodegenerative changes.

## Data Availability

The original contributions presented in the study are included in the article/Supplementary material, further inquiries can be directed to the corresponding author.
