# Supplementary material for: Correspondence between white matter hyperintensities and regional grey matter volumes in Alzheimer's disease
Source: Front Aging Neurosci. 2024 Sep 16;16:1429098. doi: 10.3389/fnagi.2024.1429098 (PMC11439820; doi:10.3389/fnagi.2024.1429098)
Supplement: Supplementary file 2 [file Data_Sheet_2.doc]

| **描述** | | | | | | | | | |
| --- | --- | --- | --- | --- | --- | --- | --- | --- | --- |
|  | | 个案数 | 平均值 | 标准 偏差 | 标准 错误 | 平均值的 95% 置信区间 | | 最小值 | 最大值 |
|  |  |  |  |  |  | 下限 | 上限 |  |  |
| Age | HC | 28 | 76.68 | 8.924 | 1.242 | 74.24 | 79.33 | 66 | 90 |
|  | AD-nonWMH | 29 | 74.06 | 7.917 | 1.743 | 74.84 | 81.98 | 64 | 94 |
|  | AD-miWMH | 25 | 78.02 | 6.814 | 1.669 | 80.84 | 87.72 | 68 | 97 |
|  | AD-moWMH | 20 | 79.78 | 9.932 | 2.058 | 78.44 | 87.06 | 66 | 93 |
|  | 总计 | 102 | 77.25 | 8.833 | .875 | 78.52 | 81.99 | 64 | 97 |
| Y of Aducation | HC | 28 | 11.706 | 2.0326 | .4767 | 10.147 | 12.103 | 6.0 | 15.0 |
|  | AD-nonWMH | 29 | 12.014 | 1.7933 | .5038 | 10.295 | 12.360 | 6.0 | 16.0 |
|  | AD-miWMH | 25 | 11.320 | 1.9646 | .5669 | 9.410 | 11.750 | 6.0 | 16.0 |
|  | AD-moWMH | 20 | 11.455 | 2.0369 | .5136 | 9.150 | 11.300 | 7.0 | 16.0 |
|  | 总计 | 102 | 10.873 | 2.6125 | .2587 | 10.359 | 11.386 | 6.0 | 16.0 |
| MMSE | HC | 28 | 29.16 | .312 | .146 | 28.88 | 29.48 | 28 | 30 |
|  | AD-nonWMH | 29 | 22.53 | .443 | .346 | 22.84 | 24.26 | 20 | 26 |
|  | AD-miWMH | 25 | 21.49 | .971 | .374 | 21.63 | 23.17 | 19 | 26 |
|  | AD-moWMH | 20 | 18.32 | 1.881 | .499 | 17.81 | 19.89 | 16 | 24 |
|  | 总计 | 102 | 23.89 | 1.032 | .399 | 23.10 | 24.68 | 16 | 30 |
| MoCA-BJ | HC | 28 | 26.77 | .392 | .194 | 25.96 | 26.76 | 25 | 28 |
|  | AD-nonWMH | 29 | 21.77 | .733 | .322 | 21.17 | 22.49 | 18 | 25 |
|  | AD-miWMH | 25 | 20.95 | .892 | .346 | 19.29 | 20.71 | 17 | 23 |
|  | AD-moWMH | 20 | 17.63 | .921 | .380 | 16.70 | 18.30 | 13 | 20 |
|  | 总计 | 102 | 21.77 | .549 | .351 | 21.08 | 22.47 | 13 | 28 |
| HAMD | HC | 28 | 2.07 | .274 | .241 | .58 | 1.57 | 0 | 4 |
|  | AD-nonWMH | 29 | 2.02 | .441 | .403 | 1.17 | 2.83 | 0 | 8 |
|  | AD-miWMH | 25 | 2.37 | .377 | .283 | .98 | 2.14 | 0 | 4 |
|  | AD-moWMH | 20 | 2.77 | .724 | .444 | 1.52 | 3.38 | 0 | 5 |
|  | 总计 | 102 | 2.63 | .592 | .177 | 1.37 | 2.08 | 0 | 8 |
| HAMA | HC | 28 | 2.33 | .193 | .242 | .86 | 1.85 | 0 | 3 |
|  | AD-nonWMH | 29 | 1.95 | .765 | .532 | .84 | 3.02 | 0 | 3 |
|  | AD-miWMH | 25 | 2.63 | .724 | .271 | .44 | 1.56 | 0 | 4 |
|  | AD-moWMH | 20 | 2.55 | .436 | .453 | 1.05 | 2.95 | 0 | 4 |
|  | 总计 | 102 | 2.32 | .553 | .200 | 1.16 | 1.96 | 0 | 4 |

| **描述-Composition Z scores of each cognitive domain** | | | | | | | | | |
| --- | --- | --- | --- | --- | --- | --- | --- | --- | --- |
|  | | 个案数 | 平均值 | 标准 偏差 | 标准 错误 | 平均值的 95% 置信区间 | | 最小值 | 最大值 |
|  |  |  |  |  |  | 下限 | 上限 |  |  |
| Z-Episodic Memory | HC | 28 | .471 | .121 | .117 | .370 | .497 | .336 | .503 |
|  | AD-nonWMH | 29 | -.470 | .368 | .283 | -.892 | -.144 | -.842 | -.172 |
|  | AD-miWMH | 25 | -.793 | .552 | .382 | -1.040 | -.442 | -1.343 | -.247 |
|  | AD-moWMH | 20 | -1.247 | .488 | .211 | -1.581 | -.693 | -1.740 | -.802 |
|  | 总计 | 102 | -.732 | .323 | .277 | -1.581 | .497 | -1.740 | .503 |
| Z-Executive Function | HC | 28 | .451 | .212 | .187 | .292 | .507 | .237 | .626 |
|  | AD-nonWMH | 29 | -.622 | .328 | .272 | -.882 | -.402 | -.913 | -.299 |
|  | AD-miWMH | 25 | -.883 | .274 | .192 | -1.032 | -.741 | -1.155 | -.688 |
|  | AD-moWMH | 20 | -1.094 | .342 | .322 | -1.270 | -.902 | -1.433 | -.752 |
|  | 总计 | 102 | -.811 | .297 | .258 | -1.270 | .507 | -1.433 | .626 |
| Z-Language | HC | 28 | .293 | .157 | .146 | .150 | .376 | .133 | .446 |
|  | AD-nonWMH | 29 | -.822 | .301 | .346 | -1.072 | -.603 | -1.122 | -.527 |
|  | AD-miWMH | 25 | -.767 | .288 | .194 | -.971 | -.559 | -1.062 | -.483 |
|  | AD-moWMH | 20 | -.891 | .307 | .233 | -1.055 | -.647 | -1.203 | -.582 |
|  | 总计 | 102 | -.656 | .244 | .257 | -1.072 | .376 | -1.203 | .446 |
| Z-Visuospatial Function | HC | 28 | .192 | .108 | .112 | .103 | .227 | .082 | .297 |
|  | AD-nonWMH | 29 | -.517 | .383 | .272 | -.817 | -.353 | -.899 | -.291 |
|  | AD-miWMH | 25 | -.573 | .327 | .283 | -.823 | -.330 | -.903 | -.240 |
|  | AD-moWMH | 20 | -.551 | .446 | .380 | -.913 | -.277 | -1.007 | -.103 |
|  | 总计 | 102 | -.429 | .377 | .331 | -.913 | .227 | -1.007 | .297 |
| Z-Attention | HC | 28 | .212 | .138 | .110 | .129 | .301 | .091 | .347 |
|  | AD-nonWMH | 29 | -.330 | .151 | .113 | -.442 | -.191 | -.476 | -.163 |
|  | AD-miWMH | 25 | -.359 | .202 | .199 | -.521 | -.293 | -.561 | -.202 |
|  | AD-moWMH | 20 | -.352 | .218 | .173 | -.497 | -.188 | -.553 | -.129 |
|  | 总计 | 102 | -.288 | .193 | .177 | -.521 | .301 | -.561 | .347 |

| **方差齐性检验** | | | | | |
| --- | --- | --- | --- | --- | --- |
|  | | 莱文统计 | 自由度 1 | 自由度 2 | 显著性 |
| Age | 基于平均值 | 2.044 | 3 | 98 | .113 |
|  | 基于中位数 | 1.479 | 3 | 98 | .225 |
|  | 基于中位数并具有调整后自由度 | 1.479 | 3 | 84.603 | .226 |
|  | 基于剪除后平均值 | 1.982 | 3 | 98 | .122 |
| Y of Aducation | 基于平均值 | .645 | 3 | 98 | .588 |
|  | 基于中位数 | .509 | 3 | 98 | .677 |
|  | 基于中位数并具有调整后自由度 | .509 | 3 | 97.949 | .677 |
|  | 基于剪除后平均值 | .758 | 3 | 98 | .520 |
| MMSE | 基于平均值 | 7.464 | 3 | 98 | .305 |
|  | 基于中位数 | 5.231 | 3 | 98 | .192 |
|  | 基于中位数并具有调整后自由度 | 5.231 | 3 | 71.096 | .273 |
|  | 基于剪除后平均值 | 7.181 | 3 | 98 | .580 |
| MoCA-BJ | 基于平均值 | 1.968 | 3 | 98 | .124 |
|  | 基于中位数 | 2.249 | 3 | 98 | .087 |
|  | 基于中位数并具有调整后自由度 | 2.249 | 3 | 90.214 | .088 |
|  | 基于剪除后平均值 | 1.958 | 3 | 98 | .125 |
| HAMD | 基于平均值 | 4.848 | 3 | 98 | .337 |
|  | 基于中位数 | 3.142 | 3 | 98 | .159 |
|  | 基于中位数并具有调整后自由度 | 3.142 | 3 | 67.317 | .361 |
|  | 基于剪除后平均值 | 4.831 | 3 | 98 | .094 |
| HAMA | 基于平均值 | 3.868 | 3 | 98 | .122 |
|  | 基于中位数 | 1.934 | 3 | 98 | .129 |
|  | 基于中位数并具有调整后自由度 | 1.934 | 3 | 58.067 | .134 |
|  | 基于剪除后平均值 | 2.910 | 3 | 98 | .308 |

| **ANOVA** | | | | | | |
| --- | --- | --- | --- | --- | --- | --- |
|  | | 平方和 | 自由度 | 均方 | F | 显著性 |
| Age | 组间 | 964.834 | 3 | 321.611 | -2.311 | .633 |
|  | 组内 | 6914.539 | 98 | 70.557 |  |  |
|  | 总计 | 7879.373 | 101 |  |  |  |
| Y of Aducation | 组间 | 18.315 | 3 | 6.105 | 1.306 | .571 |
|  | 组内 | 671.028 | 98 | 6.847 |  |  |
|  | 总计 | 689.343 | 101 |  |  |  |
| MMSE | 组间 | 1349.984 | 3 | 449.995 | -1.997 | .011 |
|  | 组内 | 291.830 | 98 | 2.978 |  |  |
|  | 总计 | 1641.814 | 101 |  |  |  |
| MoCA-BJ | 组间 | 1032.247 | 3 | 344.082 | -2.031 | .017 |
|  | 组内 | 239.567 | 98 | 2.445 |  |  |
|  | 总计 | 1271.814 | 101 |  |  |  |
| HAMD | 组间 | 25.347 | 3 | 8.449 | .837 | .619 |
|  | 组内 | 298.967 | 98 | 3.051 |  |  |
|  | 总计 | 324.314 | 101 |  |  |  |
| HAMA | 组间 | 16.856 | 3 | 5.619 | .892 | .577 |
|  | 组内 | 396.291 | 98 | 4.044 |  |  |
|  | 总计 | 413.147 | 101 |  |  |  |

| **事后检验 多重比较** | | | | | | | |
| --- | --- | --- | --- | --- | --- | --- | --- |
| LSD | | | | | | | |
| 因变量 | (I) group | (J) group | 平均值差值 (I-J) | 标准 错误 | 显著性 | 95% 置信区间 | |
|  |  |  |  |  |  | 下限 | 上限 |
| Age | 1 | 2 | -1.628 | 2.226 | .712 | -6.04 | 2.79 |
|  |  | 3 | -7.494 | 2.311 | .462 | -12.08 | -2.91 |
|  |  | 4 | -5.964 | 2.459 | .297 | -10.84 | -1.08 |
|  | 2 | 1 | 1.628 | 2.226 | .712 | -2.79 | 6.04 |
|  |  | 3 | -5.866 | 2.292 | .302 | -10.42 | -1.32 |
|  |  | 4 | -4.336 | 2.441 | .237 | -9.18 | .51 |
|  | 3 | 1 | 7.494 | 2.311 | .462 | 2.91 | 12.08 |
|  |  | 2 | 5.866 | 2.292 | .302 | 1.32 | 10.42 |
|  |  | 4 | 1.530 | 2.520 | .545 | -3.47 | 6.53 |
|  | 4 | 1 | 5.964 | 2.459 | .297 | 1.08 | 10.84 |
|  |  | 2 | 4.336 | 2.441 | .237 | -.51 | 9.18 |
|  |  | 3 | -1.530 | 2.520 | .545 | -6.53 | 3.47 |
| Y of Aducation | 1 | 2 | -.2026 | .6933 | .831 | -1.578 | 1.173 |
|  |  | 3 | .5450 | .7200 | .799 | -.884 | 1.974 |
|  |  | 4 | .9000 | .7661 | .692 | -.620 | 2.420 |
|  | 2 | 1 | .2026 | .6933 | .831 | -1.173 | 1.578 |
|  |  | 3 | .7476 | .7141 | .553 | -.670 | 2.165 |
|  |  | 4 | 1.1026 | .7606 | .564 | -.407 | 2.612 |
|  | 3 | 1 | -.5450 | .7200 | .799 | -1.974 | .884 |
|  |  | 2 | -.7476 | .7141 | .553 | -2.165 | .670 |
|  |  | 4 | .3550 | .7850 | .652 | -1.203 | 1.913 |
|  | 4 | 1 | -.9000 | .7661 | .692 | -2.420 | .620 |
|  |  | 2 | -1.1026 | .7606 | .564 | -2.612 | .407 |
|  |  | 3 | -.3550 | .7850 | .652 | -1.913 | 1.203 |
| MMSE | 1 | 2 | 5.627^*^ | .457 | .027 | 4.72 | 6.53 |
|  |  | 3 | 6.779^*^ | .475 | .022 | 5.84 | 7.72 |
|  |  | 4 | 10.329^*^ | .505 | .021 | 9.33 | 11.33 |
|  | 2 | 1 | -5.627^*^ | .457 | .027 | -6.53 | -4.72 |
|  |  | 3 | 1.152 | .471 | .071 | .22 | 2.09 |
|  |  | 4 | 4.702^*^ | .502 | .009 | 3.71 | 5.70 |
|  | 3 | 1 | -6.779^*^ | .475 | .022 | -7.72 | -5.84 |
|  |  | 2 | -1.152 | .471 | .071 | -2.09 | -.22 |
|  |  | 4 | 3.550^*^ | .518 | .002 | 2.52 | 4.58 |
|  | 4 | 1 | -10.329^*^ | .505 | .021 | -11.33 | -9.33 |
|  |  | 2 | -4.702^*^ | .502 | .009 | -5.70 | -3.71 |
|  |  | 3 | -3.550^*^ | .518 | .002 | -4.58 | -2.52 |
| MoCA-BJ | 1 | 2 | 4.530^*^ | .414 | .035 | 3.71 | 5.35 |
|  |  | 3 | 6.357^*^ | .430 | .020 | 5.50 | 7.21 |
|  |  | 4 | 8.857^*^ | .458 | .019 | 7.95 | 9.77 |
|  | 2 | 1 | -4.530^*^ | .414 | .035 | -5.35 | -3.71 |
|  |  | 3 | 1.828 | .427 | .088 | .98 | 2.67 |
|  |  | 4 | 4.328^*^ | .454 | .011 | 3.43 | 5.23 |
|  | 3 | 1 | -6.357^*^ | .430 | .020 | -7.21 | -5.50 |
|  |  | 2 | -1.828 | .427 | .088 | -2.67 | -.98 |
|  |  | 4 | 2.500^*^ | .469 | .000 | 1.57 | 3.43 |
|  | 4 | 1 | -8.857^*^ | .458 | .019 | -9.77 | -7.95 |
|  |  | 2 | -4.328^*^ | .454 | .011 | -5.23 | -3.43 |
|  |  | 3 | -2.500^*^ | .469 | .000 | -3.43 | -1.57 |
| HAMD | 1 | 2 | -.929 | .463 | .790 | -1.85 | -.01 |
|  |  | 3 | -.489 | .481 | .787 | -1.44 | .47 |
|  |  | 4 | -1.379 | .511 | .687 | -2.39 | -.36 |
|  | 2 | 1 | .929 | .463 | .790 | .01 | 1.85 |
|  |  | 3 | .440 | .477 | .737 | -.51 | 1.39 |
|  |  | 4 | -.450 | .508 | .582 | -1.46 | .56 |
|  | 3 | 1 | .489 | .481 | .787 | -.47 | 1.44 |
|  |  | 2 | -.440 | .477 | .737 | -1.39 | .51 |
|  |  | 4 | -.890 | .524 | .093 | -1.93 | .15 |
|  | 4 | 1 | 1.379 | .511 | .687 | .36 | 2.39 |
|  |  | 2 | .450 | .508 | .582 | -.56 | 1.46 |
|  |  | 3 | .890 | .524 | .093 | -.15 | 1.93 |
| HAMA | 1 | 2 | -.574 | .533 | .284 | -1.63 | .48 |
|  |  | 3 | .357 | .553 | .520 | -.74 | 1.46 |
|  |  | 4 | -.643 | .589 | .278 | -1.81 | .53 |
|  | 2 | 1 | .574 | .533 | .284 | -.48 | 1.63 |
|  |  | 3 | .931 | .549 | .493 | -.16 | 2.02 |
|  |  | 4 | -.069 | .584 | .577 | -1.23 | 1.09 |
|  | 3 | 1 | -.357 | .553 | .520 | -1.46 | .74 |
|  |  | 2 | -.931 | .549 | .493 | -2.02 | .16 |
|  |  | 4 | -1.000 | .603 | .311 | -2.20 | .20 |
|  | 4 | 1 | .643 | .589 | .278 | -.53 | 1.81 |
|  |  | 2 | .069 | .584 | .577 | -1.09 | 1.23 |
|  |  | 3 | 1.000 | .603 | .311 | -.20 | 2.20 |
| *. 平均值差值的显著性水平为 0.05。 | | | | | | | |

| **ANOVA-Composition Z scores of each cognitive domain** | | | | | | |
| --- | --- | --- | --- | --- | --- | --- |
|  | | 平方和 | 自由度 | 均方 | F | 显著性 |
| Z-Episodic Memory | 组间 | 33.128 | 3 | 11.043 | 33.712 | .021 |
|  | 组内 | 4.310 | 98 | .044 |  |  |
|  | 总计 | 37.438 | 101 |  |  |  |
| Z-Executive Function | 组间 | 35.046 | 3 | 11.682 | 42.392 | .033 |
|  | 组内 | 2.531 | 98 | .026 |  |  |
|  | 总计 | 37.577 | 101 |  |  |  |
| Z-Language | 组间 | 25.582 | 3 | 8.527 | 28.320 | .037 |
|  | 组内 | 1.995 | 98 | .020 |  |  |
|  | 总计 | 27.577 | 101 |  |  |  |
| Z-Visuospatial Function | 组间 | 11.510 | 3 | 3.837 | 16.517 | .029 |
|  | 组内 | 3.698 | 98 | .038 |  |  |
|  | 总计 | 15.208 | 101 |  |  |  |
| Z-Attention | 组间 | 6.207 | 3 | 2.069 | 19.776 | .042 |
|  | 组内 | .974 | 98 | .010 |  |  |
|  | 总计 | 7.181 | 101 |  |  |  |

| **事后检验 多重比较-Composition Z scores of each cognitive domain** | | | | | | | |
| --- | --- | --- | --- | --- | --- | --- | --- |
| LSD | | | | | | | |
| 因变量 | (I) group | (J) group | 平均值差值 (I-J) | 标准 错误 | 显著性 | 95% 置信区间 | |
|  |  |  |  |  |  | 下限 | 上限 |
| Z-Episodic Memory | 1 | 2 | .93725^*^ | .05556 | .027 | .7876 | 1.0869 |
|  |  | 3 | 1.23436^*^ | .05771 | .019 | 1.0790 | 1.3898 |
|  |  | 4 | 1.52136^*^ | .06140 | .000 | 1.3560 | 1.6867 |
|  | 2 | 1 | -.93725^*^ | .05556 | .027 | -1.0869 | -.7876 |
|  |  | 3 | .29710^*^ | .05723 | .033 | .1430 | .4512 |
|  |  | 4 | .58410^*^ | .06096 | .007 | .4200 | .7482 |
|  | 3 | 1 | -1.23436^*^ | .05771 | .019 | -1.3898 | -1.0790 |
|  |  | 2 | -.29710^*^ | .05723 | .033 | -.4512 | -.1430 |
|  |  | 4 | .28700^*^ | .06291 | .029 | .1176 | .4564 |
|  | 4 | 1 | -1.52136^*^ | .06140 | .000 | -1.6867 | -1.3560 |
|  |  | 2 | -.58410^*^ | .06096 | .007 | -.7482 | -.4200 |
|  |  | 3 | -.28700^*^ | .06291 | .029 | -.4564 | -.1176 |
| Z-Executive Function | 1 | 2 | 1.06831^*^ | .04258 | .031 | .9537 | 1.1830 |
|  |  | 3 | 1.32867^*^ | .04422 | .019 | 1.2096 | 1.4478 |
|  |  | 4 | 1.49157^*^ | .04705 | .000 | 1.3649 | 1.6183 |
|  | 2 | 1 | -1.06831^*^ | .04258 | .031 | -1.1830 | -.9537 |
|  |  | 3 | .26036^*^ | .04386 | .027 | .1423 | .3785 |
|  |  | 4 | .42326^*^ | .04671 | .023 | .2975 | .5490 |
|  | 3 | 1 | -1.32867^*^ | .04422 | .019 | -1.4478 | -1.2096 |
|  |  | 2 | -.26036^*^ | .04386 | .027 | -.3785 | -.1423 |
|  |  | 4 | .16290^*^ | .04821 | .035 | .0331 | .2927 |
|  | 4 | 1 | -1.49157^*^ | .04705 | .000 | -1.6183 | -1.3649 |
|  |  | 2 | -.42326^*^ | .04671 | .023 | -.5490 | -.2975 |
|  |  | 3 | -.16290^*^ | .04821 | .035 | -.2927 | -.0331 |
| Z-Language | 1 | 2 | 1.13062^*^ | .03780 | .044 | 1.0288 | 1.2324 |
|  |  | 3 | 1.06506^*^ | .03926 | .041 | .9593 | 1.1708 |
|  |  | 4 | 1.17086^*^ | .04177 | .017 | 1.0584 | 1.2833 |
|  | 2 | 1 | -1.13062^*^ | .03780 | .044 | -1.2324 | -1.0288 |
|  |  | 3 | -.06556 | .03894 | .071 | -.1704 | .0393 |
|  |  | 4 | .04024 | .04147 | .119 | -.0714 | .1519 |
|  | 3 | 1 | -1.06506^*^ | .03926 | .041 | -1.1708 | -.9593 |
|  |  | 2 | .06556 | .03894 | .071 | -.0393 | .1704 |
|  |  | 4 | .10580 | .04280 | .093 | -.0095 | .2211 |
|  | 4 | 1 | -1.17086^*^ | .04177 | .017 | -1.2833 | -1.0584 |
|  |  | 2 | -.04024 | .04147 | .119 | -.1519 | .0714 |
|  |  | 3 | -.10580 | .04280 | .093 | -.2211 | .0095 |
| Z-Visuospatial Function | 1 | 2 | .73427^*^ | .05147 | .035 | .5957 | .8729 |
|  |  | 3 | .76953^*^ | .05345 | .020 | .6256 | .9135 |
|  |  | 4 | .75643^*^ | .05687 | .019 | .6033 | .9096 |
|  | 2 | 1 | -.73427^*^ | .05147 | .035 | -.8729 | -.5957 |
|  |  | 3 | .03526 | .05301 | .088 | -.1075 | .1780 |
|  |  | 4 | .02216 | .05646 | .092 | -.1299 | .1742 |
|  | 3 | 1 | -.76953^*^ | .05345 | .020 | -.9135 | -.6256 |
|  |  | 2 | -.03526 | .05301 | .088 | -.1780 | .1075 |
|  |  | 4 | -.01310 | .05828 | .090 | -.1700 | .1438 |
|  | 4 | 1 | -.75643^*^ | .05687 | .019 | -.9096 | -.6033 |
|  |  | 2 | -.02216 | .05646 | .092 | -.1742 | .1299 |
|  |  | 3 | .01310 | .05828 | .090 | -.1438 | .1700 |
| Z-Attention | 1 | 2 | .54080^*^ | .02641 | .044 | .4697 | .6119 |
|  |  | 3 | .56561^*^ | .02743 | .040 | .4917 | .6395 |
|  |  | 4 | .55271^*^ | .02919 | .035 | .4741 | .6313 |
|  | 2 | 1 | -.54080^*^ | .02641 | .044 | -.6119 | -.4697 |
|  |  | 3 | .02481 | .02721 | .093 | -.0485 | .0981 |
|  |  | 4 | .01191 | .02898 | .077 | -.0661 | .0899 |
|  | 3 | 1 | -.56561^*^ | .02743 | .040 | -.6395 | -.4917 |
|  |  | 2 | -.02481 | .02721 | .093 | -.0981 | .0485 |
|  |  | 4 | -.01290 | .02991 | .311 | -.0934 | .0676 |
|  | 4 | 1 | -.55271^*^ | .02919 | .035 | -.6313 | -.4741 |
|  |  | 2 | -.01191 | .02898 | .077 | -.0899 | .0661 |
|  |  | 3 | .01290 | .02991 | .311 | -.0676 | .0934 |
| *. 平均值差值的显著性水平为 0.05。 | | | | | | | |

| **Group Male/Female独立样本克鲁斯卡尔-沃利斯检验摘要** | |
| --- | --- |
| 总计 N | 5253 |
| 检验统计 | 61.470^a^ |
| 自由度 | 3 |
| 渐进显著性（双侧检验） | .721 |
| a. 检验统计将针对绑定值进行调整。 | |

| **group Male/Female的成对比较** | | | | | |
| --- | --- | --- | --- | --- | --- |
| Sample 1-Sample 2 | 检验统计 | 标准误差 | 标准检验统计 | 显著性 | Adj.显著性^a^ |
| 2-4 | -97.534 | 63.240 | -1.542 | .738 | .357 |
| 2-3 | -121.781 | 54.996 | -2.214 | .161 | .454 |
| 2-1 | 330.187 | 45.272 | 7.293 | .537 | .792 |
| 4-3 | 24.247 | 65.366 | .371 | .711 | .570 |
| 4-1 | 232.653 | 57.425 | 4.051 | .533 | .467 |
| 3-1 | 208.406 | 48.197 | 4.324 | .297 | .553 |
| 每行都检验“样本 1 与样本 2 的分布相同”这一原假设。  显示了渐进显著性（双侧检验）。 显著性水平为 .05。 | | | | | |
| a. 已针对多项检验通过 Bonferroni 校正法调整显著性值。 | | | | | |

| **HBP 独立样本克鲁斯卡尔-沃利斯检验摘要** | |
| --- | --- |
| 总计 N | 5253 |
| 检验统计 | 159.593^a^ |
| 自由度 | 3 |
| 渐进显著性（双侧检验） | .454 |
| a. 检验统计将针对绑定值进行调整。 | |

| **HBP group 的成对比较** | | | | | |
| --- | --- | --- | --- | --- | --- |
| Sample 1-Sample 2 | 检验统计 | 标准误差 | 标准检验统计 | 显著性 | Adj.显著性^a^ |
| 2-4 | -460.408 | 62.502 | -7.366 | .508 | .773 |
| 2-1 | 494.058 | 44.744 | 11.042 | .646 | .520 |
| 2-3 | -603.314 | 54.355 | -11.100 | .293 | .311 |
| 4-1 | 33.650 | 56.755 | .593 | .553 | .454 |
| 4-3 | 142.906 | 64.604 | 2.212 | .027 | .162 |
| 1-3 | -109.256 | 47.635 | -2.294 | .722 | .811 |
| 每行都检验“样本 1 与样本 2 的分布相同”这一原假设。  显示了渐进显著性（双侧检验）。 显著性水平为 .05。 | | | | | |
| a. 已针对多项检验通过 Bonferroni 校正法调整显著性值。 | | | | | |

| **CHD独立样本克鲁斯卡尔-沃利斯检验摘要** | |
| --- | --- |
| 总计 N | 5253 |
| 检验统计 | 55.230^a^ |
| 自由度 | 3 |
| 渐进显著性（双侧检验） | .603 |
| a. 检验统计将针对绑定值进行调整。 | |

| **CHD group 的成对比较** | | | | | |
| --- | --- | --- | --- | --- | --- |
| Sample 1-Sample 2 | 检验统计 | 标准误差 | 标准检验统计 | 显著性 | Adj.显著性^a^ |
| 3-2 | 22.899 | 47.310 | .484 | .628 | .723 |
| 3-1 | 211.520 | 41.461 | 5.102 | .521 | .593 |
| 3-4 | -313.160 | 56.230 | -5.569 | .139 | .277 |
| 2-1 | 188.621 | 38.944 | 4.843 | .483 | .603 |
| 2-4 | -290.261 | 54.401 | -5.336 | .155 | .173 |
| 1-4 | -101.640 | 49.399 | -2.058 | .274 | .299 |
| 每行都检验“样本 1 与样本 2 的分布相同”这一原假设。  显示了渐进显著性（双侧检验）。 显著性水平为 .05。 | | | | | |
| a. 已针对多项检验通过 Bonferroni 校正法调整显著性值。 | | | | | |

| **DM独立样本克鲁斯卡尔-沃利斯检验摘要** | |
| --- | --- |
| 总计 N | 5253 |
| 检验统计 | 103.432^a^ |
| 自由度 | 3 |
| 渐进显著性（双侧检验） | .366 |
| a. 检验统计将针对绑定值进行调整。 | |

| **DM group 的成对比较** | | | | | |
| --- | --- | --- | --- | --- | --- |
| Sample 1-Sample 2 | 检验统计 | 标准误差 | 标准检验统计 | 显著性 | Adj.显著性^a^ |
| 3-2 | 25.138 | 46.656 | .539 | .590 | .195 |
| 3-1 | 29.700 | 40.888 | .726 | .468 | .154 |
| 3-4 | -491.780 | 55.453 | -8.868 | .620 | .572 |
| 2-1 | 4.562 | 38.406 | .119 | .905 | .621 |
| 2-4 | -466.642 | 53.649 | -8.698 | .423 | .366 |
| 1-4 | -462.080 | 48.717 | -9.485 | .401 | .372 |
| 每行都检验“样本 1 与样本 2 的分布相同”这一原假设。  显示了渐进显著性（双侧检验）。 显著性水平为 .05。 | | | | | |
| a. 已针对多项检验通过 Bonferroni 校正法调整显著性值。 | | | | | |

| **Hyperlipidemia 独立样本克鲁斯卡尔-沃利斯检验摘要** | |
| --- | --- |
| 总计 N | 5253 |
| 检验统计 | 101.243^a^ |
| 自由度 | 3 |
| 渐进显著性（双侧检验） | .447 |
| a. 检验统计将针对绑定值进行调整。 | |

| **Hyperlipidemia group 的成对比较** | | | | | |
| --- | --- | --- | --- | --- | --- |
| Sample 1-Sample 2 | 检验统计 | 标准误差 | 标准检验统计 | 显著性 | Adj.显著性^a^ |
| 1-4 | -271.692 | 47.890 | -5.673 | .572 | .622 |
| 1-2 | -288.496 | 37.755 | -7.641 | .773 | .835 |
| 1-3 | -320.776 | 40.195 | -7.981 | .625 | .474 |
| 4-2 | 16.804 | 52.739 | .319 | .553 | .447 |
| 4-3 | 49.084 | 54.513 | .900 | .368 | .283 |
| 2-3 | -32.281 | 45.865 | -.704 | .882 | .937 |
| 每行都检验“样本 1 与样本 2 的分布相同”这一原假设。  显示了渐进显著性（双侧检验）。 显著性水平为 .05。 | | | | | |
| a. 已针对多项检验通过 Bonferroni 校正法调整显著性值。 | | | | | |

| **Smoke独立样本克鲁斯卡尔-沃利斯检验摘要** | |
| --- | --- |
| 总计 N | 5253 |
| 检验统计 | 2.555^a^, |
| 自由度 | 3 |
| 渐进显著性（双侧检验） | .652 |
| a. 检验统计将针对绑定值进行调整。 | |

| **Smoke group 的成对比较** | | | | | |
| --- | --- | --- | --- | --- | --- |
| Sample 1-Sample 2 | 检验统计 | 标准误差 | 标准检验统计 | 显著性 | Adj.显著性^a^ |
| 1-4 | -271.692 | 47.890 | -5.673 | .713 | .787 |
| 1-2 | -288.496 | 37.755 | -7.641 | .383 | .296 |
| 1-3 | -320.776 | 40.195 | -7.981 | .461 | .299 |
| 4-2 | 16.804 | 52.739 | .319 | .750 | .622 |
| 4-3 | 49.084 | 54.513 | .900 | .368 | .291 |
| 2-3 | -32.281 | 45.865 | -.704 | .482 | .503 |
| 每行都检验“样本 1 与样本 2 的分布相同”这一原假设。  显示了渐进显著性（双侧检验）。 显著性水平为 .05。 | | | | | |
| a. 已针对多项检验通过 Bonferroni 校正法调整显著性值。 | | | | | |

**各组纤维束FA值比较**

| **ANOVA-FA值比较** | | | | | | |
| --- | --- | --- | --- | --- | --- | --- |
|  | | 平方和 | 自由度 | 均方 | F | 显著性 |
| ATR-L | 组间 | .102 | 3 | .034 | 99.063 | .000 |
|  | 组内 | .034 | 98 | .000 |  |  |
|  | 总计 | .136 | 101 |  |  |  |
| ATR-R | 组间 | .092 | 3 | .031 | 24.524 | .000 |
|  | 组内 | .123 | 98 | .001 |  |  |
|  | 总计 | .215 | 101 |  |  |  |
| CST-L | 组间 | .145 | 3 | .048 | 1.382 | .533 |
|  | 组内 | .971 | 98 | .214 |  |  |
|  | 总计 | .116 | 101 |  |  |  |
| CST-R | 组间 | .021 | 3 | .007 | 2.807 | .144 |
|  | 组内 | .243 | 98 | .002 |  |  |
|  | 总计 | .264 | 101 |  |  |  |

| **事后比较--各组纤维束FA值多重比较** | | | | | | | | |
| --- | --- | --- | --- | --- | --- | --- | --- | --- |
| 因变量 | | (I) group | (J) group | 平均值差值 (I-J) | 标准 错误 | 显著性 | 95% 置信区间 | |
|  |  |  |  |  |  |  | 下限 | 上限 |
| ATR-L | LSD | 1 | 2 | .018793^*^ | .004914 | .000 | .00904 | .02855 |
|  |  |  | 3 | .055360^*^ | .005104 | .000 | .04523 | .06549 |
|  |  |  | 4 | .085000^*^ | .005430 | .000 | .07422 | .09578 |
|  |  | 2 | 1 | -.018793^*^ | .004914 | .000 | -.02855 | -.00904 |
|  |  |  | 3 | .036567^*^ | .005062 | .000 | .02652 | .04661 |
|  |  |  | 4 | .066207^*^ | .005391 | .000 | .05551 | .07691 |
|  |  | 3 | 1 | -.055360^*^ | .005104 | .000 | -.06549 | -.04523 |
|  |  |  | 2 | -.036567^*^ | .005062 | .000 | -.04661 | -.02652 |
|  |  |  | 4 | .029640^*^ | .005565 | .013 | .01860 | .04068 |
|  |  | 4 | 1 | -.085000^*^ | .005430 | .000 | -.09578 | -.07422 |
|  |  |  | 2 | -.066207^*^ | .005391 | .000 | -.07691 | -.05551 |
|  |  |  | 3 | -.029640^*^ | .005565 | .013 | -.04068 | -.01860 |
| ATR-R | LSD | 1 | 2 | .007557^*^ | .009370 | .009 | -.01104 | .02615 |
|  |  |  | 3 | .046583^*^ | .009732 | .000 | .02727 | .06590 |
|  |  |  | 4 | .077693^*^ | .010355 | .000 | .05714 | .09824 |
|  |  | 2 | 1 | -.007557^*^ | .009370 | .009 | -.02615 | .01104 |
|  |  |  | 3 | .039026 | .009652 | .000 | .01987 | .05818 |
|  |  |  | 4 | .070136^*^ | .010280 | .000 | .04974 | .09054 |
|  |  | 3 | 1 | -.046583^*^ | .009732 | .000 | -.06590 | -.02727 |
|  |  |  | 2 | -.039026 | .009652 | .000 | -.05818 | -.01987 |
|  |  |  | 4 | .031110^*^ | .010610 | .024 | .01005 | .05217 |
|  |  | 4 | 1 | -.077693^*^ | .010355 | .000 | -.09824 | -.05714 |
|  |  |  | 2 | -.070136^*^ | .010280 | .000 | -.09054 | -.04974 |
|  |  |  | 3 | -.031110^*^ | .010610 | .024 | -.05217 | -.01005 |
| CST-L | LSD | 1 | 2 | .013917 | 247.309092 | .422 | -.76293 | .79076 |
|  |  |  | 3 | .023847 | 256.844095 | .792 | -.67490 | .72259 |
|  |  |  | 4 | -.987643 | 273.279639 | .086 | -1.30219 | .32691 |
|  |  | 2 | 1 | -.013917 | 247.309092 | .422 | -.79076 | .76293 |
|  |  |  | 3 | .009930 | 254.746688 | .277 | -.52658 | .54644 |
|  |  |  | 4 | -.001560 | 271.309320 | .084 | -1.40608 | .40296 |
|  |  | 3 | 1 | -.023847 | 256.844095 | .792 | -.72259 | .67490 |
|  |  |  | 2 | -.009930 | 254.746688 | .277 | -.54644 | .52658 |
|  |  |  | 4 | -.011490 | 280.028301 | .094 | -.71854 | .69556 |
|  |  | 4 | 1 | .987643 | 273.279639 | .086 | -.32691 | 1.30219 |
|  |  |  | 2 | .001560 | 271.309320 | .084 | -.40296 | 1.40608 |
|  |  |  | 3 | .011490 | 280.028301 | .094 | -.69556 | .71854 |
| CST-R | LSD | 1 | 2 | .031416 | .013186 | .079 | .00525 | .05758 |
|  |  |  | 3 | .030311 | .013695 | .219 | .00313 | .05749 |
|  |  |  | 4 | .034521 | .014571 | .830 | .00561 | .06344 |
|  |  | 2 | 1 | -.031416 | .013186 | .079 | -.05758 | -.00525 |
|  |  |  | 3 | -.001105 | .013583 | .935 | -.02806 | .02585 |
|  |  |  | 4 | .003105^*^ | .014466 | .002 | -.02560 | .03181 |
|  |  | 3 | 1 | -.030311 | .013695 | .219 | -.05749 | -.00313 |
|  |  |  | 2 | .001105 | .013583 | .935 | -.02585 | .02806 |
|  |  |  | 4 | .004210 | .014931 | .779 | -.02542 | .03384 |
|  |  | 4 | 1 | -.034521 | .014571 | .830 | -.06344 | -.00561 |
|  |  |  | 2 | -.003105^*^ | .014466 | .002 | -.03181 | .02560 |
|  |  |  | 3 | -.004210 | .014931 | .779 | -.03384 | .02542 |
| *. 平均值差值的显著性水平为 0.05。 | | | | | | | | |

| **ANOVA- FA值比较** | | | | | | |
| --- | --- | --- | --- | --- | --- | --- |
|  | | 平方和 | 自由度 | 均方 | F | 显著性 |
| CH-L | 组间 | .031 | 3 | .010 | 36.252 | .000 |
|  | 组内 | .028 | 98 | .000 |  |  |
|  | 总计 | .058 | 101 |  |  |  |
| CH-R | 组间 | .022 | 3 | .007 | 21.030 | .000 |
|  | 组内 | .035 | 98 | .000 |  |  |
|  | 总计 | .057 | 101 |  |  |  |
| CC-L | 组间 | .012 | 3 | .004 | 22.921 | .126 |
|  | 组内 | .018 | 98 | .000 |  |  |
|  | 总计 | .030 | 101 |  |  |  |
| CC-R | 组间 | .036 | 3 | .012 | 37.359 | .000 |
|  | 组内 | .031 | 98 | .000 |  |  |
|  | 总计 | .067 | 101 |  |  |  |

| **多重比较-FA值比较** | | | | | | | |
| --- | --- | --- | --- | --- | --- | --- | --- |
| LSD | | | | | | | |
| 因变量 | (I) group | (J) group | 平均值差值 (I-J) | 标准 错误 | 显著性 | 95% 置信区间 | |
|  |  |  |  |  |  | 下限 | 上限 |
| CH-L | 1 | 2 | .020325^*^ | .004450 | .000 | .01149 | .02916 |
|  |  | 3 | .025509^*^ | .004621 | .000 | .01634 | .03468 |
|  |  | 4 | .050929^*^ | .004917 | .000 | .04117 | .06069 |
|  | 2 | 1 | -.020325^*^ | .004450 | .000 | -.02916 | -.01149 |
|  |  | 3 | .005183^*^ | .004584 | .006 | -.00391 | .01428 |
|  |  | 4 | .030603^*^ | .004882 | .009 | .02092 | .04029 |
|  | 3 | 1 | -.025509^*^ | .004621 | .000 | -.03468 | -.01634 |
|  |  | 2 | -.005183^*^ | .004584 | .006 | -.01428 | .00391 |
|  |  | 4 | .025420 | .005039 | .072 | .01542 | .03542 |
|  | 4 | 1 | -.050929^*^ | .004917 | .000 | -.06069 | -.04117 |
|  |  | 2 | -.030603^*^ | .004882 | .009 | -.04029 | -.02092 |
|  |  | 3 | -.025420 | .005039 | .072 | -.03542 | -.01542 |
| CH-R | 1 | 2 | .012937^*^ | .004981 | .000 | .00305 | .02282 |
|  |  | 3 | .019259^*^ | .005173 | .000 | .00899 | .02952 |
|  |  | 4 | .043079^*^ | .005504 | .000 | .03216 | .05400 |
|  | 2 | 1 | -.012937^*^ | .004981 | .000 | -.02282 | -.00305 |
|  |  | 3 | .006321 | .005131 | .221 | -.00386 | .01650 |
|  |  | 4 | .030141^*^ | .005464 | .003 | .01930 | .04098 |
|  | 3 | 1 | -.019259^*^ | .005173 | .000 | -.02952 | -.00899 |
|  |  | 2 | -.006321 | .005131 | .221 | -.01650 | .00386 |
|  |  | 4 | .023820 | .005640 | .093 | .01263 | .03501 |
|  | 4 | 1 | -.043079^*^ | .005504 | .000 | -.05400 | -.03216 |
|  |  | 2 | -.030141^*^ | .005464 | .003 | -.04098 | -.01930 |
|  |  | 3 | -.023820 | .005640 | .093 | -.03501 | -.01263 |
| CC-L | 1 | 2 | .011723 | .003546 | .351 | .00469 | .01876 |
|  |  | 3 | .021164 | .003683 | .092 | .01386 | .02847 |
|  |  | 4 | .030464 | .003918 | .088 | .02269 | .03824 |
|  | 2 | 1 | -.011723 | .003546 | .351 | -.01876 | -.00469 |
|  |  | 3 | .009441 | .003653 | .077 | .00219 | .01669 |
|  |  | 4 | .018741 | .003890 | .063 | .01102 | .02646 |
|  | 3 | 1 | -.021164 | .003683 | 092 | -.02847 | -.01386 |
|  |  | 2 | -.009441 | .003653 | .077 | -.01669 | -.00219 |
|  |  | 4 | .009300 | .004015 | .083 | .00133 | .01727 |
|  | 4 | 1 | -.030464 | .003918 | .088 | -.03824 | -.02269 |
|  |  | 2 | -.018741 | .003890 | .063 | -.02646 | -.01102 |
|  |  | 3 | -.009300 | .004015 | .083 | -.01727 | -.00133 |
| CC-R | 1 | 2 | .010959^*^ | .004739 | .003 | .00156 | .02036 |
|  |  | 3 | .040621^*^ | .004921 | .000 | .03086 | .05039 |
|  |  | 4 | .044471^*^ | .005236 | .000 | .03408 | .05486 |
|  | 2 | 1 | -.010959^*^ | .004739 | .003 | -.02036 | -.00156 |
|  |  | 3 | .029662 | .004881 | .077 | .01998 | .03935 |
|  |  | 4 | .033512^*^ | .005198 | .000 | .02320 | .04383 |
|  | 3 | 1 | -.040621^*^ | .004921 | .000 | -.05039 | -.03086 |
|  |  | 2 | -.029662 | .004881 | .077 | -.03935 | -.01998 |
|  |  | 4 | .003850^*^ | .005366 | .035 | -.00680 | .01450 |
|  | 4 | 1 | -.044471^*^ | .005236 | .000 | -.05486 | -.03408 |
|  |  | 2 | -.033512^*^ | .005198 | .000 | -.04383 | -.02320 |
|  |  | 3 | -.003850^*^ | .005366 | .035 | -.01450 | .00680 |
| *. 平均值差值的显著性水平为 0.05。 | | | | | | | |

| **ANOVA-FA值比较** | | | | | | |
| --- | --- | --- | --- | --- | --- | --- |
|  | | 平方和 | 自由度 | 均方 | F | 显著性 |
| FMa | 组间 | .015 | 3 | .005 | 18.469 | .070 |
|  | 组内 | .027 | 98 | .000 |  |  |
|  | 总计 | .042 | 101 |  |  |  |
| FMi | 组间 | .014 | 3 | .005 | 12.244 | .017 |
|  | 组内 | .038 | 98 | .000 |  |  |
|  | 总计 | .052 | 101 |  |  |  |
| IFOF-L | 组间 | .006 | 3 | .002 | 11.119 | .041 |
|  | 组内 | .017 | 98 | .000 |  |  |
|  | 总计 | .023 | 101 |  |  |  |
| IFOF-R | 组间 | .005 | 3 | .002 | 7.386 | .351 |
|  | 组内 | .021 | 98 | .000 |  |  |
|  | 总计 | .025 | 101 |  |  |  |

| **多重比较-FA值比较** | | | | | | | |
| --- | --- | --- | --- | --- | --- | --- | --- |
| LSD | | | | | | | |
| 因变量 | (I) group | (J) group | 平均值差值 (I-J) | 标准 错误 | 显著性 | 95% 置信区间 | |
|  |  |  |  |  |  | 下限 | 上限 |
| FMa | 1 | 2 | .018312 | .004391 | .133 | .00960 | .02703 |
|  |  | 3 | .017436 | .004560 | .507 | .00839 | .02649 |
|  |  | 4 | .035836 | .004852 | .142 | .02621 | .04546 |
|  | 2 | 1 | -.018312 | .004391 | .133 | -.02703 | -.00960 |
|  |  | 3 | -.000876 | .004523 | .847 | -.00985 | .00810 |
|  |  | 4 | .017524^*^ | .004817 | .007 | .00796 | .02708 |
|  | 3 | 1 | -.017436 | .004560 | .507 | -.02649 | -.00839 |
|  |  | 2 | .000876 | .004523 | .847 | -.00810 | .00985 |
|  |  | 4 | .018400 | .004972 | .221 | .00853 | .02827 |
|  | 4 | 1 | -.035836 | .004852 | .142 | -.04546 | -.02621 |
|  |  | 2 | -.017524^*^ | .004817 | .007 | -.02708 | -.00796 |
|  |  | 3 | -.018400 | .004972 | .221 | -.02827 | -.00853 |
| FMi | 1 | 2 | .017076^*^ | .005228 | .002 | .00670 | .02745 |
|  |  | 3 | .015254^*^ | .005429 | .006 | .00448 | .02603 |
|  |  | 4 | .034864^*^ | .005777 | .000 | .02340 | .04633 |
|  | 2 | 1 | -.017076^*^ | .005228 | .002 | -.02745 | -.00670 |
|  |  | 3 | -.001822 | .005385 | .736 | -.01251 | .00886 |
|  |  | 4 | .017788^*^ | .005735 | .003 | .00641 | .02917 |
|  | 3 | 1 | -.015254^*^ | .005429 | .006 | -.02603 | -.00448 |
|  |  | 2 | .001822 | .005385 | .736 | -.00886 | .01251 |
|  |  | 4 | .019610^*^ | .005919 | .031 | .00786 | .03136 |
|  | 4 | 1 | -.034864^*^ | .005777 | .000 | -.04633 | -.02340 |
|  |  | 2 | -.017788^*^ | .005735 | .003 | -.02917 | -.00641 |
|  |  | 3 | -.019610^*^ | .005919 | .031 | -.03136 | -.00786 |
| IFOF-L | 1 | 2 | .010499^*^ | .003537 | .004 | .00348 | .01752 |
|  |  | 3 | .018544^*^ | .003673 | .000 | .01125 | .02583 |
|  |  | 4 | .018514^*^ | .003908 | .000 | .01076 | .02627 |
|  | 2 | 1 | -.010499^*^ | .003537 | .004 | -.01752 | -.00348 |
|  |  | 3 | .008046 | .003643 | .070 | .00082 | .01528 |
|  |  | 4 | .008016 | .003880 | .091 | .00032 | .01572 |
|  | 3 | 1 | -.018544^*^ | .003673 | .000 | -.02583 | -.01125 |
|  |  | 2 | -.008046 | .003643 | .070 | -.01528 | -.00082 |
|  |  | 4 | -.000030 | .004005 | .994 | -.00798 | .00792 |
|  | 4 | 1 | -.018514^*^ | .003908 | .000 | -.02627 | -.01076 |
|  |  | 2 | -.008016 | .003880 | .091 | -.01572 | -.00032 |
|  |  | 3 | .000030 | .004005 | .994 | -.00792 | .00798 |
| IFOF-R | 1 | 2 | .006081 | .003846 | .117 | -.00155 | .01371 |
|  |  | 3 | .017957 | .003994 | .088 | .01003 | .02588 |
|  |  | 4 | .011957 | .004249 | .076 | .00352 | .02039 |
|  | 2 | 1 | -.006081 | .003846 | .117 | -.01371 | .00155 |
|  |  | 3 | .011876 | .003961 | .403 | .00402 | .01974 |
|  |  | 4 | .005876 | .004219 | .167 | -.00250 | .01425 |
|  | 3 | 1 | -.017957 | .003994 | .088 | -.02588 | -.01003 |
|  |  | 2 | -.011876 | .003961 | .403 | -.01974 | -.00402 |
|  |  | 4 | -.006000 | .004354 | .171 | -.01464 | .00264 |
|  | 4 | 1 | -.011957 | .004249 | .076 | -.02039 | -.00352 |
|  |  | 2 | -.005876 | .004219 | .167 | -.01425 | .00250 |
|  |  | 3 | .006000 | .004354 | .171 | -.00264 | .01464 |
| *. 平均值差值的显著性水平为 0.05。 | | | | | | | |

| **ANOVA-FA值比较** | | | | | | |
| --- | --- | --- | --- | --- | --- | --- |
|  | | 平方和 | 自由度 | 均方 | F | 显著性 |
| ILF-L | 组间 | .003 | 3 | .001 | 4.820 | .204 |
|  | 组内 | .021 | 98 | .000 |  |  |
|  | 总计 | .024 | 101 |  |  |  |
| ILF-R | 组间 | .008 | 3 | .003 | 17.355 | .070 |
|  | 组内 | .015 | 98 | .000 |  |  |
|  | 总计 | .023 | 101 |  |  |  |
| SLF-L | 组间 | .021 | 3 | .007 | 23.582 | .019 |
|  | 组内 | .029 | 98 | .000 |  |  |
|  | 总计 | .050 | 101 |  |  |  |
| SLF-R | 组间 | .002 | 3 | .001 | 3.492 | .037 |
|  | 组内 | .020 | 98 | .000 |  |  |
|  | 总计 | .022 | 101 |  |  |  |

| **多重比较-FA值比较** | | | | | | | |
| --- | --- | --- | --- | --- | --- | --- | --- |
| LSD | | | | | | | |
| 因变量 | (I) group | (J) group | 平均值差值 (I-J) | 标准 错误 | 显著性 | 95% 置信区间 | |
|  |  |  |  |  |  | 下限 | 上限 |
| ILF-L | 1 | 2 | .002172 | .003838 | .573 | -.00544 | .00979 |
|  |  | 3 | .007680 | .003986 | .057 | -.00023 | .01559 |
|  |  | 4 | .014850 | .004241 | .091 | .00643 | .02327 |
|  | 2 | 1 | -.002172 | .003838 | .573 | -.00979 | .00544 |
|  |  | 3 | .005508 | .003954 | .167 | -.00234 | .01335 |
|  |  | 4 | .012678 | .004211 | .303 | .00432 | .02103 |
|  | 3 | 1 | -.007680 | .003986 | .057 | -.01559 | .00023 |
|  |  | 2 | -.005508 | .003954 | .167 | -.01335 | .00234 |
|  |  | 4 | .007170 | .004346 | .102 | -.00145 | .01579 |
|  | 4 | 1 | -.014850 | .004241 | .091 | -.02327 | -.00643 |
|  |  | 2 | -.012678 | .004211 | .303 | -.02103 | -.00432 |
|  |  | 3 | -.007170 | .004346 | .102 | -.01579 | .00145 |
| ILF-R | 1 | 2 | .010527 | .003286 | .072 | .00401 | .01705 |
|  |  | 3 | .018766 | .003413 | .088 | .01199 | .02554 |
|  |  | 4 | .023786 | .003632 | .066 | .01658 | .03099 |
|  | 2 | 1 | -.010527 | .003286 | .072 | -.01705 | -.00401 |
|  |  | 3 | .008239^*^ | .003385 | .017 | .00152 | .01496 |
|  |  | 4 | .013259 | .003605 | .059 | .00610 | .02041 |
|  | 3 | 1 | -.018766 | .003413 | .088 | -.02554 | -.01199 |
|  |  | 2 | -.008239^*^ | .003385 | .017 | -.01496 | -.00152 |
|  |  | 4 | .005020 | .003721 | .180 | -.00236 | .01240 |
|  | 4 | 1 | -.023786 | .003632 | .066 | -.03099 | -.01658 |
|  |  | 2 | -.013259 | .003605 | .059 | -.02041 | -.00610 |
|  |  | 3 | -.005020 | .003721 | .180 | -.01240 | .00236 |
| SLF-L | 1 | 2 | .017783^*^ | .004552 | .000 | .00875 | .02682 |
|  |  | 3 | .033394^*^ | .004728 | .000 | .02401 | .04278 |
|  |  | 4 | .035914^*^ | .005030 | .000 | .02593 | .04590 |
|  | 2 | 1 | -.017783^*^ | .004552 | .000 | -.02682 | -.00875 |
|  |  | 3 | .015611 | .004689 | .131 | .00631 | .02492 |
|  |  | 4 | .018131 | .004994 | .079 | .00822 | .02804 |
|  | 3 | 1 | -.033394^*^ | .004728 | .000 | -.04278 | -.02401 |
|  |  | 2 | -.015611 | .004689 | .131 | -.02492 | -.00631 |
|  |  | 4 | .002520 | .005154 | .626 | -.00771 | .01275 |
|  | 4 | 1 | -.035914^*^ | .005030 | .000 | -.04590 | -.02593 |
|  |  | 2 | -.018131 | .004994 | .079 | -.02804 | -.00822 |
|  |  | 3 | -.002520 | .005154 | .626 | -.01275 | .00771 |
| SLF-R | 1 | 2 | .010469 | .003738 | .216 | .00305 | .01789 |
|  |  | 3 | .008887 | .003882 | .094 | .00118 | .01659 |
|  |  | 4 | .010757 | .004130 | .071 | .00256 | .01895 |
|  | 2 | 1 | -.010469 | .003738 | .216 | -.01789 | -.00305 |
|  |  | 3 | -.001582 | .003850 | .682 | -.00922 | .00606 |
|  |  | 4 | .000288 | .004101 | .944 | -.00785 | .00843 |
|  | 3 | 1 | -.008887 | .003882 | .094 | -.01659 | -.00118 |
|  |  | 2 | .001582 | .003850 | .682 | -.00606 | .00922 |
|  |  | 4 | .001870^*^ | .004232 | .040 | -.00653 | .01027 |
|  | 4 | 1 | -.010757 | .004130 | .071 | -.01895 | -.00256 |
|  |  | 2 | -.000288 | .004101 | .944 | -.00843 | .00785 |
|  |  | 3 | -.001870^*^ | .004232 | .040 | -.01027 | .00653 |
| *. 平均值差值的显著性水平为 0.05。 | | | | | | | |

| **ANOVA-FA值比较** | | | | | | |
| --- | --- | --- | --- | --- | --- | --- |
|  | | 平方和 | 自由度 | 均方 | F | 显著性 |
| UF-L | 组间 | .007 | 3 | .002 | 10.051 | .660 |
|  | 组内 | .023 | 98 | .000 |  |  |
|  | 总计 | .030 | 101 |  |  |  |
| UF-R | 组间 | .004 | 3 | .001 | 6.947 | .177 |
|  | 组内 | .017 | 98 | .000 |  |  |
|  | 总计 | .020 | 101 |  |  |  |
| tSLF-L | 组间 | .004 | 3 | .001 | 1.849 | .008 |
|  | 组内 | .067 | 98 | .001 |  |  |
|  | 总计 | .071 | 101 |  |  |  |
| tSLF-R | 组间 | .006 | 3 | .002 | 4.168 | .143 |
|  | 组内 | .045 | 98 | .000 |  |  |
|  | 总计 | .051 | 101 |  |  |  |

| **多重比较-FA值比较** | | | | | | | |
| --- | --- | --- | --- | --- | --- | --- | --- |
| LSD | | | | | | | |
| 因变量 | (I) group | (J) group | 平均值差值 (I-J) | 标准 错误 | 显著性 | 95% 置信区间 | |
|  |  |  |  |  |  | 下限 | 上限 |
| UF-L | 1 | 2 | .009182 | .004023 | .225 | .00120 | .01717 |
|  |  | 3 | .016686 | .004178 | .323 | .00839 | .02498 |
|  |  | 4 | .022536 | .004445 | .198 | .01371 | .03136 |
|  | 2 | 1 | -.009182 | .004023 | .225 | -.01717 | -.00120 |
|  |  | 3 | .007503 | .004144 | .273 | -.00072 | .01573 |
|  |  | 4 | .013353 | .004413 | .425 | .00460 | .02211 |
|  | 3 | 1 | -.016686 | .004178 | .323 | -.02498 | -.00839 |
|  |  | 2 | -.007503 | .004144 | .273 | -.01573 | .00072 |
|  |  | 4 | .005850 | .004555 | .202 | -.00319 | .01489 |
|  | 4 | 1 | -.022536 | .004445 | .198 | -.03136 | -.01371 |
|  |  | 2 | -.013353 | .004413 | .425 | -.02211 | -.00460 |
|  |  | 3 | -.005850 | .004555 | .202 | -.01489 | .00319 |
| UF-R | 1 | 2 | .003533 | .003458 | .309 | -.00333 | .01039 |
|  |  | 3 | .012864 | .003591 | .431 | .00574 | .01999 |
|  |  | 4 | .013964 | .003821 | .220 | .00638 | .02155 |
|  | 2 | 1 | -.003533 | .003458 | .309 | -.01039 | .00333 |
|  |  | 3 | .009331 | .003562 | .177 | .00226 | .01640 |
|  |  | 4 | .010431 | .003793 | .287 | .00290 | .01796 |
|  | 3 | 1 | -.012864 | .003591 | .431 | -.01999 | -.00574 |
|  |  | 2 | -.009331 | .003562 | .177 | -.01640 | -.00226 |
|  |  | 4 | .001100 | .003915 | .779 | -.00667 | .00887 |
|  | 4 | 1 | -.013964 | .003821 | .220 | -.02155 | -.00638 |
|  |  | 2 | -.010431 | .003793 | .287 | -.01796 | -.00290 |
|  |  | 3 | -.001100 | .003915 | .779 | -.00887 | .00667 |
| tSLF-L | 1 | 2 | .007432 | .006938 | .287 | -.00634 | .02120 |
|  |  | 3 | .015176 | .007206 | .098 | .00088 | .02948 |
|  |  | 4 | .014136 | .007667 | .166 | -.00108 | .02935 |
|  | 2 | 1 | -.007432 | .006938 | .287 | -.02120 | .00634 |
|  |  | 3 | .007743 | .007147 | .281 | -.00644 | .02193 |
|  |  | 4 | .006703^*^ | .007611 | .008 | -.00840 | .02181 |
|  | 3 | 1 | -.015176 | .007206 | .098 | -.02948 | -.00088 |
|  |  | 2 | -.007743 | .007147 | .281 | -.02193 | .00644 |
|  |  | 4 | -.001040^*^ | .007856 | .025 | -.01663 | .01455 |
|  | 4 | 1 | -.014136 | .007667 | .166 | -.02935 | .00108 |
|  |  | 2 | -.006703^*^ | .007611 | .008 | -.02181 | .00840 |
|  |  | 3 | .001040^*^ | .007856 | .025 | -.01455 | .01663 |
| tSLF-R | 1 | 2 | .012261 | .005682 | .093 | .00098 | .02354 |
|  |  | 3 | .018371 | .005901 | .112 | .00666 | .03008 |
|  |  | 4 | .018021 | .006279 | .185 | .00556 | .03048 |
|  | 2 | 1 | -.012261 | .005682 | .093 | -.02354 | -.00098 |
|  |  | 3 | .006110 | .005853 | .299 | -.00550 | .01773 |
|  |  | 4 | .005760 | .006234 | .358 | -.00661 | .01813 |
|  | 3 | 1 | -.018371 | .005901 | .112 | -.03008 | -.00666 |
|  |  | 2 | -.006110 | .005853 | .299 | -.01773 | .00550 |
|  |  | 4 | -.000350 | .006434 | .957 | -.01312 | .01242 |
|  | 4 | 1 | -.018021 | .006279 | .185 | -.03048 | -.00556 |
|  |  | 2 | -.005760 | .006234 | .358 | -.01813 | .00661 |
|  |  | 3 | .000350 | .006434 | .957 | -.01242 | .01312 |
| *. 平均值差值的显著性水平为 0.05。 | | | | | | | |

**各组纤维束MD值比较**

| **ANOVA-MD值比较** | | | | | | |
| --- | --- | --- | --- | --- | --- | --- |
|  | | 平方和 | 自由度 | 均方 | F | 显著性 |
| ATR-L | 组间 | .000 | 3 | .000 | 56.817 | .000 |
|  | 组内 | .000 | 98 | .000 |  |  |
|  | 总计 | .000 | 101 |  |  |  |
| ATR-R | 组间 | .000 | 3 | .000 | 41.430 | .000 |
|  | 组内 | .000 | 98 | .000 |  |  |
|  | 总计 | .000 | 101 |  |  |  |
| CST-L | 组间 | .000 | 3 | .000 | 48.956 | .000 |
|  | 组内 | .000 | 98 | .000 |  |  |
|  | 总计 | .000 | 101 |  |  |  |
| CST-R | 组间 | .000 | 3 | .000 | 30.585 | .000 |
|  | 组内 | .000 | 98 | .000 |  |  |
|  | 总计 | .000 | 101 |  |  |  |

| **多重比较-MD值比较** | | | | | | | |
| --- | --- | --- | --- | --- | --- | --- | --- |
| LSD | | | | | | | |
| 因变量 | (I) group | (J) group | 平均值差值 (I-J) | 标准 错误 | 显著性 | 95% 置信区间 | |
|  |  |  |  |  |  | 下限 | 上限 |
| ATR-L | 1 | 2 | -.000097956^*^ | .000015475 | .000 | -.00012867 | -.00006725 |
|  |  | 3 | -.000167409^*^ | .000016071 | .000 | -.00019930 | -.00013552 |
|  |  | 4 | -.000198479^*^ | .000017100 | .000 | -.00023241 | -.00016454 |
|  | 2 | 1 | .000097956^*^ | .000015475 | .000 | .00006725 | .00012867 |
|  |  | 3 | -.000069452^*^ | .000015940 | .000 | -.00010109 | -.00003782 |
|  |  | 4 | -.000100522^*^ | .000016977 | .000 | -.00013421 | -.00006683 |
|  | 3 | 1 | .000167409^*^ | .000016071 | .000 | .00013552 | .00019930 |
|  |  | 2 | .000069452^*^ | .000015940 | .000 | .00003782 | .00010109 |
|  |  | 4 | -.000031070^*^ | .000017522 | .039 | -.00006584 | .00000370 |
|  | 4 | 1 | .000198479^*^ | .000017100 | .000 | .00016454 | .00023241 |
|  |  | 2 | .000100522^*^ | .000016977 | .000 | .00006683 | .00013421 |
|  |  | 3 | .000031070^*^ | .000017522 | .039 | -.00000370 | .00006584 |
| ATR-R | 1 | 2 | -.000083017^*^ | .000015755 | .000 | -.00011428 | -.00005175 |
|  |  | 3 | -.000147580^*^ | .000016363 | .000 | -.00018005 | -.00011511 |
|  |  | 4 | -.000170450^*^ | .000017410 | .000 | -.00020500 | -.00013590 |
|  | 2 | 1 | .000083017^*^ | .000015755 | .000 | .00005175 | .00011428 |
|  |  | 3 | -.000064563^*^ | .000016229 | .000 | -.00009677 | -.00003236 |
|  |  | 4 | -.000087433^*^ | .000017284 | .000 | -.00012173 | -.00005313 |
|  | 3 | 1 | .000147580^*^ | .000016363 | .000 | .00011511 | .00018005 |
|  |  | 2 | .000064563^*^ | .000016229 | .000 | .00003236 | .00009677 |
|  |  | 4 | -.000022870^*^ | .000017840 | .023 | -.00005827 | .00001253 |
|  | 4 | 1 | .000170450^*^ | .000017410 | .000 | .00013590 | .00020500 |
|  |  | 2 | .000087433^*^ | .000017284 | .000 | .00005313 | .00012173 |
|  |  | 3 | .000022870^*^ | .000017840 | .023 | -.00001253 | .00005827 |
| CST-L | 1 | 2 | -.000033512^*^ | .000009524 | .001 | -.00005241 | -.00001461 |
|  |  | 3 | -.000056857^*^ | .000009891 | .000 | -.00007649 | -.00003723 |
|  |  | 4 | -.000124557^*^ | .000010524 | .000 | -.00014544 | -.00010367 |
|  | 2 | 1 | .000033512^*^ | .000009524 | .001 | .00001461 | .00005241 |
|  |  | 3 | -.000023345 | .000009810 | .079 | -.00004281 | -.00000388 |
|  |  | 4 | -.000091045^*^ | .000010448 | .000 | -.00011178 | -.00007031 |
|  | 3 | 1 | .000056857^*^ | .000009891 | .000 | .00003723 | .00007649 |
|  |  | 2 | .000023345 | .000009810 | .079 | .00000388 | .00004281 |
|  |  | 4 | -.000067700^*^ | .000010784 | .000 | -.00008910 | -.00004630 |
|  | 4 | 1 | .000124557^*^ | .000010524 | .000 | .00010367 | .00014544 |
|  |  | 2 | .000091045^*^ | .000010448 | .000 | .00007031 | .00011178 |
|  |  | 3 | .000067700^*^ | .000010784 | .000 | .00004630 | .00008910 |
| CST-R | 1 | 2 | -.000050313^*^ | .000008392 | .000 | -.00006697 | -.00003366 |
|  |  | 3 | -.000067871^*^ | .000008716 | .000 | -.00008517 | -.00005058 |
|  |  | 4 | -.000077871^*^ | .000009273 | .000 | -.00009627 | -.00005947 |
|  | 2 | 1 | .000050313^*^ | .000008392 | .000 | .00003366 | .00006697 |
|  |  | 3 | -.000017559^*^ | .000008645 | .005 | -.00003471 | -.00000040 |
|  |  | 4 | -.000027559^*^ | .000009207 | .003 | -.00004583 | -.00000929 |
|  | 3 | 1 | .000067871^*^ | .000008716 | .000 | .00005058 | .00008517 |
|  |  | 2 | .000017559^*^ | .000008645 | .005 | .00000040 | .00003471 |
|  |  | 4 | -.000010000^*^ | .000009502 | .043 | -.00002886 | .00000886 |
|  | 4 | 1 | .000077871^*^ | .000009273 | .000 | .00005947 | .00009627 |
|  |  | 2 | .000027559^*^ | .000009207 | .003 | .00000929 | .00004583 |
|  |  | 3 | .000010000^*^ | .000009502 | .043 | -.00000886 | .00002886 |
| *. 平均值差值的显著性水平为 0.05。 | | | | | | | |

| **ANOVA-MD值比较** | | | | | | |
| --- | --- | --- | --- | --- | --- | --- |
|  | | 平方和 | 自由度 | 均方 | F | 显著性 |
| CH-L | 组间 | .000 | 3 | .000 | 53.070 | .000 |
|  | 组内 | .000 | 98 | .000 |  |  |
|  | 总计 | .000 | 101 |  |  |  |
| CH-R | 组间 | .000 | 3 | .000 | 33.370 | .000 |
|  | 组内 | .000 | 98 | .000 |  |  |
|  | 总计 | .000 | 101 |  |  |  |
| CC-L | 组间 | .000 | 3 | .000 | 144.727 | .000 |
|  | 组内 | .000 | 98 | .000 |  |  |
|  | 总计 | .000 | 101 |  |  |  |
| CC-R | 组间 | .000 | 3 | .000 | 383.180 | .000 |
|  | 组内 | .000 | 98 | .000 |  |  |
|  | 总计 | .000 | 101 |  |  |  |

| **多重比较-MD值比较** | | | | | | | |
| --- | --- | --- | --- | --- | --- | --- | --- |
| LSD | | | | | | | |
| 因变量 | (I) group | (J) group | 平均值差值 (I-J) | 标准 错误 | 显著性 | 95% 置信区间 | |
|  |  |  |  |  |  | 下限 | 上限 |
| CH-L | 1 | 2 | -.000074855^*^ | .000008812 | .000 | -.00009234 | -.00005737 |
|  |  | 3 | -.000093626^*^ | .000009151 | .000 | -.00011179 | -.00007546 |
|  |  | 4 | -.000106536^*^ | .000009737 | .000 | -.00012586 | -.00008721 |
|  | 2 | 1 | .000074855^*^ | .000008812 | .000 | .00005737 | .00009234 |
|  |  | 3 | -.000018771^*^ | .000009077 | .007 | -.00003678 | -.00000076 |
|  |  | 4 | -.000031681^*^ | .000009667 | .001 | -.00005086 | -.00001250 |
|  | 3 | 1 | .000093626^*^ | .000009151 | .000 | .00007546 | .00011179 |
|  |  | 2 | .000018771^*^ | .000009077 | .007 | .00000076 | .00003678 |
|  |  | 4 | -.000012910 | .000009978 | .199 | -.00003271 | .00000689 |
|  | 4 | 1 | .000106536^*^ | .000009737 | .000 | .00008721 | .00012586 |
|  |  | 2 | .000031681^*^ | .000009667 | .001 | .00001250 | .00005086 |
|  |  | 3 | .000012910 | .000009978 | .199 | -.00000689 | .00003271 |
| CH-R | 1 | 2 | -.000042803^*^ | .000006669 | .000 | -.00005604 | -.00002957 |
|  |  | 3 | -.000055526^*^ | .000006926 | .000 | -.00006927 | -.00004178 |
|  |  | 4 | -.000065086^*^ | .000007369 | .000 | -.00007971 | -.00005046 |
|  | 2 | 1 | .000042803^*^ | .000006669 | .000 | .00002957 | .00005604 |
|  |  | 3 | -.000012723 | .000006869 | .067 | -.00002635 | .00000091 |
|  |  | 4 | -.000022283^*^ | .000007316 | .003 | -.00003680 | -.00000776 |
|  | 3 | 1 | .000055526^*^ | .000006926 | .000 | .00004178 | .00006927 |
|  |  | 2 | .000012723 | .000006869 | .067 | -.00000091 | .00002635 |
|  |  | 4 | -.000009560 | .000007551 | .208 | -.00002454 | .00000542 |
|  | 4 | 1 | .000065086^*^ | .000007369 | .000 | .00005046 | .00007971 |
|  |  | 2 | .000022283^*^ | .000007316 | .003 | .00000776 | .00003680 |
|  |  | 3 | .000009560 | .000007551 | .208 | -.00000542 | .00002454 |
| CC-L | 1 | 2 | -.000044988^*^ | .000005061 | .000 | -.00005503 | -.00003494 |
|  |  | 3 | -.000084603^*^ | .000005256 | .000 | -.00009503 | -.00007417 |
|  |  | 4 | -.000104493^*^ | .000005592 | .000 | -.00011559 | -.00009340 |
|  | 2 | 1 | .000044988^*^ | .000005061 | .000 | .00003494 | .00005503 |
|  |  | 3 | -.000039615^*^ | .000005213 | .000 | -.00004996 | -.00002927 |
|  |  | 4 | -.000059505^*^ | .000005552 | .000 | -.00007052 | -.00004849 |
|  | 3 | 1 | .000084603^*^ | .000005256 | .000 | .00007417 | .00009503 |
|  |  | 2 | .000039615^*^ | .000005213 | .000 | .00002927 | .00004996 |
|  |  | 4 | -.000019890^*^ | .000005730 | .011 | -.00003126 | -.00000852 |
|  | 4 | 1 | .000104493^*^ | .000005592 | .000 | .00009340 | .00011559 |
|  |  | 2 | .000059505^*^ | .000005552 | .000 | .00004849 | .00007052 |
|  |  | 3 | .000019890^*^ | .000005730 | .011 | .00000852 | .00003126 |
| CC-R | 1 | 2 | -.000071777^*^ | .000003813 | .000 | -.00007934 | -.00006421 |
|  |  | 3 | -.000088216^*^ | .000003960 | .000 | -.00009607 | -.00008036 |
|  |  | 4 | -.000138036^*^ | .000004213 | .000 | -.00014640 | -.00012967 |
|  | 2 | 1 | .000071777^*^ | .000003813 | .000 | .00006421 | .00007934 |
|  |  | 3 | -.000016439 | .000003928 | .116 | -.00002423 | -.00000864 |
|  |  | 4 | -.000066259^*^ | .000004183 | .000 | -.00007456 | -.00005796 |
|  | 3 | 1 | .000088216^*^ | .000003960 | .000 | .00008036 | .00009607 |
|  |  | 2 | .000016439 | .000003928 | .116 | .00000864 | .00002423 |
|  |  | 4 | -.000049820^*^ | .000004317 | .030 | -.00005839 | -.00004125 |
|  | 4 | 1 | .000138036^*^ | .000004213 | .000 | .00012967 | .00014640 |
|  |  | 2 | .000066259^*^ | .000004183 | .000 | .00005796 | .00007456 |
|  |  | 3 | .000049820^*^ | .000004317 | .030 | .00004125 | .00005839 |
| *. 平均值差值的显著性水平为 0.05。 | | | | | | | |

| **ANOVA-MD值比较** | | | | | | |
| --- | --- | --- | --- | --- | --- | --- |
|  | | 平方和 | 自由度 | 均方 | F | 显著性 |
| FMa | 组间 | .000 | 3 | .000 | 1.432 | .238 |
|  | 组内 | .000 | 98 | .000 |  |  |
|  | 总计 | .000 | 101 |  |  |  |
| FMi | 组间 | .000 | 3 | .000 | 36.567 | .000 |
|  | 组内 | .000 | 98 | .000 |  |  |
|  | 总计 | .000 | 101 |  |  |  |
| IFOF-L | 组间 | .000 | 3 | .000 | 79.386 | .000 |
|  | 组内 | .000 | 98 | .000 |  |  |
|  | 总计 | .000 | 101 |  |  |  |
| IFOF-R | 组间 | .000 | 3 | .000 | 54.389 | .000 |
|  | 组内 | .000 | 98 | .000 |  |  |
|  | 总计 | .000 | 101 |  |  |  |

| **多重比较-MD值比较** | | | | | | | |
| --- | --- | --- | --- | --- | --- | --- | --- |
| LSD | | | | | | | |
| 因变量 | (I) group | (J) group | 平均值差值 (I-J) | 标准 错误 | 显著性 | 95% 置信区间 | |
|  |  |  |  |  |  | 下限 | 上限 |
| FMa | 1 | 2 | -.000000025 | .000008644 | .998 | -.00001718 | .00001713 |
|  |  | 3 | -.000012194 | .000008978 | .177 | -.00003001 | .00000562 |
|  |  | 4 | -.000014864 | .000009552 | .123 | -.00003382 | .00000409 |
|  | 2 | 1 | .000000025 | .000008644 | .998 | -.00001713 | .00001718 |
|  |  | 3 | -.000012170 | .000008904 | .175 | -.00002984 | .00000550 |
|  |  | 4 | -.000014840 | .000009483 | .121 | -.00003366 | .00000398 |
|  | 3 | 1 | .000012194 | .000008978 | .177 | -.00000562 | .00003001 |
|  |  | 2 | .000012170 | .000008904 | .175 | -.00000550 | .00002984 |
|  |  | 4 | -.000002670 | .000009788 | .786 | -.00002209 | .00001675 |
|  | 4 | 1 | .000014864 | .000009552 | .123 | -.00000409 | .00003382 |
|  |  | 2 | .000014840 | .000009483 | .121 | -.00000398 | .00003366 |
|  |  | 3 | .000002670 | .000009788 | .786 | -.00001675 | .00002209 |
| FMi | 1 | 2 | -.000022339^*^ | .000005402 | .000 | -.00003306 | -.00001162 |
|  |  | 3 | -.000042781^*^ | .000005610 | .000 | -.00005391 | -.00003165 |
|  |  | 4 | -.000057371^*^ | .000005969 | .000 | -.00006922 | -.00004553 |
|  | 2 | 1 | .000022339^*^ | .000005402 | .000 | .00001162 | .00003306 |
|  |  | 3 | -.000020443^*^ | .000005564 | .000 | -.00003148 | -.00000940 |
|  |  | 4 | -.000035033^*^ | .000005926 | .000 | -.00004679 | -.00002327 |
|  | 3 | 1 | .000042781^*^ | .000005610 | .000 | .00003165 | .00005391 |
|  |  | 2 | .000020443^*^ | .000005564 | .000 | .00000940 | .00003148 |
|  |  | 4 | -.000014590^*^ | .000006116 | .019 | -.00002673 | -.00000245 |
|  | 4 | 1 | .000057371^*^ | .000005969 | .000 | .00004553 | .00006922 |
|  |  | 2 | .000035033^*^ | .000005926 | .000 | .00002327 | .00004679 |
|  |  | 3 | .000014590^*^ | .000006116 | .019 | .00000245 | .00002673 |
| IFOF-L | 1 | 2 | -.000029422^*^ | .000005593 | .000 | -.00004052 | -.00001832 |
|  |  | 3 | -.000070290^*^ | .000005809 | .000 | -.00008182 | -.00005876 |
|  |  | 4 | -.000082100^*^ | .000006181 | .000 | -.00009437 | -.00006983 |
|  | 2 | 1 | .000029422^*^ | .000005593 | .000 | .00001832 | .00004052 |
|  |  | 3 | -.000040868^*^ | .000005761 | .000 | -.00005230 | -.00002943 |
|  |  | 4 | -.000052678^*^ | .000006136 | .000 | -.00006485 | -.00004050 |
|  | 3 | 1 | .000070290^*^ | .000005809 | .000 | .00005876 | .00008182 |
|  |  | 2 | .000040868^*^ | .000005761 | .000 | .00002943 | .00005230 |
|  |  | 4 | -.000011810 | .000006333 | .065 | -.00002438 | .00000076 |
|  | 4 | 1 | .000082100^*^ | .000006181 | .000 | .00006983 | .00009437 |
|  |  | 2 | .000052678^*^ | .000006136 | .000 | .00004050 | .00006485 |
|  |  | 3 | .000011810 | .000006333 | .065 | -.00000076 | .00002438 |
| IFOF-R | 1 | 2 | -.000044371^*^ | .000005085 | .000 | -.00005446 | -.00003428 |
|  |  | 3 | -.000048670^*^ | .000005281 | .000 | -.00005915 | -.00003819 |
|  |  | 4 | -.000066000^*^ | .000005619 | .000 | -.00007715 | -.00005485 |
|  | 2 | 1 | .000044371^*^ | .000005085 | .000 | .00003428 | .00005446 |
|  |  | 3 | -.000004299 | .000005238 | .414 | -.00001469 | .00000610 |
|  |  | 4 | -.000021629^*^ | .000005579 | .000 | -.00003270 | -.00001056 |
|  | 3 | 1 | .000048670^*^ | .000005281 | .000 | .00003819 | .00005915 |
|  |  | 2 | .000004299 | .000005238 | .414 | -.00000610 | .00001469 |
|  |  | 4 | -.000017330^*^ | .000005758 | .023 | -.00002876 | -.00000590 |
|  | 4 | 1 | .000066000^*^ | .000005619 | .000 | .00005485 | .00007715 |
|  |  | 2 | .000021629^*^ | .000005579 | .000 | .00001056 | .00003270 |
|  |  | 3 | .000017330^*^ | .000005758 | .023 | .00000590 | .00002876 |
| *. 平均值差值的显著性水平为 0.05。 | | | | | | | |

| **ANOVA-MD值比较** | | | | | | |
| --- | --- | --- | --- | --- | --- | --- |
|  | | 平方和 | 自由度 | 均方 | F | 显著性 |
| SLF-L | 组间 | .000 | 3 | .000 | 139.333 | .000 |
|  | 组内 | .000 | 98 | .000 |  |  |
|  | 总计 | .000 | 101 |  |  |  |
| SLF-R | 组间 | .000 | 3 | .000 | 88.823 | .000 |
|  | 组内 | .000 | 98 | .000 |  |  |
|  | 总计 | .000 | 101 |  |  |  |
| ILF-L | 组间 | .000 | 3 | .000 | 4.734 | .414 |
|  | 组内 | .000 | 98 | .000 |  |  |
|  | 总计 | .000 | 101 |  |  |  |
| ILF-R | 组间 | .000 | 3 | .000 | 66.385 | .000 |
|  | 组内 | .000 | 98 | .000 |  |  |
|  | 总计 | .000 | 101 |  |  |  |

| **多重比较-MD值比较** | | | | | | | |
| --- | --- | --- | --- | --- | --- | --- | --- |
| LSD | | | | | | | |
| 因变量 | (I) group | (J) group | 平均值差值 (I-J) | 标准 错误 | 显著性 | 95% 置信区间 | |
|  |  |  |  |  |  | 下限 | 上限 |
| SLF-L | 1 | 2 | -.000061081^*^ | .000004149 | .000 | -.00006931 | -.00005285 |
|  |  | 3 | -.000076557^*^ | .000004309 | .000 | -.00008511 | -.00006801 |
|  |  | 4 | -.000073807^*^ | .000004584 | .000 | -.00008290 | -.00006471 |
|  | 2 | 1 | .000061081^*^ | .000004149 | .000 | .00005285 | .00006931 |
|  |  | 3 | -.000015476 | .000004274 | .088 | -.00002396 | -.00000700 |
|  |  | 4 | -.000012726 | .000004551 | .106 | -.00002176 | -.00000369 |
|  | 3 | 1 | .000076557^*^ | .000004309 | .000 | .00006801 | .00008511 |
|  |  | 2 | .000015476 | .000004274 | .088 | .00000700 | .00002396 |
|  |  | 4 | .000002750 | .000004698 | .560 | -.00000657 | .00001207 |
|  | 4 | 1 | .000073807^*^ | .000004584 | .000 | .00006471 | .00008290 |
|  |  | 2 | .000012726 | .000004551 | .106 | .00000369 | .00002176 |
|  |  | 3 | -.000002750 | .000004698 | .560 | -.00001207 | .00000657 |
| SLF-R | 1 | 2 | -.000046443^*^ | .000005550 | .000 | -.00005746 | -.00003543 |
|  |  | 3 | -.000054257^*^ | .000005764 | .000 | -.00006570 | -.00004282 |
|  |  | 4 | -.000098907^*^ | .000006133 | .000 | -.00011108 | -.00008674 |
|  | 2 | 1 | .000046443^*^ | .000005550 | .000 | .00003543 | .00005746 |
|  |  | 3 | -.000007814 | .000005717 | .175 | -.00001916 | .00000353 |
|  |  | 4 | -.000052464^*^ | .000006089 | .003 | -.00006455 | -.00004038 |
|  | 3 | 1 | .000054257^*^ | .000005764 | .000 | .00004282 | .00006570 |
|  |  | 2 | .000007814 | .000005717 | .175 | -.00000353 | .00001916 |
|  |  | 4 | -.000044650^*^ | .000006285 | .027 | -.00005712 | -.00003218 |
|  | 4 | 1 | .000098907^*^ | .000006133 | .000 | .00008674 | .00011108 |
|  |  | 2 | .000052464^*^ | .000006089 | .003 | .00004038 | .00006455 |
|  |  | 3 | .000044650^*^ | .000006285 | .027 | .00003218 | .00005712 |
| ILF-L | 1 | 2 | -.000011793 | .000005095 | .073 | -.00002190 | -.00000168 |
|  |  | 3 | .000000360 | .000005292 | .946 | -.00001014 | .00001086 |
|  |  | 4 | -.000016700 | .000005630 | .224 | -.00002787 | -.00000553 |
|  | 2 | 1 | .000011793 | .000005095 | .073 | .00000168 | .00002190 |
|  |  | 3 | .000012153 | .000005249 | .103 | .00000174 | .00002257 |
|  |  | 4 | -.000004907 | .000005590 | .382 | -.00001600 | .00000619 |
|  | 3 | 1 | -.000000360 | .000005292 | .946 | -.00001086 | .00001014 |
|  |  | 2 | -.000012153 | .000005249 | .103 | -.00002257 | -.00000174 |
|  |  | 4 | -.000017060 | .000005769 | .094 | -.00002851 | -.00000561 |
|  | 4 | 1 | .000016700 | .000005630 | .224 | .00000553 | .00002787 |
|  |  | 2 | .000004907 | .000005590 | .382 | -.00000619 | .00001600 |
|  |  | 3 | .000017060 | .000005769 | .094 | .00000561 | .00002851 |
| ILF-R | 1 | 2 | -.000034031^*^ | .000004755 | .000 | -.00004347 | -.00002459 |
|  |  | 3 | -.000057653^*^ | .000004939 | .000 | -.00006745 | -.00004785 |
|  |  | 4 | -.000064333^*^ | .000005255 | .000 | -.00007476 | -.00005390 |
|  | 2 | 1 | .000034031^*^ | .000004755 | .000 | .00002459 | .00004347 |
|  |  | 3 | -.000023622^*^ | .000004898 | .000 | -.00003334 | -.00001390 |
|  |  | 4 | -.000030302^*^ | .000005217 | .000 | -.00004065 | -.00001995 |
|  | 3 | 1 | .000057653^*^ | .000004939 | .000 | .00004785 | .00006745 |
|  |  | 2 | .000023622^*^ | .000004898 | .000 | .00001390 | .00003334 |
|  |  | 4 | -.000006680 | .000005385 | .218 | -.00001737 | .00000401 |
|  | 4 | 1 | .000064333^*^ | .000005255 | .000 | .00005390 | .00007476 |
|  |  | 2 | .000030302^*^ | .000005217 | .000 | .00001995 | .00004065 |
|  |  | 3 | .000006680 | .000005385 | .218 | -.00000401 | .00001737 |
| *. 平均值差值的显著性水平为 0.05。 | | | | | | | |

| **ANOVA-MD值比较** | | | | | | |
| --- | --- | --- | --- | --- | --- | --- |
|  | | 平方和 | 自由度 | 均方 | F | 显著性 |
| UF-L | 组间 | .000 | 3 | .000 | 10.277 | .330 |
|  | 组内 | .000 | 98 | .000 |  |  |
|  | 总计 | .000 | 101 |  |  |  |
| UF-R | 组间 | .000 | 3 | .000 | 18.220 | .147 |
|  | 组内 | .000 | 98 | .000 |  |  |
|  | 总计 | .000 | 101 |  |  |  |
| tSLF-L | 组间 | .000 | 3 | .000 | 41.138 | .000 |
|  | 组内 | .000 | 98 | .000 |  |  |
|  | 总计 | .000 | 101 |  |  |  |
| tSLF-R | 组间 | .000 | 3 | .000 | 21.577 | .010 |
|  | 组内 | .000 | 98 | .000 |  |  |
|  | 总计 | .000 | 101 |  |  |  |

| **多重比较-MD值比较** | | | | | | | |
| --- | --- | --- | --- | --- | --- | --- | --- |
| LSD | | | | | | | |
| 因变量 | (I) group | (J) group | 平均值差值 (I-J) | 标准 错误 | 显著性 | 95% 置信区间 | |
|  |  |  |  |  |  | 下限 | 上限 |
| UF-L | 1 | 2 | -.000024644 | .000004780 | .233 | -.00003413 | -.00001516 |
|  |  | 3 | -.000019479 | .000004964 | .173 | -.00002933 | -.00000963 |
|  |  | 4 | -.000020479 | .000005282 | .202 | -.00003096 | -.00001000 |
|  | 2 | 1 | .000024644 | .000004780 | .233 | .00001516 | .00003413 |
|  |  | 3 | .000005166 | .000004923 | .297 | -.00000460 | .00001494 |
|  |  | 4 | .000004166 | .000005244 | .429 | -.00000624 | .00001457 |
|  | 3 | 1 | .000019479 | .000004964 | .173 | .00000963 | .00002933 |
|  |  | 2 | -.000005166 | .000004923 | .297 | -.00001494 | .00000460 |
|  |  | 4 | -.000001000 | .000005412 | .854 | -.00001174 | .00000974 |
|  | 4 | 1 | .000020479 | .000005282 | .202 | .00001000 | .00003096 |
|  |  | 2 | -.000004166 | .000005244 | .429 | -.00001457 | .00000624 |
|  |  | 3 | .000001000 | .000005412 | .854 | -.00000974 | .00001174 |
| UF-R | 1 | 2 | -.000026901 | .000004264 | .199 | -.00003536 | -.00001844 |
|  |  | 3 | -.000024063 | .000004429 | .237 | -.00003285 | -.00001527 |
|  |  | 4 | -.000027943 | .000004712 | .441 | -.00003729 | -.00001859 |
|  | 2 | 1 | .000026901 | .000004264 | .199 | .00001844 | .00003536 |
|  |  | 3 | .000002839 | .000004392 | .520 | -.00000588 | .00001156 |
|  |  | 4 | -.000001041 | .000004678 | .824 | -.00001032 | .00000824 |
|  | 3 | 1 | .000024063 | .000004429 | .237 | .00001527 | .00003285 |
|  |  | 2 | -.000002839 | .000004392 | .520 | -.00001156 | .00000588 |
|  |  | 4 | -.000003880 | .000004828 | .424 | -.00001346 | .00000570 |
|  | 4 | 1 | .000027943 | .000004712 | .441 | .00001859 | .00003729 |
|  |  | 2 | .000001041 | .000004678 | .824 | -.00000824 | .00001032 |
|  |  | 3 | .000003880 | .000004828 | .424 | -.00000570 | .00001346 |
| tSLF-L | 1 | 2 | -.000031711^*^ | .000004129 | .007 | -.00003990 | -.00002352 |
|  |  | 3 | -.000032407^*^ | .000004288 | .000 | -.00004092 | -.00002390 |
|  |  | 4 | -.000047307^*^ | .000004563 | .000 | -.00005636 | -.00003825 |
|  | 2 | 1 | .000031711^*^ | .000004129 | .007 | .00002352 | .00003990 |
|  |  | 3 | -.000000697 | .000004253 | .870 | -.00000914 | .00000774 |
|  |  | 4 | -.000015597^*^ | .000004530 | .010 | -.00002459 | -.00000661 |
|  | 3 | 1 | .000032407^*^ | .000004288 | .000 | .00002390 | .00004092 |
|  |  | 2 | .000000697 | .000004253 | .870 | -.00000774 | .00000914 |
|  |  | 4 | -.000014900^*^ | .000004675 | .032 | -.00002418 | -.00000562 |
|  | 4 | 1 | .000047307^*^ | .000004563 | .000 | .00003825 | .00005636 |
|  |  | 2 | .000015597^*^ | .000004530 | .010 | .00000661 | .00002459 |
|  |  | 3 | .000014900^*^ | .000004675 | .032 | .00000562 | .00002418 |
| tSLF-R | 1 | 2 | -.000033192^*^ | .000005283 | .000 | -.00004368 | -.00002271 |
|  |  | 3 | -.000035411^*^ | .000005487 | .000 | -.00004630 | -.00002452 |
|  |  | 4 | -.000038471^*^ | .000005838 | .000 | -.00005006 | -.00002689 |
|  | 2 | 1 | .000033192^*^ | .000005283 | .000 | .00002271 | .00004368 |
|  |  | 3 | -.000002219 | .000005442 | .684 | -.00001302 | .00000858 |
|  |  | 4 | -.000005279 | .000005796 | .365 | -.00001678 | .00000622 |
|  | 3 | 1 | .000035411^*^ | .000005487 | .000 | .00002452 | .00004630 |
|  |  | 2 | .000002219 | .000005442 | .684 | -.00000858 | .00001302 |
|  |  | 4 | -.000003060 | .000005982 | .610 | -.00001493 | .00000881 |
|  | 4 | 1 | .000038471^*^ | .000005838 | .000 | .00002689 | .00005006 |
|  |  | 2 | .000005279 | .000005796 | .365 | -.00000622 | .00001678 |
|  |  | 3 | .000003060 | .000005982 | .610 | -.00000881 | .00001493 |
| *. 平均值差值的显著性水平为 0.05。 | | | | | | | |

| **描述** | | | | | | | | | |
| --- | --- | --- | --- | --- | --- | --- | --- | --- | --- |
|  | | 个案数 | 平均值 | 标准 偏差 | 标准 错误 | 平均值的 95% 置信区间 | | 最小值 | 最大值 |
|  |  |  |  |  |  | 下限 | 上限 |  |  |
| 左侧额前回 | HC | 28 | 9.13804 | .646839 | .122241 | 8.88722 | 9.38885 | 7.884 | 10.337 |
|  | AD-nonWMH | 29 | 9.03590 | .875839 | .162639 | 8.70275 | 9.36905 | 7.442 | 11.232 |
|  | AD-miWMH | 25 | 8.95168 | .892132 | .178426 | 8.58343 | 9.31993 | 6.773 | 10.447 |
|  | AD-moWMH | 20 | 8.47520 | .991782 | .221769 | 8.01103 | 8.93937 | 6.294 | 9.901 |
|  | 总计 | 102 | 8.93335 | .868882 | .086032 | 8.76269 | 9.10402 | 6.294 | 11.232 |
| 右侧额前回 | HC | 28 | 9.27111 | .643607 | .121630 | 9.02154 | 9.52067 | 8.023 | 11.434 |
|  | AD-nonWMH | 29 | 9.14945 | .546876 | .101552 | 8.94143 | 9.35747 | 8.033 | 10.284 |
|  | AD-miWMH | 25 | 9.13080 | .418826 | .083765 | 8.95792 | 9.30368 | 8.273 | 9.744 |
|  | AD-moWMH | 20 | 9.03515 | .600272 | .134225 | 8.75421 | 9.31609 | 8.034 | 10.244 |
|  | 总计 | 102 | 9.15586 | .556640 | .055116 | 9.04653 | 9.26520 | 8.023 | 11.434 |
| 左侧额上回 | HC | 28 | 9.57268 | .352570 | .066630 | 9.43597 | 9.70939 | 8.947 | 10.282 |
|  | AD-nonWMH | 29 | 9.08900 | .323893 | .060145 | 8.96580 | 9.21220 | 8.277 | 9.577 |
|  | AD-miWMH | 25 | 8.97192 | .672363 | .134473 | 8.69438 | 9.24946 | 7.580 | 10.233 |
|  | AD-moWMH | 20 | 8.91310 | .614960 | .137509 | 8.62529 | 9.20091 | 7.092 | 9.997 |
|  | 总计 | 102 | 9.15859 | .557103 | .055161 | 9.04916 | 9.26801 | 7.092 | 10.282 |
| 右侧额上回 | HC | 28 | 9.64111 | 1.289432 | .243680 | 9.14112 | 10.14110 | 7.366 | 12.377 |
|  | AD-nonWMH | 29 | 10.26383 | 1.175696 | .218321 | 9.81662 | 10.71104 | 8.288 | 12.395 |
|  | AD-miWMH | 25 | 10.80500 | 1.300410 | .260082 | 10.26822 | 11.34178 | 8.524 | 13.461 |
|  | AD-moWMH | 20 | 8.29160 | .931226 | .208228 | 7.85577 | 8.72743 | 6.377 | 10.407 |
|  | 总计 | 102 | 9.83881 | 1.469844 | .145536 | 9.55011 | 10.12752 | 6.377 | 13.461 |
| 左侧额中回 | HC | 28 | 7.23961 | 1.489273 | .281446 | 6.66213 | 7.81709 | 3.355 | 9.483 |
|  | AD-nonWMH | 29 | 3.07497 | 1.000821 | .185848 | 2.69427 | 3.45566 | .977 | 4.480 |
|  | AD-miWMH | 25 | .48628 | .031162 | .006232 | .47342 | .49914 | .433 | .522 |
|  | AD-moWMH | 20 | .42040 | .013212 | .002954 | .41422 | .42658 | .399 | .443 |
|  | 总计 | 102 | 3.06322 | 2.955190 | .292607 | 2.48276 | 3.64367 | .399 | 9.483 |
| 右侧额中回 | HC | 28 | 6.06879 | .556921 | .105248 | 5.85283 | 6.28474 | 4.530 | 7.420 |
|  | AD-nonWMH | 29 | 4.72841 | 1.222338 | .226983 | 4.26346 | 5.19337 | 1.773 | 6.664 |
|  | AD-miWMH | 25 | 1.18212 | .133713 | .026743 | 1.12693 | 1.23731 | .889 | 1.423 |
|  | AD-moWMH | 20 | .56775 | .097883 | .021887 | .52194 | .61356 | .447 | .848 |
|  | 总计 | 102 | 3.41135 | 2.406301 | .238259 | 2.93871 | 3.88400 | .447 | 7.420 |
| 左侧额下回 | HC | 28 | 6.77618 | .762431 | .144086 | 6.48054 | 7.07182 | 5.478 | 8.502 |
|  | AD-nonWMH | 29 | 7.06383 | .861775 | .160028 | 6.73603 | 7.39163 | 5.452 | 8.480 |
|  | AD-miWMH | 25 | 7.11384 | .899816 | .179963 | 6.74241 | 7.48527 | 5.477 | 8.483 |
|  | AD-moWMH | 20 | 6.79090 | 1.099973 | .245961 | 6.27610 | 7.30570 | 4.401 | 8.460 |
|  | 总计 | 102 | 6.94361 | .897220 | .088838 | 6.76738 | 7.11984 | 4.401 | 8.502 |
| 右侧额下回 | HC | 28 | 7.46750 | 1.202492 | .227250 | 7.00122 | 7.93378 | 5.076 | 9.855 |
|  | AD-nonWMH | 29 | 8.37897 | .906555 | .168343 | 8.03413 | 8.72380 | 6.094 | 9.649 |
|  | AD-miWMH | 25 | 8.52384 | 1.117109 | .223422 | 8.06272 | 8.98496 | 5.463 | 9.472 |
|  | AD-moWMH | 20 | 8.00085 | .950261 | .212485 | 7.55611 | 8.44559 | 6.340 | 9.442 |
|  | 总计 | 102 | 8.09013 | 1.122659 | .111160 | 7.86962 | 8.31064 | 5.076 | 9.855 |

| **方差齐性检验** | | | | | |
| --- | --- | --- | --- | --- | --- |
|  | | 莱文统计 | 自由度 1 | 自由度 2 | 显著性 |
| 左侧额前回 | 基于平均值 | 1.022 | 3 | 98 | .387 |
|  | 基于中位数 | .659 | 3 | 98 | .579 |
|  | 基于中位数并具有调整后自由度 | .659 | 3 | 85.106 | .580 |
|  | 基于剪除后平均值 | .956 | 3 | 98 | .417 |
| 右侧额前回 | 基于平均值 | .541 | 3 | 98 | .655 |
|  | 基于中位数 | .463 | 3 | 98 | .709 |
|  | 基于中位数并具有调整后自由度 | .463 | 3 | 83.591 | .709 |
|  | 基于剪除后平均值 | .555 | 3 | 98 | .646 |
| 左侧额上回 | 基于平均值 | 4.191 | 3 | 98 | .088 |
|  | 基于中位数 | 3.787 | 3 | 98 | .093 |
|  | 基于中位数并具有调整后自由度 | 3.787 | 3 | 66.988 | .094 |
|  | 基于剪除后平均值 | 4.068 | 3 | 98 | .089 |
| 右侧额上回 | 基于平均值 | 1.481 | 3 | 98 | .224 |
|  | 基于中位数 | .940 | 3 | 98 | .424 |
|  | 基于中位数并具有调整后自由度 | .940 | 3 | 93.078 | .425 |
|  | 基于剪除后平均值 | 1.391 | 3 | 98 | .250 |
| 左侧额中回 | 基于平均值 | 21.319 | 3 | 98 | .000 |
|  | 基于中位数 | 15.961 | 3 | 98 | .000 |
|  | 基于中位数并具有调整后自由度 | 15.961 | 3 | 41.919 | .000 |
|  | 基于剪除后平均值 | 19.360 | 3 | 98 | .000 |
| 右侧额中回 | 基于平均值 | 28.163 | 3 | 98 | .000 |
|  | 基于中位数 | 24.154 | 3 | 98 | .000 |
|  | 基于中位数并具有调整后自由度 | 24.154 | 3 | 41.781 | .000 |
|  | 基于剪除后平均值 | 28.265 | 3 | 98 | .000 |
| 左侧额下回 | 基于平均值 | 1.303 | 3 | 98 | .278 |
|  | 基于中位数 | 1.141 | 3 | 98 | .337 |
|  | 基于中位数并具有调整后自由度 | 1.141 | 3 | 96.334 | .337 |
|  | 基于剪除后平均值 | 1.304 | 3 | 98 | .278 |
| 右侧额下回 | 基于平均值 | .799 | 3 | 98 | .498 |
|  | 基于中位数 | .851 | 3 | 98 | .469 |
|  | 基于中位数并具有调整后自由度 | .851 | 3 | 90.905 | .470 |
|  | 基于剪除后平均值 | .917 | 3 | 98 | .436 |

| **ANOVA** | | | | | | |
| --- | --- | --- | --- | --- | --- | --- |
|  | | 平方和 | 自由度 | 均方 | F | 显著性 |
| 左侧额前回 | 组间 | 5.684 | 3 | 1.895 | 2.631 | .084 |
|  | 组内 | 70.566 | 98 | .720 |  |  |
|  | 总计 | 76.250 | 101 |  |  |  |
| 右侧额前回 | 组间 | .680 | 3 | .227 | .726 | .539 |
|  | 组内 | 30.614 | 98 | .312 |  |  |
|  | 总计 | 31.295 | 101 |  |  |  |
| 左侧额上回 | 组间 | 7.018 | 3 | 2.339 | 9.423 | .048 |
|  | 组内 | 24.329 | 98 | .248 |  |  |
|  | 总计 | 31.347 | 101 |  |  |  |
| 右侧额上回 | 组间 | 77.548 | 3 | 25.849 | 18.010 | .182 |
|  | 组内 | 140.656 | 98 | 1.435 |  |  |
|  | 总计 | 218.205 | 101 |  |  |  |
| 左侧额中回 | 组间 | 794.091 | 3 | 264.697 | 294.921 | .000 |
|  | 组内 | 87.957 | 98 | .898 |  |  |
|  | 总计 | 882.048 | 101 |  |  |  |
| 右侧额中回 | 组间 | 533.998 | 3 | 177.999 | 343.245 | .000 |
|  | 组内 | 50.821 | 98 | .519 |  |  |
|  | 总计 | 584.819 | 101 |  |  |  |
| 左侧额下回 | 组间 | 2.395 | 3 | .798 | .991 | .400 |
|  | 组内 | 78.910 | 98 | .805 |  |  |
|  | 总计 | 81.305 | 101 |  |  |  |
| 右侧额下回 | 组间 | 18.136 | 3 | 6.045 | 5.427 | .042 |
|  | 组内 | 109.160 | 98 | 1.114 |  |  |
|  | 总计 | 127.297 | 101 |  |  |  |

| **多重比较** | | | | | | | | |
| --- | --- | --- | --- | --- | --- | --- | --- | --- |
| 因变量 | | (I) group | (J) group | 平均值差值 (I-J) | 标准 错误 | 显著性 | 95% 置信区间 | |
|  |  |  |  |  |  |  | 下限 | 上限 |
| 左侧额前回 | LSD | HC | AD-nonWMH | .102139 | .224825 | .651 | -.34402 | .54830 |
|  |  |  | AD-miWMH | .186356 | .233493 | .427 | -.27700 | .64971 |
|  |  |  | AD-moWMH | .662836 | .248434 | .079 | .16983 | 1.15585 |
|  |  | AD-nonWMH | HC | -.102139 | .224825 | .651 | -.54830 | .34402 |
|  |  |  | AD-miWMH | .084217 | .231586 | .717 | -.37536 | .54379 |
|  |  |  | AD-moWMH | .560697 | .246643 | .125 | .07124 | 1.05015 |
|  |  | AD-miWMH | HC | -.186356 | .233493 | .427 | -.64971 | .27700 |
|  |  |  | AD-nonWMH | -.084217 | .231586 | .717 | -.54379 | .37536 |
|  |  |  | AD-moWMH | .476480 | .254569 | .064 | -.02870 | .98166 |
|  |  | AD-moWMH | HC | -.662836 | .248434 | .079 | -1.15585 | -.16983 |
|  |  |  | AD-nonWMH | -.560697 | .246643 | .125 | -1.05015 | -.07124 |
|  |  |  | AD-miWMH | -.476480 | .254569 | .064 | -.98166 | .02870 |
|  | 塔姆黑尼 | HC | AD-nonWMH | .102139 | .203456 | .997 | -.45450 | .65878 |
|  |  |  | AD-miWMH | .186356 | .216284 | .950 | -.40986 | .78257 |
|  |  |  | AD-moWMH | .662836 | .253228 | .079 | -.04972 | 1.37539 |
|  |  | AD-nonWMH | HC | -.102139 | .203456 | .997 | -.65878 | .45450 |
|  |  |  | AD-miWMH | .084217 | .241428 | 1.000 | -.57682 | .74526 |
|  |  |  | AD-moWMH | .560697 | .275015 | .258 | -.20299 | 1.32439 |
|  |  | AD-miWMH | HC | -.186356 | .216284 | .950 | -.78257 | .40986 |
|  |  |  | AD-nonWMH | -.084217 | .241428 | 1.000 | -.74526 | .57682 |
|  |  |  | AD-moWMH | .476480 | .284636 | .476 | -.31263 | 1.26559 |
|  |  | AD-moWMH | HC | -.662836 | .253228 | .079 | -1.37539 | .04972 |
|  |  |  | AD-nonWMH | -.560697 | .275015 | .258 | -1.32439 | .20299 |
|  |  |  | AD-miWMH | -.476480 | .284636 | .476 | -1.26559 | .31263 |
| 右侧额前回 | LSD | HC | AD-nonWMH | .121659 | .148084 | .413 | -.17221 | .41553 |
|  |  |  | AD-miWMH | .140307 | .153794 | .364 | -.16489 | .44551 |
|  |  |  | AD-moWMH | .235957 | .163635 | .152 | -.08877 | .56069 |
|  |  | AD-nonWMH | HC | -.121659 | .148084 | .413 | -.41553 | .17221 |
|  |  |  | AD-miWMH | .018648 | .152538 | .903 | -.28406 | .32135 |
|  |  |  | AD-moWMH | .114298 | .162455 | .483 | -.20809 | .43669 |
|  |  | AD-miWMH | HC | -.140307 | .153794 | .364 | -.44551 | .16489 |
|  |  |  | AD-nonWMH | -.018648 | .152538 | .903 | -.32135 | .28406 |
|  |  |  | AD-moWMH | .095650 | .167676 | .570 | -.23710 | .42840 |
|  |  | AD-moWMH | HC | -.235957 | .163635 | .152 | -.56069 | .08877 |
|  |  |  | AD-nonWMH | -.114298 | .162455 | .483 | -.43669 | .20809 |
|  |  |  | AD-miWMH | -.095650 | .167676 | .570 | -.42840 | .23710 |
|  | 塔姆黑尼 | HC | AD-nonWMH | .121659 | .158451 | .971 | -.31139 | .55471 |
|  |  |  | AD-miWMH | .140307 | .147684 | .922 | -.26536 | .54597 |
|  |  |  | AD-moWMH | .235957 | .181136 | .737 | -.26370 | .73561 |
|  |  | AD-nonWMH | HC | -.121659 | .158451 | .971 | -.55471 | .31139 |
|  |  |  | AD-miWMH | .018648 | .131641 | 1.000 | -.34157 | .37886 |
|  |  |  | AD-moWMH | .114298 | .168313 | .985 | -.35251 | .58110 |
|  |  | AD-miWMH | HC | -.140307 | .147684 | .922 | -.54597 | .26536 |
|  |  |  | AD-nonWMH | -.018648 | .131641 | 1.000 | -.37886 | .34157 |
|  |  |  | AD-moWMH | .095650 | .158218 | .992 | -.34730 | .53860 |
|  |  | AD-moWMH | HC | -.235957 | .181136 | .737 | -.73561 | .26370 |
|  |  |  | AD-nonWMH | -.114298 | .168313 | .985 | -.58110 | .35251 |
|  |  |  | AD-miWMH | -.095650 | .158218 | .992 | -.53860 | .34730 |
| 左侧额上回 | LSD | HC | AD-nonWMH | .483679 | .132010 | .170 | .22171 | .74565 |
|  |  |  | AD-miWMH | .600759 | .137099 | .280 | .32869 | .87283 |
|  |  |  | AD-moWMH | .659579 | .145872 | .140 | .37010 | .94906 |
|  |  | AD-nonWMH | HC | -.483679 | .132010 | .170 | -.74565 | -.22171 |
|  |  |  | AD-miWMH | .117080^*^ | .135980 | .019 | -.15277 | .38693 |
|  |  |  | AD-moWMH | .175900 | .144821 | .227 | -.11149 | .46329 |
|  |  | AD-miWMH | HC | -.600759 | .137099 | .280 | -.87283 | -.32869 |
|  |  |  | AD-nonWMH | -.117080^*^ | .135980 | .019 | -.38693 | .15277 |
|  |  |  | AD-moWMH | .058820 | .149475 | .695 | -.23781 | .35545 |
|  |  | AD-moWMH | HC | -.659579 | .145872 | .140 | -.94906 | -.37010 |
|  |  |  | AD-nonWMH | -.175900 | .144821 | .227 | -.46329 | .11149 |
|  |  |  | AD-miWMH | -.058820 | .149475 | .695 | -.35545 | .23781 |
|  | 塔姆黑尼 | HC | AD-nonWMH | .483679 | .089761 | .070 | .23858 | .72877 |
|  |  |  | AD-miWMH | .600759 | .150075 | .092 | .18256 | 1.01895 |
|  |  |  | AD-moWMH | .659579 | .152801 | .301 | .22700 | 1.09216 |
|  |  | AD-nonWMH | HC | -.483679 | .089761 | .070 | -.72877 | -.23858 |
|  |  |  | AD-miWMH | .117080^*^ | .147310 | .027 | -.29480 | .52896 |
|  |  |  | AD-moWMH | .175900 | .150087 | .824 | -.25093 | .60273 |
|  |  | AD-miWMH | HC | -.600759 | .150075 | .092 | -1.01895 | -.18256 |
|  |  |  | AD-nonWMH | -.117080^*^ | .147310 | .027 | -.52896 | .29480 |
|  |  |  | AD-moWMH | .058820 | .192332 | 1.000 | -.47206 | .58970 |
|  |  | AD-moWMH | HC | -.659579 | .152801 | .301 | -1.09216 | -.22700 |
|  |  |  | AD-nonWMH | -.175900 | .150087 | .824 | -.60273 | .25093 |
|  |  |  | AD-miWMH | -.058820 | .192332 | 1.000 | -.58970 | .47206 |
| 右侧额上回 | LSD | HC | AD-nonWMH | -.622720 | .317414 | .053 | -1.25262 | .00718 |
|  |  |  | AD-miWMH | -1.163893 | .329652 | .071 | -1.81808 | -.50971 |
|  |  |  | AD-moWMH | 1.349507 | .350747 | .088 | .65346 | 2.04555 |
|  |  | AD-nonWMH | HC | .622720 | .317414 | .053 | -.00718 | 1.25262 |
|  |  |  | AD-miWMH | -.541172 | .326960 | .101 | -1.19001 | .10767 |
|  |  |  | AD-moWMH | 1.972228 | .348218 | .071 | 1.28120 | 2.66325 |
|  |  | AD-miWMH | HC | 1.163893 | .329652 | .071 | .50971 | 1.81808 |
|  |  |  | AD-nonWMH | .541172 | .326960 | .101 | -.10767 | 1.19001 |
|  |  |  | AD-moWMH | 2.513400 | .359408 | .068 | 1.80017 | 3.22663 |
|  |  | AD-moWMH | HC | -1.349507 | .350747 | .088 | -2.04555 | -.65346 |
|  |  |  | AD-nonWMH | -1.972228 | .348218 | .710 | -2.66325 | -1.28120 |
|  |  |  | AD-miWMH | -2.513400 | .359408 | .068 | -3.22663 | -1.80017 |
|  | 塔姆黑尼 | HC | AD-nonWMH | -.622720 | .327176 | .320 | -1.51615 | .27071 |
|  |  |  | AD-miWMH | -1.163893 | .356402 | .072 | -2.13998 | -.18780 |
|  |  |  | AD-moWMH | 1.349507 | .320529 | .101 | .46835 | 2.23067 |
|  |  | AD-nonWMH | HC | .622720 | .327176 | .320 | -.27071 | 1.51615 |
|  |  |  | AD-miWMH | -.541172 | .339569 | .527 | -1.47218 | .38983 |
|  |  |  | AD-moWMH | 1.972228 | .301701 | .080 | 1.14285 | 2.80161 |
|  |  | AD-miWMH | HC | 1.163893 | .356402 | .072 | .18780 | 2.13998 |
|  |  |  | AD-nonWMH | .541172 | .339569 | .527 | -.38983 | 1.47218 |
|  |  |  | AD-moWMH | 2.513400 | .333169 | .092 | 1.59417 | 3.43263 |
|  |  | AD-moWMH | HC | -1.349507 | .320529 | .101 | -2.23067 | -.46835 |
|  |  |  | AD-nonWMH | -1.972228 | .301701 | .080 | -2.80161 | -1.14285 |
|  |  |  | AD-miWMH | -2.513400 | .333169 | .092 | -3.43263 | -1.59417 |
| 左侧额中回 | LSD | HC | AD-nonWMH | 4.164642^*^ | .251004 | .000 | 3.66653 | 4.66275 |
|  |  |  | AD-miWMH | 6.753327^*^ | .260682 | .000 | 6.23601 | 7.27064 |
|  |  |  | AD-moWMH | 6.819207^*^ | .277363 | .000 | 6.26879 | 7.36962 |
|  |  | AD-nonWMH | HC | -4.164642^*^ | .251004 | .000 | -4.66275 | -3.66653 |
|  |  |  | AD-miWMH | 2.588686^*^ | .258553 | .000 | 2.07560 | 3.10178 |
|  |  |  | AD-moWMH | 2.654566^*^ | .275363 | .000 | 2.10812 | 3.20101 |
|  |  | AD-miWMH | HC | -6.753327^*^ | .260682 | .000 | -7.27064 | -6.23601 |
|  |  |  | AD-nonWMH | -2.588686^*^ | .258553 | .000 | -3.10178 | -2.07560 |
|  |  |  | AD-moWMH | -.065880^*^ | .284212 | .017 | -.49813 | .62989 |
|  |  | AD-moWMH | HC | -6.819207^*^ | .277363 | .000 | -7.36962 | -6.26879 |
|  |  |  | AD-nonWMH | -2.654566^*^ | .275363 | .000 | -3.20101 | -2.10812 |
|  |  |  | AD-miWMH | -.065880^*^ | .284212 | .017 | -.62989 | .49813 |
|  | 塔姆黑尼 | HC | AD-nonWMH | 4.164642^*^ | .337271 | .000 | 3.23840 | 5.09089 |
|  |  |  | AD-miWMH | 6.753327^*^ | .281515 | .000 | 5.95443 | 7.55222 |
|  |  |  | AD-moWMH | 6.819207^*^ | .281462 | .000 | 6.02042 | 7.61800 |
|  |  | AD-nonWMH | HC | -4.164642^*^ | .337271 | .000 | -5.09089 | -3.23840 |
|  |  |  | AD-miWMH | 2.588686^*^ | .185952 | .000 | 2.06250 | 3.11487 |
|  |  |  | AD-moWMH | 2.654566^*^ | .185871 | .000 | 2.12854 | 3.18059 |
|  |  | AD-miWMH | HC | -6.753327^*^ | .281515 | .000 | -7.55222 | -5.95443 |
|  |  |  | AD-nonWMH | -2.588686^*^ | .185952 | .000 | -3.11487 | -2.06250 |
|  |  |  | AD-moWMH | .065880^*^ | .006897 | .000 | .04661 | .08515 |
|  |  | AD-moWMH | HC | -6.819207^*^ | .281462 | .000 | -7.61800 | -6.02042 |
|  |  |  | AD-nonWMH | -2.654566^*^ | .185871 | .000 | -3.18059 | -2.12854 |
|  |  |  | AD-miWMH | -.065880^*^ | .006897 | .000 | -.08515 | -.04661 |
| 右侧额中回 | LSD | HC | AD-nonWMH | 1.340372 | .190795 | .087 | .96175 | 1.71900 |
|  |  |  | AD-miWMH | 4.886666^*^ | .198151 | .000 | 4.49344 | 5.27989 |
|  |  |  | AD-moWMH | 5.501036^*^ | .210830 | .000 | 5.08265 | 5.91942 |
|  |  | AD-nonWMH | HC | -1.340372 | .190795 | .087 | -1.71900 | -.96175 |
|  |  |  | AD-miWMH | 3.546294^*^ | .196533 | .000 | 3.15628 | 3.93631 |
|  |  |  | AD-moWMH | 4.160664^*^ | .209310 | .000 | 3.74529 | 4.57603 |
|  |  | AD-miWMH | HC | -4.886666^*^ | .198151 | .000 | -5.27989 | -4.49344 |
|  |  |  | AD-nonWMH | -3.546294^*^ | .196533 | .000 | -3.93631 | -3.15628 |
|  |  |  | AD-moWMH | .614370^*^ | .216037 | .005 | .18565 | 1.04309 |
|  |  | AD-moWMH | HC | -5.501036^*^ | .210830 | .000 | -5.91942 | -5.08265 |
|  |  |  | AD-nonWMH | -4.160664^*^ | .209310 | .000 | -4.57603 | -3.74529 |
|  |  |  | AD-miWMH | -.614370^*^ | .216037 | .005 | -1.04309 | -.18565 |
|  | 塔姆黑尼 | HC | AD-nonWMH | 1.340372 | .250196 | .090 | .64743 | 2.03332 |
|  |  |  | AD-miWMH | 4.886666^*^ | .108593 | .000 | 4.58119 | 5.19215 |
|  |  |  | AD-moWMH | 5.501036^*^ | .107500 | .000 | 5.19781 | 5.80426 |
|  |  | AD-nonWMH | HC | -1.340372 | .250196 | .090 | -2.03332 | -.64743 |
|  |  |  | AD-miWMH | 3.546294^*^ | .228552 | .000 | 2.90077 | 4.19182 |
|  |  |  | AD-moWMH | 4.160664^*^ | .228035 | .000 | 3.51617 | 4.80515 |
|  |  | AD-miWMH | HC | -4.886666^*^ | .108593 | .000 | -5.19215 | -4.58119 |
|  |  |  | AD-nonWMH | -3.546294^*^ | .228552 | .000 | -4.19182 | -2.90077 |
|  |  |  | AD-moWMH | .614370^*^ | .034558 | .000 | .51904 | .70970 |
|  |  | AD-moWMH | HC | -5.501036^*^ | .107500 | .000 | -5.80426 | -5.19781 |
|  |  |  | AD-nonWMH | -4.160664^*^ | .228035 | .000 | -4.80515 | -3.51617 |
|  |  |  | AD-miWMH | -.614370^*^ | .034558 | .000 | -.70970 | -.51904 |
| 左侧额下回 | LSD | HC | AD-nonWMH | -.287649 | .237746 | .229 | -.75945 | .18415 |
|  |  |  | AD-miWMH | -.337661 | .246912 | .175 | -.82765 | .15233 |
|  |  |  | AD-moWMH | -.014721 | .262713 | .955 | -.53607 | .50662 |
|  |  | AD-nonWMH | HC | .287649 | .237746 | .229 | -.18415 | .75945 |
|  |  |  | AD-miWMH | -.050012 | .244896 | .839 | -.53600 | .43598 |
|  |  |  | AD-moWMH | .272928 | .260818 | .298 | -.24466 | .79051 |
|  |  | AD-miWMH | HC | .337661 | .246912 | .175 | -.15233 | .82765 |
|  |  |  | AD-nonWMH | .050012 | .244896 | .839 | -.43598 | .53600 |
|  |  |  | AD-moWMH | .322940 | .269200 | .233 | -.21128 | .85716 |
|  |  | AD-moWMH | HC | .014721 | .262713 | .955 | -.50662 | .53607 |
|  |  |  | AD-nonWMH | -.272928 | .260818 | .298 | -.79051 | .24466 |
|  |  |  | AD-miWMH | -.322940 | .269200 | .233 | -.85716 | .21128 |
|  | 塔姆黑尼 | HC | AD-nonWMH | -.287649 | .215336 | .712 | -.87548 | .30018 |
|  |  |  | AD-miWMH | -.337661 | .230538 | .622 | -.97062 | .29530 |
|  |  |  | AD-moWMH | -.014721 | .285057 | 1.000 | -.81453 | .78508 |
|  |  | AD-nonWMH | HC | .287649 | .215336 | .712 | -.30018 | .87548 |
|  |  |  | AD-miWMH | -.050012 | .240823 | 1.000 | -.70963 | .60960 |
|  |  |  | AD-moWMH | .272928 | .293438 | .931 | -.54619 | 1.09205 |
|  |  | AD-miWMH | HC | .337661 | .230538 | .622 | -.29530 | .97062 |
|  |  |  | AD-nonWMH | .050012 | .240823 | 1.000 | -.60960 | .70963 |
|  |  |  | AD-moWMH | .322940 | .304768 | .879 | -.52472 | 1.17060 |
|  |  | AD-moWMH | HC | .014721 | .285057 | 1.000 | -.78508 | .81453 |
|  |  |  | AD-nonWMH | -.272928 | .293438 | .931 | -1.09205 | .54619 |
|  |  |  | AD-miWMH | -.322940 | .304768 | .879 | -1.17060 | .52472 |
| 右侧额下回 | LSD | HC | AD-nonWMH | -.911466 | .279627 | .072 | -1.46638 | -.35655 |
|  |  |  | AD-miWMH | -1.056340 | .290408 | .090 | -1.63265 | -.48003 |
|  |  |  | AD-moWMH | -.533350 | .308991 | .087 | -1.14653 | .07983 |
|  |  | AD-nonWMH | HC | .911466 | .279627 | .072 | .35655 | 1.46638 |
|  |  |  | AD-miWMH | -.144874 | .288037 | .616 | -.71647 | .42672 |
|  |  |  | AD-moWMH | .378116 | .306764 | .221 | -.23065 | .98688 |
|  |  | AD-miWMH | HC | 1.056340 | .290408 | .090 | .48003 | 1.63265 |
|  |  |  | AD-nonWMH | .144874 | .288037 | .616 | -.42672 | .71647 |
|  |  |  | AD-moWMH | .522990 | .316622 | .102 | -.10534 | 1.15132 |
|  |  | AD-moWMH | HC | .533350 | .308991 | .087 | -.07983 | 1.14653 |
|  |  |  | AD-nonWMH | -.378116 | .306764 | .221 | -.98688 | .23065 |
|  |  |  | AD-miWMH | -.522990 | .316622 | .102 | -1.15132 | .10534 |
|  | 塔姆黑尼 | HC | AD-nonWMH | -.911466 | .282810 | .113 | -1.68603 | -.13690 |
|  |  |  | AD-miWMH | -1.056340 | .318684 | .210 | -1.92865 | -.18403 |
|  |  |  | AD-moWMH | -.533350 | .311114 | .444 | -1.38907 | .32237 |
|  |  | AD-nonWMH | HC | .911466 | .282810 | .113 | .13690 | 1.68603 |
|  |  |  | AD-miWMH | -.144874 | .279744 | .996 | -.91374 | .62399 |
|  |  |  | AD-moWMH | .378116 | .271089 | .675 | -.37242 | 1.12865 |
|  |  | AD-miWMH | HC | 1.056340 | .318684 | .210 | .18403 | 1.92865 |
|  |  |  | AD-nonWMH | .144874 | .279744 | .996 | -.62399 | .91374 |
|  |  |  | AD-moWMH | .522990 | .308330 | .458 | -.32745 | 1.37343 |
|  |  | AD-moWMH | HC | .533350 | .311114 | .444 | -.32237 | 1.38907 |
|  |  |  | AD-nonWMH | -.378116 | .271089 | .675 | -1.12865 | .37242 |
|  |  |  | AD-miWMH | -.522990 | .308330 | .458 | -1.37343 | .32745 |
| *. 平均值差值的显著性水平为 0.05。 | | | | | | | | |

| **描述** | | | | | | | | | |
| --- | --- | --- | --- | --- | --- | --- | --- | --- | --- |
|  | | 个案数 | 平均值 | 标准 偏差 | 标准 错误 | 平均值的 95% 置信区间 | | 最小值 | 最大值 |
|  |  |  |  |  |  | 下限 | 上限 |  |  |
| 左侧角回 | HC | 28 | 9.59361 | 1.155064 | .218287 | 9.14572 | 10.04149 | 7.655 | 12.680 |
|  | AD-nonWMH | 29 | 9.11686 | .829096 | .153959 | 8.80149 | 9.43223 | 7.620 | 10.638 |
|  | AD-miWMH | 25 | 8.41920 | .929727 | .185945 | 8.03543 | 8.80297 | 6.495 | 9.680 |
|  | AD-moWMH | 20 | 6.32705 | 1.268882 | .283731 | 5.73320 | 6.92090 | 4.434 | 9.429 |
|  | 总计 | 102 | 8.52972 | 1.559500 | .154414 | 8.22340 | 8.83603 | 4.434 | 12.680 |
| 右侧角回 | HC | 28 | 9.04875 | 1.001227 | .189214 | 8.66051 | 9.43699 | 7.522 | 11.542 |
|  | AD-nonWMH | 29 | 8.24345 | .842610 | .156469 | 7.92294 | 8.56396 | 6.506 | 9.545 |
|  | AD-miWMH | 25 | 7.47596 | 1.148327 | .229665 | 7.00195 | 7.94997 | 4.592 | 9.632 |
|  | AD-moWMH | 20 | 5.47180 | .935936 | .209282 | 5.03377 | 5.90983 | 4.205 | 7.380 |
|  | 总计 | 102 | 7.73294 | 1.589505 | .157384 | 7.42073 | 8.04515 | 4.205 | 11.542 |
| 左侧楔前回 | HC | 28 | 6.13068 | .903479 | .170742 | 5.78035 | 6.48101 | 4.859 | 7.984 |
|  | AD-nonWMH | 29 | 4.97359 | .580334 | .107765 | 4.75284 | 5.19433 | 3.849 | 6.233 |
|  | AD-miWMH | 25 | 4.07752 | .511948 | .102390 | 3.86620 | 4.28884 | 3.424 | 4.521 |
|  | AD-moWMH | 20 | 2.96630 | .520645 | .116420 | 2.72263 | 3.20997 | 2.177 | 3.430 |
|  | 总计 | 102 | 4.67801 | 1.305526 | .129266 | 4.42158 | 4.93444 | 2.177 | 7.984 |
| 右侧楔前回 | HC | 28 | 6.57425 | .612397 | .115732 | 6.33679 | 6.81171 | 5.483 | 7.954 |
|  | AD-nonWMH | 29 | 5.38303 | .630199 | .117025 | 5.14332 | 5.62275 | 3.402 | 6.812 |
|  | AD-miWMH | 25 | 3.92212 | .719754 | .143951 | 3.62502 | 4.21922 | 2.452 | 5.489 |
|  | AD-moWMH | 20 | 2.91855 | .690207 | .154335 | 2.59552 | 3.24158 | 2.252 | 4.444 |
|  | 总计 | 102 | 4.86874 | 1.510076 | .149520 | 4.57213 | 5.16534 | 2.252 | 7.954 |
| 左侧顶上回 | HC | 28 | 1.29457 | .144339 | .027277 | 1.23860 | 1.35054 | .991 | 1.502 |
|  | AD-nonWMH | 29 | 1.11966 | .136325 | .025315 | 1.06780 | 1.17151 | .889 | 1.372 |
|  | AD-miWMH | 25 | .72236 | .110686 | .022137 | .67667 | .76805 | .443 | .920 |
|  | AD-moWMH | 20 | .51570 | .163646 | .036592 | .43911 | .59229 | .329 | .889 |
|  | 总计 | 102 | .95187 | .331697 | .032843 | .88672 | 1.01702 | .329 | 1.502 |
| 右侧顶上回 | HC | 28 | 1.29886 | .143650 | .027147 | 1.24316 | 1.35456 | 1.042 | 1.732 |
|  | AD-nonWMH | 29 | 1.09841 | .119534 | .022197 | 1.05295 | 1.14388 | .878 | 1.322 |
|  | AD-miWMH | 25 | .47120 | .016731 | .003346 | .46429 | .47811 | .437 | .495 |
|  | AD-moWMH | 20 | .38190 | .029663 | .006633 | .36802 | .39578 | .332 | .434 |
|  | 总计 | 102 | .85922 | .402620 | .039865 | .78013 | .93830 | .332 | 1.732 |
| 左侧顶中下回 | HC | 28 | 1.13825 | .159669 | .030175 | 1.07634 | 1.20016 | .970 | 1.822 |
|  | AD-nonWMH | 29 | 1.01697 | .095833 | .017796 | .98051 | 1.05342 | .879 | 1.277 |
|  | AD-miWMH | 25 | .84972 | .182405 | .036481 | .77443 | .92501 | .441 | 1.323 |
|  | AD-moWMH | 20 | .47460 | .089145 | .019933 | .43288 | .51632 | .323 | .656 |
|  | 总计 | 102 | .90292 | .273640 | .027094 | .84917 | .95667 | .323 | 1.822 |
| 右侧顶中下回 | HC | 28 | 1.18668 | .158545 | .029962 | 1.12520 | 1.24816 | .898 | 1.703 |
|  | AD-nonWMH | 29 | .99183 | .116362 | .021608 | .94757 | 1.03609 | .623 | 1.177 |
|  | AD-miWMH | 25 | .49076 | .100641 | .020128 | .44922 | .53230 | .383 | .774 |
|  | AD-moWMH | 20 | .42570 | .025431 | .005687 | .41380 | .43760 | .371 | .477 |
|  | 总计 | 102 | .81150 | .341026 | .033767 | .74452 | .87848 | .371 | 1.703 |
| 左侧顶后叶 | HC | 28 | 1.89082 | .208943 | .039487 | 1.80980 | 1.97184 | 1.442 | 2.219 |
|  | AD-nonWMH | 29 | 1.13838 | .201519 | .037421 | 1.06173 | 1.21503 | .857 | 1.571 |
|  | AD-miWMH | 25 | .47332 | .017296 | .003459 | .46618 | .48046 | .446 | .501 |
|  | AD-moWMH | 20 | .42285 | .019069 | .004264 | .41393 | .43177 | .388 | .454 |
|  | 总计 | 102 | 1.04163 | .617605 | .061152 | .92032 | 1.16294 | .388 | 2.219 |
| 右侧顶后叶 | HC | 28 | 1.81004 | .251702 | .047567 | 1.71244 | 1.90764 | 1.377 | 2.318 |
|  | AD-nonWMH | 29 | 1.12438 | .171599 | .031865 | 1.05911 | 1.18965 | .772 | 1.432 |
|  | AD-miWMH | 25 | .57284 | .140301 | .028060 | .51493 | .63075 | .460 | .882 |
|  | AD-moWMH | 20 | .42005 | .022922 | .005126 | .40932 | .43078 | .353 | .450 |
|  | 总计 | 102 | 1.03931 | .571340 | .056571 | .92709 | 1.15154 | .353 | 2.318 |

| **方差齐性检验** | | | | | |
| --- | --- | --- | --- | --- | --- |
|  | | 莱文统计 | 自由度 1 | 自由度 2 | 显著性 |
| 左侧角回 | 基于平均值 | .623 | 3 | 98 | .102 |
|  | 基于中位数 | .623 | 3 | 98 | .102 |
|  | 基于中位数并具有调整后自由度 | .623 | 3 | 86.154 | .102 |
|  | 基于剪除后平均值 | .614 | 3 | 98 | .108 |
| 右侧角回 | 基于平均值 | .270 | 3 | 98 | .847 |
|  | 基于中位数 | .218 | 3 | 98 | .884 |
|  | 基于中位数并具有调整后自由度 | .218 | 3 | 88.132 | .884 |
|  | 基于剪除后平均值 | .263 | 3 | 98 | .852 |
| 左侧楔前回 | 基于平均值 | 6.794 | 3 | 98 | .070 |
|  | 基于中位数 | 4.530 | 3 | 98 | .075 |
|  | 基于中位数并具有调整后自由度 | 4.530 | 3 | 92.789 | .075 |
|  | 基于剪除后平均值 | 6.745 | 3 | 98 | .070 |
| 右侧楔前回 | 基于平均值 | 1.100 | 3 | 98 | .003 |
|  | 基于中位数 | .682 | 3 | 98 | .065 |
|  | 基于中位数并具有调整后自由度 | .682 | 3 | 82.906 | .065 |
|  | 基于剪除后平均值 | 1.102 | 3 | 98 | .032 |
| 左侧顶上回 | 基于平均值 | 1.533 | 3 | 98 | .041 |
|  | 基于中位数 | 1.073 | 3 | 98 | .044 |
|  | 基于中位数并具有调整后自由度 | 1.073 | 3 | 88.968 | .035 |
|  | 基于剪除后平均值 | 1.455 | 3 | 98 | .032 |
| 右侧顶上回 | 基于平均值 | 12.772 | 3 | 98 | .000 |
|  | 基于中位数 | 11.716 | 3 | 98 | .000 |
|  | 基于中位数并具有调整后自由度 | 11.716 | 3 | 48.477 | .000 |
|  | 基于剪除后平均值 | 13.270 | 3 | 98 | .000 |
| 左侧顶中下回 | 基于平均值 | 1.441 | 3 | 98 | .036 |
|  | 基于中位数 | 1.355 | 3 | 98 | .041 |
|  | 基于中位数并具有调整后自由度 | 1.355 | 3 | 68.294 | .044 |
|  | 基于剪除后平均值 | 1.419 | 3 | 98 | .042 |
| 右侧顶中下回 | 基于平均值 | 6.506 | 3 | 98 | .107 |
|  | 基于中位数 | 5.539 | 3 | 98 | .101 |
|  | 基于中位数并具有调整后自由度 | 5.539 | 3 | 75.728 | .112 |
|  | 基于剪除后平均值 | 6.111 | 3 | 98 | .101 |
| 左侧顶后叶 | 基于平均值 | 24.435 | 3 | 98 | .000 |
|  | 基于中位数 | 20.236 | 3 | 98 | .000 |
|  | 基于中位数并具有调整后自由度 | 20.236 | 3 | 51.875 | .000 |
|  | 基于剪除后平均值 | 23.210 | 3 | 98 | .000 |
| 右侧顶后叶 | 基于平均值 | 15.992 | 3 | 98 | .000 |
|  | 基于中位数 | 12.431 | 3 | 98 | .000 |
|  | 基于中位数并具有调整后自由度 | 12.431 | 3 | 68.615 | .000 |
|  | 基于剪除后平均值 | 15.557 | 3 | 98 | .000 |

| **ANOVA** | | | | | | |
| --- | --- | --- | --- | --- | --- | --- |
|  | | 平方和 | 自由度 | 均方 | F | 显著性 |
| 左侧角回 | 组间 | 139.030 | 3 | 46.343 | 42.602 | .180 |
|  | 组内 | 106.606 | 98 | 1.088 |  |  |
|  | 总计 | 245.636 | 101 |  |  |  |
| 右侧角回 | 组间 | 159.942 | 3 | 53.314 | 54.861 | .207 |
|  | 组内 | 95.237 | 98 | .972 |  |  |
|  | 总计 | 255.179 | 101 |  |  |  |
| 左侧楔前回 | 组间 | 129.234 | 3 | 43.078 | 98.384 | .231 |
|  | 组内 | 42.910 | 98 | .438 |  |  |
|  | 总计 | 172.144 | 101 |  |  |  |
| 右侧楔前回 | 组间 | 187.583 | 3 | 62.528 | 143.404 | .060 |
|  | 组内 | 42.730 | 98 | .436 |  |  |
|  | 总计 | 230.313 | 101 |  |  |  |
| 左侧顶上回 | 组间 | 9.227 | 3 | 3.076 | 159.833 | .030 |
|  | 组内 | 1.886 | 98 | .019 |  |  |
|  | 总计 | 11.112 | 101 |  |  |  |
| 右侧顶上回 | 组间 | 15.392 | 3 | 5.131 | 512.711 | .000 |
|  | 组内 | .981 | 98 | .010 |  |  |
|  | 总计 | 16.372 | 101 |  |  |  |
| 左侧顶中下回 | 组间 | 5.668 | 3 | 1.889 | 97.702 | .000 |
|  | 组内 | 1.895 | 98 | .019 |  |  |
|  | 总计 | 7.563 | 101 |  |  |  |
| 右侧顶中下回 | 组间 | 10.433 | 3 | 3.478 | 259.530 | .177 |
|  | 组内 | 1.313 | 98 | .013 |  |  |
|  | 总计 | 11.746 | 101 |  |  |  |
| 左侧顶后叶 | 组间 | 36.195 | 3 | 12.065 | 507.477 | .000 |
|  | 组内 | 2.330 | 98 | .024 |  |  |
|  | 总计 | 38.525 | 101 |  |  |  |
| 右侧顶后叶 | 组间 | 29.952 | 3 | 9.984 | 324.255 | .000 |
|  | 组内 | 3.017 | 98 | .031 |  |  |
|  | 总计 | 32.969 | 101 |  |  |  |

| **多重比较** | | | | | | | | |
| --- | --- | --- | --- | --- | --- | --- | --- | --- |
| 因变量 | | (I) group | (J) group | 平均值差值 (I-J) | 标准 错误 | 显著性 | 95% 置信区间 | |
|  |  |  |  |  |  |  | 下限 | 上限 |
| 左侧角回 | LSD | HC | AD-nonWMH | .476745 | .276336 | .088 | -.07164 | 1.02513 |
|  |  |  | AD-miWMH | 1.174407 | .286991 | .090 | .60488 | 1.74393 |
|  |  |  | AD-moWMH | 3.266557 | .305355 | .055 | 2.66059 | 3.87252 |
|  |  | AD-nonWMH | HC | -.476745 | .276336 | .088 | -1.02513 | .07164 |
|  |  |  | AD-miWMH | .697662^*^ | .284647 | .016 | .13279 | 1.26253 |
|  |  |  | AD-moWMH | 2.789812 | .303154 | .079 | 2.18821 | 3.39141 |
|  |  | AD-miWMH | HC | -1.174407 | .286991 | .090 | -1.74393 | -.60488 |
|  |  |  | AD-nonWMH | -.697662^*^ | .284647 | .016 | -1.26253 | -.13279 |
|  |  |  | AD-moWMH | 2.092150 | .312896 | .080 | 1.47122 | 2.71308 |
|  |  | AD-moWMH | HC | -3.266557 | .305355 | .055 | -3.87252 | -2.66059 |
|  |  |  | AD-nonWMH | -2.789812 | .303154 | .079 | -3.39141 | -2.18821 |
|  |  |  | AD-miWMH | -2.092150 | .312896 | .080 | -2.71308 | -1.47122 |
|  | 塔姆黑尼 | HC | AD-nonWMH | .476745 | .267119 | .396 | -.25565 | 1.20914 |
|  |  |  | AD-miWMH | 1.174407 | .286749 | .201 | .38925 | 1.95957 |
|  |  |  | AD-moWMH | 3.266557 | .357983 | .090 | 2.27398 | 4.25913 |
|  |  | AD-nonWMH | HC | -.476745 | .267119 | .396 | -1.20914 | .25565 |
|  |  |  | AD-miWMH | .697662^*^ | .241411 | .034 | .03560 | 1.35972 |
|  |  |  | AD-moWMH | 2.789812 | .322810 | .077 | 1.88091 | 3.69871 |
|  |  | AD-miWMH | HC | -1.174407 | .286749 | .201 | -1.95957 | -.38925 |
|  |  |  | AD-nonWMH | -.697662^*^ | .241411 | .034 | -1.35972 | -.03560 |
|  |  |  | AD-moWMH | 2.092150 | .339233 | .082 | 1.14445 | 3.03985 |
|  |  | AD-moWMH | HC | -3.266557 | .357983 | .090 | -4.25913 | -2.27398 |
|  |  |  | AD-nonWMH | -2.789812 | .322810 | .077 | -3.69871 | -1.88091 |
|  |  |  | AD-miWMH | -2.092150 | .339233 | .082 | -3.03985 | -1.14445 |
| 右侧角回 | LSD | HC | AD-nonWMH | .805302 | .261186 | .203 | .28699 | 1.32362 |
|  |  |  | AD-miWMH | 1.572790 | .271256 | .095 | 1.03449 | 2.11109 |
|  |  |  | AD-moWMH | 3.576950 | .288614 | .130 | 3.00421 | 4.14969 |
|  |  | AD-nonWMH | HC | -.805302 | .261186 | .203 | -1.32362 | -.28699 |
|  |  |  | AD-miWMH | .767488 | .269041 | .255 | .23359 | 1.30139 |
|  |  |  | AD-moWMH | 2.771648 | .286533 | .090 | 2.20303 | 3.34026 |
|  |  | AD-miWMH | HC | -1.572790 | .271256 | .095 | -2.11109 | -1.03449 |
|  |  |  | AD-nonWMH | -.767488 | .269041 | .255 | -1.30139 | -.23359 |
|  |  |  | AD-moWMH | 2.004160 | .295741 | .122 | 1.41727 | 2.59105 |
|  |  | AD-moWMH | HC | -3.576950 | .288614 | .130 | -4.14969 | -3.00421 |
|  |  |  | AD-nonWMH | -2.771648 | .286533 | .090 | -3.34026 | -2.20303 |
|  |  |  | AD-miWMH | -2.004160 | .295741 | .122 | -2.59105 | -1.41727 |
|  | 塔姆黑尼 | HC | AD-nonWMH | .805302 | .245529 | .311 | .13418 | 1.47643 |
|  |  |  | AD-miWMH | 1.572790 | .297570 | .170 | .75627 | 2.38931 |
|  |  |  | AD-moWMH | 3.576950 | .282136 | .104 | 2.79865 | 4.35525 |
|  |  | AD-nonWMH | HC | -.805302 | .245529 | .311 | -1.47643 | -.13418 |
|  |  |  | AD-miWMH | .767488 | .277900 | .079 | .00150 | 1.53347 |
|  |  |  | AD-moWMH | 2.771648 | .261307 | .088 | 2.04659 | 3.49671 |
|  |  | AD-miWMH | HC | -1.572790 | .297570 | .170 | -2.38931 | -.75627 |
|  |  |  | AD-nonWMH | -.767488 | .277900 | .079 | -1.53347 | -.00150 |
|  |  |  | AD-moWMH | 2.004160 | .310717 | .104 | 1.14729 | 2.86103 |
|  |  | AD-moWMH | HC | -3.576950 | .282136 | .090 | -4.35525 | -2.79865 |
|  |  |  | AD-nonWMH | -2.771648 | .261307 | .088 | -3.49671 | -2.04659 |
|  |  |  | AD-miWMH | -2.004160 | .310717 | .090 | -2.86103 | -1.14729 |
| 左侧楔前回 | LSD | HC | AD-nonWMH | 1.157092 | .175318 | .133 | .80918 | 1.50500 |
|  |  |  | AD-miWMH | 2.053159 | .182077 | .272 | 1.69183 | 2.41448 |
|  |  |  | AD-moWMH | 3.164379 | .193728 | .110 | 2.77993 | 3.54883 |
|  |  | AD-nonWMH | HC | -1.157092 | .175318 | .133 | -1.50500 | -.80918 |
|  |  |  | AD-miWMH | .896066 | .180590 | .205 | .53769 | 1.25444 |
|  |  |  | AD-moWMH | 2.007286 | .192331 | .172 | 1.62561 | 2.38896 |
|  |  | AD-miWMH | HC | -2.053159 | .182077 | .272 | -2.41448 | -1.69183 |
|  |  |  | AD-nonWMH | -.896066 | .180590 | .205 | -1.25444 | -.53769 |
|  |  |  | AD-moWMH | 1.111220 | .198512 | .097 | .71728 | 1.50516 |
|  |  | AD-moWMH | HC | -3.164379 | .193728 | .110 | -3.54883 | -2.77993 |
|  |  |  | AD-nonWMH | -2.007286 | .192331 | .172 | -2.38896 | -1.62561 |
|  |  |  | AD-miWMH | -1.111220 | .198512 | .097 | -1.50516 | -.71728 |
|  | 塔姆黑尼 | HC | AD-nonWMH | 1.157092 | .201906 | .153 | .60193 | 1.71225 |
|  |  |  | AD-miWMH | 2.053159 | .199089 | .257 | 1.50449 | 2.60183 |
|  |  |  | AD-moWMH | 3.164379 | .206655 | .126 | 2.59532 | 3.73344 |
|  |  | AD-nonWMH | HC | -1.157092 | .201906 | .153 | -1.71225 | -.60193 |
|  |  |  | AD-miWMH | .896066 | .148651 | .188 | .48951 | 1.30263 |
|  |  |  | AD-moWMH | 2.007286 | .158641 | .142 | 1.57016 | 2.44441 |
|  |  | AD-miWMH | HC | -2.053159 | .199089 | .257 | -2.60183 | -1.50449 |
|  |  |  | AD-nonWMH | -.896066 | .148651 | .188 | -1.30263 | -.48951 |
|  |  |  | AD-moWMH | 1.111220 | .155039 | .103 | .68244 | 1.54000 |
|  |  | AD-moWMH | HC | -3.164379 | .206655 | .126 | -3.73344 | -2.59532 |
|  |  |  | AD-nonWMH | -2.007286 | .158641 | .142 | -2.44441 | -1.57016 |
|  |  |  | AD-miWMH | -1.111220 | .155039 | .103 | -1.54000 | -.68244 |
| 右侧楔前回 | LSD | HC | AD-nonWMH | 1.191216 | .174951 | .122 | .84403 | 1.53840 |
|  |  |  | AD-miWMH | 2.652130 | .181696 | .108 | 2.29156 | 3.01270 |
|  |  |  | AD-moWMH | 3.655700 | .193322 | .090 | 3.27206 | 4.03934 |
|  |  | AD-nonWMH | HC | -1.191216 | .174951 | .122 | -1.53840 | -.84403 |
|  |  |  | AD-miWMH | 1.460914 | .180212 | .171 | 1.10329 | 1.81854 |
|  |  |  | AD-moWMH | 2.464484^*^ | .191929 | .000 | 2.08361 | 2.84536 |
|  |  | AD-miWMH | HC | -2.652130 | .181696 | .108 | -3.01270 | -2.29156 |
|  |  |  | AD-nonWMH | -1.460914 | .180212 | .177 | -1.81854 | -1.10329 |
|  |  |  | AD-moWMH | 1.003570 | .198097 | .088 | .61045 | 1.39669 |
|  |  | AD-moWMH | HC | -3.655700 | .193322 | .090 | -4.03934 | -3.27206 |
|  |  |  | AD-nonWMH | -2.464484^*^ | .191929 | .000 | -2.84536 | -2.08361 |
|  |  |  | AD-miWMH | -1.003570 | .198097 | .088 | -1.39669 | -.61045 |
|  | 塔姆黑尼 | HC | AD-nonWMH | 1.191216 | .164587 | .122 | .74205 | 1.64038 |
|  |  |  | AD-miWMH | 2.652130 | .184705 | .108 | 2.14506 | 3.15920 |
|  |  |  | AD-moWMH | 3.655700 | .192907 | .090 | 3.12031 | 4.19109 |
|  |  | AD-nonWMH | HC | -1.191216 | .164587 | .122 | -1.64038 | -.74205 |
|  |  |  | AD-miWMH | 1.460914 | .185517 | .171 | .95194 | 1.96988 |
|  |  |  | AD-moWMH | 2.464484^*^ | .193686 | .000 | 1.92736 | 3.00161 |
|  |  | AD-miWMH | HC | -2.652130 | .184705 | .108 | -3.15920 | -2.14506 |
|  |  |  | AD-nonWMH | -1.460914 | .185517 | .177 | -1.96988 | -.95194 |
|  |  |  | AD-moWMH | 1.003570 | .211048 | .088 | .42060 | 1.58654 |
|  |  | AD-moWMH | HC | -3.655700 | .192907 | .090 | -4.19109 | -3.12031 |
|  |  |  | AD-nonWMH | -2.464484^*^ | .193686 | .000 | -3.00161 | -1.92736 |
|  |  |  | AD-miWMH | -1.003570 | .211048 | .088 | -1.58654 | -.42060 |
| 左侧顶上回 | LSD | HC | AD-nonWMH | .174916 | .036752 | .680 | .10198 | .24785 |
|  |  |  | AD-miWMH | .572211 | .038169 | .255 | .49647 | .64796 |
|  |  |  | AD-moWMH | .778871 | .040612 | .175 | .69828 | .85946 |
|  |  | AD-nonWMH | HC | -.174916 | .036752 | .680 | -.24785 | -.10198 |
|  |  |  | AD-miWMH | .397295 | .037858 | .247 | .32217 | .47242 |
|  |  |  | AD-moWMH | .603955 | .040319 | .139 | .52394 | .68397 |
|  |  | AD-miWMH | HC | -.572211 | .038169 | 255 | -.64796 | -.49647 |
|  |  |  | AD-nonWMH | -.397295 | .037858 | .247 | -.47242 | -.32217 |
|  |  |  | AD-moWMH | .206660^*^ | .041615 | .010 | .12408 | .28924 |
|  |  | AD-moWMH | HC | -.778871 | .040612 | .175 | -.85946 | -.69828 |
|  |  |  | AD-nonWMH | -.603955 | .040319 | .139 | -.68397 | -.52394 |
|  |  |  | AD-miWMH | -.206660^*^ | .041615 | .010 | -.28924 | -.12408 |
|  | 塔姆黑尼 | HC | AD-nonWMH | .174916 | .037214 | .680 | .07332 | .27651 |
|  |  |  | AD-miWMH | .572211 | .035130 | .255 | .47598 | .66845 |
|  |  |  | AD-moWMH | .778871 | .045641 | .175 | .65217 | .90557 |
|  |  | AD-nonWMH | HC | -.174916 | .037214 | .680 | -.27651 | -.07332 |
|  |  |  | AD-miWMH | .397295 | .033629 | .247 | .30531 | .48928 |
|  |  |  | AD-moWMH | .603955 | .044495 | .139 | .48009 | .72782 |
|  |  | AD-miWMH | HC | -.572211 | .035130 | 255 | -.66845 | -.47598 |
|  |  |  | AD-nonWMH | -.397295 | .033629 | .247 | -.48928 | -.30531 |
|  |  |  | AD-moWMH | .206660^*^ | .042767 | .010 | .08676 | .32656 |
|  |  | AD-moWMH | HC | -.778871 | .045641 | .175 | -.90557 | -.65217 |
|  |  |  | AD-nonWMH | -.603955 | .044495 | .139 | -.72782 | -.48009 |
|  |  |  | AD-miWMH | -.206660^*^ | .042767 | .010 | -.32656 | -.08676 |
| 右侧顶上回 | LSD | HC | AD-nonWMH | .200443 | .026504 | .088 | .14785 | .25304 |
|  |  |  | AD-miWMH | .827657 | .027526 | .068 | .77303 | .88228 |
|  |  |  | AD-moWMH | .916957 | .029287 | .053 | .85884 | .97508 |
|  |  | AD-nonWMH | HC | -.200443 | .026504 | .070 | -.25304 | -.14785 |
|  |  |  | AD-miWMH | .627214^*^ | .027301 | .000 | .57304 | .68139 |
|  |  |  | AD-moWMH | .716514^*^ | .029076 | .000 | .65881 | .77421 |
|  |  | AD-miWMH | HC | -.827657 | .027526 | .068 | -.88228 | -.77303 |
|  |  |  | AD-nonWMH | -.627214^*^ | .027301 | .000 | -.68139 | -.57304 |
|  |  |  | AD-moWMH | .089300 | .030010 | .074 | .02975 | .14885 |
|  |  | AD-moWMH | HC | -.916957 | .029287 | .053 | -.97508 | -.85884 |
|  |  |  | AD-nonWMH | -.716514^*^ | .029076 | .000 | -.77421 | -.65881 |
|  |  |  | AD-miWMH | -.089300 | .030010 | .074 | -.14885 | -.02975 |
|  | 塔姆黑尼 | HC | AD-nonWMH | .200443 | .035067 | .088 | .10458 | .29631 |
|  |  |  | AD-miWMH | .827657 | .027353 | .068 | .75021 | .90511 |
|  |  |  | AD-moWMH | .916957 | .027946 | .053 | .83829 | .99562 |
|  |  | AD-nonWMH | HC | -.200443 | .035067 | .070 | -.29631 | -.10458 |
|  |  |  | AD-miWMH | .627214^*^ | .022448 | .000 | .56389 | .69054 |
|  |  |  | AD-moWMH | .716514^*^ | .023167 | .000 | .65167 | .78136 |
|  |  | AD-miWMH | HC | -.827657 | .027353 | .068 | -.90511 | -.75021 |
|  |  |  | AD-nonWMH | -.627214^*^ | .022448 | .000 | -.69054 | -.56389 |
|  |  |  | AD-moWMH | .089300 | .007429 | .074 | .06830 | .11030 |
|  |  | AD-moWMH | HC | -.916957 | .027946 | .053 | -.99562 | -.83829 |
|  |  |  | AD-nonWMH | -.716514^*^ | .023167 | .000 | -.78136 | -.65167 |
|  |  |  | AD-miWMH | -.089300 | .007429 | .074 | -.11030 | -.06830 |
| 左侧顶中下回 | LSD | HC | AD-nonWMH | .121284^*^ | .036843 | .001 | .04817 | .19440 |
|  |  |  | AD-miWMH | .288530^*^ | .038263 | .000 | .21260 | .36446 |
|  |  |  | AD-moWMH | .663650^*^ | .040712 | .000 | .58286 | .74444 |
|  |  | AD-nonWMH | HC | -.121284^*^ | .036843 | .001 | -.19440 | -.04817 |
|  |  |  | AD-miWMH | .167246 | .037951 | .057 | .09193 | .24256 |
|  |  |  | AD-moWMH | .542366^*^ | .040418 | .000 | .46216 | .62257 |
|  |  | AD-miWMH | HC | -.288530^*^ | .038263 | .000 | -.36446 | -.21260 |
|  |  |  | AD-nonWMH | -.167246 | .037951 | .057 | -.24256 | -.09193 |
|  |  |  | AD-moWMH | .375120^*^ | .041717 | .000 | .29233 | .45791 |
|  |  | AD-moWMH | HC | -.663650^*^ | .040712 | .000 | -.74444 | -.58286 |
|  |  |  | AD-nonWMH | -.542366^*^ | .040418 | .000 | -.62257 | -.46216 |
|  |  |  | AD-miWMH | -.375120^*^ | .041717 | .000 | -.45791 | -.29233 |
|  | 塔姆黑尼 | HC | AD-nonWMH | .121284^*^ | .035031 | .007 | .02478 | .21779 |
|  |  |  | AD-miWMH | .288530^*^ | .047343 | .000 | .15863 | .41843 |
|  |  |  | AD-moWMH | .663650^*^ | .036164 | .000 | .56401 | .76329 |
|  |  | AD-nonWMH | HC | -.121284^*^ | .035031 | .007 | -.21779 | -.02478 |
|  |  |  | AD-miWMH | .167246 | .040590 | .057 | .05409 | .28040 |
|  |  |  | AD-moWMH | .542366^*^ | .026721 | .000 | .46867 | .61606 |
|  |  | AD-miWMH | HC | -.288530^*^ | .047343 | .000 | -.41843 | -.15863 |
|  |  |  | AD-nonWMH | -.167246 | .040590 | .057 | -.28040 | -.05409 |
|  |  |  | AD-moWMH | .375120^*^ | .041572 | .000 | .25947 | .49077 |
|  |  | AD-moWMH | HC | -.663650^*^ | .036164 | .000 | -.76329 | -.56401 |
|  |  |  | AD-nonWMH | -.542366^*^ | .026721 | .000 | -.61606 | -.46867 |
|  |  |  | AD-miWMH | -.375120^*^ | .041572 | .000 | -.49077 | -.25947 |
| 右侧顶中下回 | LSD | HC | AD-nonWMH | .194851^*^ | .030670 | .000 | .13399 | .25571 |
|  |  |  | AD-miWMH | .695919^*^ | .031852 | .020 | .63271 | .75913 |
|  |  |  | AD-moWMH | .760979^*^ | .033890 | .009 | .69372 | .82823 |
|  |  | AD-nonWMH | HC | -.194851^*^ | .030670 | .000 | -.25571 | -.13399 |
|  |  |  | AD-miWMH | .501068^*^ | .031592 | .000 | .43837 | .56376 |
|  |  |  | AD-moWMH | .566128^*^ | .033646 | .000 | .49936 | .63290 |
|  |  | AD-miWMH | HC | -.695919^*^ | .031852 | .020 | -.75913 | -.63271 |
|  |  |  | AD-nonWMH | -.501068^*^ | .031592 | .000 | -.56376 | -.43837 |
|  |  |  | AD-moWMH | .065060 | .034727 | .064 | -.00386 | .13398 |
|  |  | AD-moWMH | HC | -.760979^*^ | .033890 | .009 | -.82823 | -.69372 |
|  |  |  | AD-nonWMH | -.566128^*^ | .033646 | .000 | -.63290 | -.49936 |
|  |  |  | AD-miWMH | -.065060 | .034727 | .064 | -.13398 | .00386 |
|  | 塔姆黑尼 | HC | AD-nonWMH | .194851^*^ | .036941 | .000 | .09362 | .29608 |
|  |  |  | AD-miWMH | .695919^*^ | .036095 | .020 | .59672 | .79512 |
|  |  |  | AD-moWMH | .760979^*^ | .030497 | .009 | .67488 | .84708 |
|  |  | AD-nonWMH | HC | -.194851^*^ | .036941 | .000 | -.29608 | -.09362 |
|  |  |  | AD-miWMH | .501068^*^ | .029530 | .000 | .42030 | .58183 |
|  |  |  | AD-moWMH | .566128^*^ | .022344 | .000 | .50345 | .62880 |
|  |  | AD-miWMH | HC | -.695919^*^ | .036095 | .020 | -.79512 | -.59672 |
|  |  |  | AD-nonWMH | -.501068^*^ | .029530 | .000 | -.58183 | -.42030 |
|  |  |  | AD-moWMH | .065060^*^ | .020916 | .064 | .00583 | .12429 |
|  |  | AD-moWMH | HC | -.760979^*^ | .030497 | .009 | -.84708 | -.67488 |
|  |  |  | AD-nonWMH | -.566128^*^ | .022344 | .000 | -.62880 | -.50345 |
|  |  |  | AD-miWMH | -.065060^*^ | .020916 | .064 | -.12429 | -.00583 |
| 左侧顶后叶 | LSD | HC | AD-nonWMH | .752442^*^ | .040852 | .000 | .67137 | .83351 |
|  |  |  | AD-miWMH | 1.417501^*^ | .042427 | .000 | 1.33331 | 1.50170 |
|  |  |  | AD-moWMH | 1.467971^*^ | .045142 | .000 | 1.37839 | 1.55755 |
|  |  | AD-nonWMH | HC | -.752442^*^ | .040852 | .000 | -.83351 | -.67137 |
|  |  |  | AD-miWMH | .665059^*^ | .042081 | .000 | .58155 | .74857 |
|  |  |  | AD-moWMH | .715529^*^ | .044817 | .000 | .62659 | .80447 |
|  |  | AD-miWMH | HC | -1.417501^*^ | .042427 | .000 | -1.50170 | -1.33331 |
|  |  |  | AD-nonWMH | -.665059^*^ | .042081 | .000 | -.74857 | -.58155 |
|  |  |  | AD-moWMH | .050470 | .046257 | .078 | -.04133 | .14227 |
|  |  | AD-moWMH | HC | -1.467971^*^ | .045142 | .000 | -1.55755 | -1.37839 |
|  |  |  | AD-nonWMH | -.715529^*^ | .044817 | .000 | -.80447 | -.62659 |
|  |  |  | AD-miWMH | -.050470 | .046257 | .078 | -.14227 | .04133 |
|  | 塔姆黑尼 | HC | AD-nonWMH | .752442^*^ | .054402 | .000 | .60395 | .90094 |
|  |  |  | AD-miWMH | 1.417501^*^ | .039638 | .000 | 1.30514 | 1.52986 |
|  |  |  | AD-moWMH | 1.467971^*^ | .039716 | .000 | 1.35545 | 1.58049 |
|  |  | AD-nonWMH | HC | -.752442^*^ | .054402 | .000 | -.90094 | -.60395 |
|  |  |  | AD-miWMH | .665059^*^ | .037581 | .000 | .55883 | .77128 |
|  |  |  | AD-moWMH | .715529^*^ | .037663 | .000 | .60914 | .82192 |
|  |  | AD-miWMH | HC | -1.417501^*^ | .039638 | .000 | -1.52986 | -1.30514 |
|  |  |  | AD-nonWMH | -.665059^*^ | .037581 | .000 | -.77128 | -.55883 |
|  |  |  | AD-moWMH | .050470^*^ | .005491 | .000 | .03525 | .06569 |
|  |  | AD-moWMH | HC | -1.467971^*^ | .039716 | .000 | -1.58049 | -1.35545 |
|  |  |  | AD-nonWMH | -.715529^*^ | .037663 | .000 | -.82192 | -.60914 |
|  |  |  | AD-miWMH | -.050470^*^ | .005491 | .000 | -.06569 | -.03525 |
| 右侧顶后叶 | LSD | HC | AD-nonWMH | .685656^*^ | .046491 | .000 | .59340 | .77792 |
|  |  |  | AD-miWMH | 1.237196^*^ | .048283 | .000 | 1.14138 | 1.33301 |
|  |  |  | AD-moWMH | 1.389986^*^ | .051373 | .000 | 1.28804 | 1.49193 |
|  |  | AD-nonWMH | HC | -.685656^*^ | .046491 | .000 | -.77792 | -.59340 |
|  |  |  | AD-miWMH | .551539^*^ | .047889 | .000 | .45651 | .64657 |
|  |  |  | AD-moWMH | .704329^*^ | .051003 | .000 | .60312 | .80554 |
|  |  | AD-miWMH | HC | -1.237196^*^ | .048283 | .000 | -1.33301 | -1.14138 |
|  |  |  | AD-nonWMH | -.551539^*^ | .047889 | .000 | -.64657 | -.45651 |
|  |  |  | AD-moWMH | .152790^*^ | .052642 | .005 | .04832 | .25726 |
|  |  | AD-moWMH | HC | -1.389986^*^ | .051373 | .000 | -1.49193 | -1.28804 |
|  |  |  | AD-nonWMH | -.704329^*^ | .051003 | .000 | -.80554 | -.60312 |
|  |  |  | AD-miWMH | -.152790^*^ | .052642 | .005 | -.25726 | -.04832 |
|  | 塔姆黑尼 | HC | AD-nonWMH | .685656^*^ | .057254 | .000 | .52848 | .84284 |
|  |  |  | AD-miWMH | 1.237196^*^ | .055227 | .000 | 1.08493 | 1.38946 |
|  |  |  | AD-moWMH | 1.389986^*^ | .047843 | .000 | 1.25445 | 1.52553 |
|  |  | AD-nonWMH | HC | -.685656^*^ | .057254 | .000 | -.84284 | -.52848 |
|  |  |  | AD-miWMH | .551539^*^ | .042459 | .000 | .43541 | .66767 |
|  |  |  | AD-moWMH | .704329^*^ | .032275 | .000 | .61332 | .79534 |
|  |  | AD-miWMH | HC | -1.237196^*^ | .055227 | .000 | -1.38946 | -1.08493 |
|  |  |  | AD-nonWMH | -.551539^*^ | .042459 | .000 | -.66767 | -.43541 |
|  |  |  | AD-moWMH | .152790^*^ | .028524 | .000 | .07149 | .23409 |
|  |  | AD-moWMH | HC | -1.389986^*^ | .047843 | .000 | -1.52553 | -1.25445 |
|  |  |  | AD-nonWMH | -.704329^*^ | .032275 | .000 | -.79534 | -.61332 |
|  |  |  | AD-miWMH | -.152790^*^ | .028524 | .000 | -.23409 | -.07149 |
| *. 平均值差值的显著性水平为 0.05。 | | | | | | | | |

| **描述** | | | | | | | | | |
| --- | --- | --- | --- | --- | --- | --- | --- | --- | --- |
|  | | 个案数 | 平均值 | 标准 偏差 | 标准 错误 | 平均值的 95% 置信区间 | | 最小值 | 最大值 |
|  |  |  |  |  |  | 下限 | 上限 |  |  |
| 左侧缘上回 | HC | 28 | 43.70896 | 2.593737 | .490170 | 42.70322 | 44.71471 | 35.329 | 47.103 |
|  | AD-nonWMH | 29 | 34.92466 | 1.461063 | .271313 | 34.36890 | 35.48041 | 31.329 | 38.103 |
|  | AD-miWMH | 25 | 29.06740 | 3.950474 | .790095 | 27.43672 | 30.69808 | 22.743 | 37.021 |
|  | AD-moWMH | 20 | 23.70395 | 2.293400 | .512820 | 22.63061 | 24.77729 | 18.830 | 27.843 |
|  | 总计 | 102 | 33.70029 | 7.771080 | .769452 | 32.17391 | 35.22668 | 18.830 | 47.103 |
| 右侧缘上回 | HC | 28 | 44.51825 | 3.024256 | .571531 | 43.34557 | 45.69093 | 38.288 | 48.553 |
|  | AD-nonWMH | 29 | 34.24214 | 2.397653 | .445233 | 33.33012 | 35.15416 | 29.440 | 38.504 |
|  | AD-miWMH | 25 | 30.00076 | 3.358392 | .671678 | 28.61448 | 31.38704 | 21.768 | 37.577 |
|  | AD-moWMH | 20 | 23.90660 | 3.351954 | .749520 | 22.33784 | 25.47536 | 17.744 | 29.443 |
|  | 总计 | 102 | 33.99690 | 7.978250 | .789964 | 32.42982 | 35.56398 | 17.744 | 48.553 |
| 左侧枕上回 | HC | 28 | 13.81739 | 1.632113 | .308440 | 13.18453 | 14.45026 | 9.409 | 16.724 |
|  | AD-nonWMH | 29 | 8.81393 | 1.072884 | .199230 | 8.40583 | 9.22203 | 6.958 | 11.239 |
|  | AD-miWMH | 25 | 6.75888 | 1.093645 | .218729 | 6.30745 | 7.21031 | 4.433 | 8.434 |
|  | AD-moWMH | 20 | 5.51350 | 1.132250 | .253179 | 4.98359 | 6.04341 | 4.010 | 7.284 |
|  | 总计 | 102 | 9.03660 | 3.413088 | .337946 | 8.36620 | 9.70699 | 4.010 | 16.724 |
| 右侧枕上回 | HC | 28 | 12.65657 | 1.188444 | .224595 | 12.19574 | 13.11740 | 10.293 | 14.942 |
|  | AD-nonWMH | 29 | 10.96048 | 1.403633 | .260648 | 10.42657 | 11.49440 | 8.943 | 13.984 |
|  | AD-miWMH | 25 | 8.91228 | 1.013922 | .202784 | 8.49375 | 9.33081 | 6.392 | 11.283 |
|  | AD-moWMH | 20 | 7.74185 | 1.322515 | .295723 | 7.12289 | 8.36081 | 5.093 | 11.330 |
|  | 总计 | 102 | 10.29296 | 2.221349 | .219946 | 9.85665 | 10.72928 | 5.093 | 14.942 |
| 左侧枕中回 | HC | 28 | 15.65679 | .765934 | .144748 | 15.35979 | 15.95378 | 14.398 | 17.549 |
|  | AD-nonWMH | 29 | 12.10024 | 1.017449 | .188935 | 11.71322 | 12.48726 | 10.032 | 13.921 |
|  | AD-miWMH | 25 | 11.51240 | 1.040848 | .208170 | 11.08276 | 11.94204 | 10.288 | 13.283 |
|  | AD-moWMH | 20 | 10.26570 | 1.006630 | .225089 | 9.79458 | 10.73682 | 8.869 | 12.930 |
|  | 总计 | 102 | 12.57275 | 2.219239 | .219737 | 12.13685 | 13.00865 | 8.869 | 17.549 |
| 右侧枕中回 | HC | 28 | 14.83561 | 1.147386 | .216836 | 14.39070 | 15.28052 | 12.578 | 17.277 |
|  | AD-nonWMH | 29 | 14.87031 | 1.140787 | .211839 | 14.43638 | 15.30424 | 13.430 | 16.954 |
|  | AD-miWMH | 25 | 14.45424 | 1.098791 | .219758 | 14.00068 | 14.90780 | 11.876 | 16.943 |
|  | AD-moWMH | 20 | 14.55880 | .937051 | .209531 | 14.12025 | 14.99735 | 12.849 | 15.920 |
|  | 总计 | 102 | 14.69773 | 1.094125 | .108335 | 14.48282 | 14.91263 | 11.876 | 17.277 |

| **方差齐性检验** | | | | | |
| --- | --- | --- | --- | --- | --- |
|  | | 莱文统计 | 自由度 1 | 自由度 2 | 显著性 |
| 左侧缘上回 | 基于平均值 | 9.706 | 3 | 98 | .119 |
|  | 基于中位数 | 5.970 | 3 | 98 | .122 |
|  | 基于中位数并具有调整后自由度 | 5.970 | 3 | 64.813 | .122 |
|  | 基于剪除后平均值 | 9.162 | 3 | 98 | .119 |
| 右侧缘上回 | 基于平均值 | .818 | 3 | 98 | .487 |
|  | 基于中位数 | .737 | 3 | 98 | .532 |
|  | 基于中位数并具有调整后自由度 | .737 | 3 | 90.258 | .533 |
|  | 基于剪除后平均值 | .811 | 3 | 98 | .491 |
| 左侧枕上回 | 基于平均值 | .666 | 3 | 98 | .575 |
|  | 基于中位数 | .443 | 3 | 98 | .723 |
|  | 基于中位数并具有调整后自由度 | .443 | 3 | 66.343 | .723 |
|  | 基于剪除后平均值 | .573 | 3 | 98 | .634 |
| 右侧枕上回 | 基于平均值 | 1.172 | 3 | 98 | .324 |
|  | 基于中位数 | .830 | 3 | 98 | .480 |
|  | 基于中位数并具有调整后自由度 | .830 | 3 | 89.665 | .481 |
|  | 基于剪除后平均值 | 1.125 | 3 | 98 | .343 |
| 左侧枕中回 | 基于平均值 | 2.013 | 3 | 98 | .117 |
|  | 基于中位数 | 1.908 | 3 | 98 | .133 |
|  | 基于中位数并具有调整后自由度 | 1.908 | 3 | 92.593 | .134 |
|  | 基于剪除后平均值 | 2.047 | 3 | 98 | .112 |
| 右侧枕中回 | 基于平均值 | .180 | 3 | 98 | .910 |
|  | 基于中位数 | .103 | 3 | 98 | .958 |
|  | 基于中位数并具有调整后自由度 | .103 | 3 | 89.769 | .958 |
|  | 基于剪除后平均值 | .159 | 3 | 98 | .924 |

| **ANOVA** | | | | | | |
| --- | --- | --- | --- | --- | --- | --- |
|  | | 平方和 | 自由度 | 均方 | F | 显著性 |
| 左侧缘上回 | 组间 | 5383.461 | 3 | 1794.487 | 245.649 | .072 |
|  | 组内 | 715.897 | 98 | 7.305 |  |  |
|  | 总计 | 6099.358 | 101 |  |  |  |
| 右侧缘上回 | 组间 | 5536.822 | 3 | 1845.607 | 202.751 | .066 |
|  | 组内 | 892.077 | 98 | 9.103 |  |  |
|  | 总计 | 6428.900 | 101 |  |  |  |
| 左侧枕上回 | 组间 | 1019.350 | 3 | 339.783 | 211.803 | .172 |
|  | 组内 | 157.216 | 98 | 1.604 |  |  |
|  | 总计 | 1176.566 | 101 |  |  |  |
| 右侧枕上回 | 组间 | 347.169 | 3 | 115.723 | 75.003 | .059 |
|  | 组内 | 151.205 | 98 | 1.543 |  |  |
|  | 总计 | 498.373 | 101 |  |  |  |
| 左侧枕中回 | 组间 | 407.349 | 3 | 135.783 | 147.723 | .620 |
|  | 组内 | 90.079 | 98 | .919 |  |  |
|  | 总计 | 497.427 | 101 |  |  |  |
| 右侧枕中回 | 组间 | 3.264 | 3 | 1.088 | .906 | .441 |
|  | 组内 | 117.644 | 98 | 1.200 |  |  |
|  | 总计 | 120.908 | 101 |  |  |  |

| **多重比较** | | | | | | | | |
| --- | --- | --- | --- | --- | --- | --- | --- | --- |
| 因变量 | | (I) group | (J) group | 平均值差值 (I-J) | 标准 错误 | 显著性 | 95% 置信区间 | |
|  |  |  |  |  |  |  | 下限 | 上限 |
| 左侧缘上回 | LSD | HC | AD-nonWMH | 8.784309 | .716097 | .127 | 7.36324 | 10.20538 |
|  |  |  | AD-miWMH | 14.641564 | .743706 | .306 | 13.16570 | 16.11742 |
|  |  |  | AD-moWMH | 20.005014 | .791296 | .092 | 18.43471 | 21.57532 |
|  |  | AD-nonWMH | HC | -8.784309 | .716097 | .127 | -10.20538 | -7.36324 |
|  |  |  | AD-miWMH | 5.857255 | .737633 | .088 | 4.39345 | 7.32106 |
|  |  |  | AD-moWMH | 11.220705 | .785591 | .070 | 9.66173 | 12.77968 |
|  |  | AD-miWMH | HC | -14.641564 | .743706 | .306 | -16.11742 | -13.16570 |
|  |  |  | AD-nonWMH | -5.857255 | .737633 | .088 | -7.32106 | -4.39345 |
|  |  |  | AD-moWMH | 5.363450 | .810837 | .121 | 3.75437 | 6.97253 |
|  |  | AD-moWMH | HC | -20.005014 | .791296 | .092 | -21.57532 | -18.43471 |
|  |  |  | AD-nonWMH | -11.220705 | .785591 | .070 | -12.77968 | -9.66173 |
|  |  |  | AD-miWMH | -5.363450 | .810837 | .121 | -6.97253 | -3.75437 |
|  | 塔姆黑尼 | HC | AD-nonWMH | 8.784309 | .560248 | .127 | 7.23804 | 10.33058 |
|  |  |  | AD-miWMH | 14.641564 | .929794 | .306 | 12.07048 | 17.21265 |
|  |  |  | AD-moWMH | 20.005014 | .709402 | .092 | 18.05052 | 21.95951 |
|  |  | AD-nonWMH | HC | -8.784309 | .560248 | .127 | -10.33058 | -7.23804 |
|  |  |  | AD-miWMH | 5.857255 | .835380 | .088 | 3.50281 | 8.21170 |
|  |  |  | AD-moWMH | 11.220705 | .580168 | .070 | 9.58521 | 12.85620 |
|  |  | AD-miWMH | HC | -14.641564 | .929794 | .306 | -17.21265 | -12.07048 |
|  |  |  | AD-nonWMH | -5.857255 | .835380 | .088 | -8.21170 | -3.50281 |
|  |  |  | AD-moWMH | 5.363450 | .941931 | .121 | 2.75522 | 7.97168 |
|  |  | AD-moWMH | HC | -20.005014 | .709402 | .092 | -21.95951 | -18.05052 |
|  |  |  | AD-nonWMH | -11.220705 | .580168 | .070 | -12.85620 | -9.58521 |
|  |  |  | AD-miWMH | -5.363450 | .941931 | .121 | -7.97168 | -2.75522 |
| 右侧缘上回 | LSD | HC | AD-nonWMH | 10.276112 | .799370 | .082 | 8.68979 | 11.86244 |
|  |  |  | AD-miWMH | 14.517490 | .830189 | .119 | 12.87001 | 16.16497 |
|  |  |  | AD-moWMH | 20.611650 | .883313 | .079 | 18.85874 | 22.36456 |
|  |  | AD-nonWMH | HC | -10.276112 | .799370 | .082 | -11.86244 | -8.68979 |
|  |  |  | AD-miWMH | 4.241378 | .823410 | .217 | 2.60735 | 5.87541 |
|  |  |  | AD-moWMH | 10.335538 | .876945 | .094 | 8.59527 | 12.07581 |
|  |  | AD-miWMH | HC | -14.517490 | .830189 | .119 | -16.16497 | -12.87001 |
|  |  |  | AD-nonWMH | -4.241378 | .823410 | .217 | -5.87541 | -2.60735 |
|  |  |  | AD-moWMH | 6.094160 | .905127 | .120 | 4.29796 | 7.89036 |
|  |  | AD-moWMH | HC | -20.611650 | .883313 | .079 | -22.36456 | -18.85874 |
|  |  |  | AD-nonWMH | -10.335538 | .876945 | .094 | -12.07581 | -8.59527 |
|  |  |  | AD-miWMH | -6.094160 | .905127 | .120 | -7.89036 | -4.29796 |
|  | 塔姆黑尼 | HC | AD-nonWMH | 10.276112^*^ | .724486 | .082 | 8.29385 | 12.25837 |
|  |  |  | AD-miWMH | 14.517490^*^ | .881929 | .119 | 12.09894 | 16.93604 |
|  |  |  | AD-moWMH | 20.611650^*^ | .942564 | .079 | 17.99734 | 23.22596 |
|  |  | AD-nonWMH | HC | -10.276112^*^ | .724486 | .082 | -12.25837 | -8.29385 |
|  |  |  | AD-miWMH | 4.241378^*^ | .805844 | .217 | 2.01831 | 6.46444 |
|  |  |  | AD-moWMH | 10.335538^*^ | .871787 | .094 | 7.89157 | 12.77951 |
|  |  | AD-miWMH | HC | -14.517490^*^ | .881929 | .119 | -16.93604 | -12.09894 |
|  |  |  | AD-nonWMH | -4.241378^*^ | .805844 | .217 | -6.46444 | -2.01831 |
|  |  |  | AD-moWMH | 6.094160^*^ | 1.006445 | .120 | 3.31187 | 8.87645 |
|  |  | AD-moWMH | HC | -20.611650^*^ | .942564 | .079 | -23.22596 | -17.99734 |
|  |  |  | AD-nonWMH | -10.335538^*^ | .871787 | .094 | -12.77951 | -7.89157 |
|  |  |  | AD-miWMH | -6.094160^*^ | 1.006445 | .120 | -8.87645 | -3.31187 |
| 左侧枕上回 | LSD | HC | AD-nonWMH | 5.003462 | .335579 | .170 | 4.33752 | 5.66941 |
|  |  |  | AD-miWMH | 7.058513 | .348517 | .227 | 6.36689 | 7.75013 |
|  |  |  | AD-moWMH | 8.303893 | .370819 | .311 | 7.56801 | 9.03977 |
|  |  | AD-nonWMH | HC | -5.003462 | .335579 | .170 | -5.66941 | -4.33752 |
|  |  |  | AD-miWMH | 2.055051 | .345671 | .190 | 1.36908 | 2.74102 |
|  |  |  | AD-moWMH | 3.300431 | .368145 | .431 | 2.56986 | 4.03100 |
|  |  | AD-miWMH | HC | -7.058513 | .348517 | .227 | -7.75013 | -6.36689 |
|  |  |  | AD-nonWMH | -2.055051 | .345671 | .190 | -2.74102 | -1.36908 |
|  |  |  | AD-moWMH | 1.245380 | .379976 | .091 | .49133 | 1.99943 |
|  |  | AD-moWMH | HC | -8.303893 | .370819 | .311 | -9.03977 | -7.56801 |
|  |  |  | AD-nonWMH | -3.300431 | .368145 | .431 | -4.03100 | -2.56986 |
|  |  |  | AD-miWMH | -1.245380 | .379976 | .091 | -1.99943 | -.49133 |
|  | 塔姆黑尼 | HC | AD-nonWMH | 5.003462 | .367189 | .170 | 3.99447 | 6.01246 |
|  |  |  | AD-miWMH | 7.058513 | .378124 | .227 | 6.02048 | 8.09655 |
|  |  |  | AD-moWMH | 8.303893 | .399043 | .311 | 7.20689 | 9.40090 |
|  |  | AD-nonWMH | HC | -5.003462 | .367189 | .170 | -6.01246 | -3.99447 |
|  |  |  | AD-miWMH | 2.055051 | .295863 | .190 | 1.24496 | 2.86514 |
|  |  |  | AD-moWMH | 3.300431 | .322168 | .434 | 2.40826 | 4.19261 |
|  |  | AD-miWMH | HC | -7.058513 | .378124 | .227 | -8.09655 | -6.02048 |
|  |  |  | AD-nonWMH | -2.055051 | .295863 | .190 | -2.86514 | -1.24496 |
|  |  |  | AD-moWMH | 1.245380 | .334577 | .091 | .31966 | 2.17110 |
|  |  | AD-moWMH | HC | -8.303893 | .399043 | .311 | -9.40090 | -7.20689 |
|  |  |  | AD-nonWMH | -3.300431 | .322168 | .434 | -4.19261 | -2.40826 |
|  |  |  | AD-miWMH | -1.245380 | .334577 | .091 | -2.17110 | -.31966 |
| 右侧枕上回 | LSD | HC | AD-nonWMH | 1.696089 | .329101 | .114 | 1.04300 | 2.34918 |
|  |  |  | AD-miWMH | 3.744291 | .341789 | .274 | 3.06602 | 4.42256 |
|  |  |  | AD-moWMH | 4.914721 | .363661 | .150 | 4.19305 | 5.63639 |
|  |  | AD-nonWMH | HC | -1.696089 | .329101 | .114 | -2.34918 | -1.04300 |
|  |  |  | AD-miWMH | 2.048203 | .338998 | .090 | 1.37547 | 2.72093 |
|  |  |  | AD-moWMH | 3.218633 | .361039 | .077 | 2.50216 | 3.93510 |
|  |  | AD-miWMH | HC | -3.744291 | .341789 | .274 | -4.42256 | -3.06602 |
|  |  |  | AD-nonWMH | -2.048203 | .338998 | .090 | -2.72093 | -1.37547 |
|  |  |  | AD-moWMH | 1.170430 | .372641 | .082 | .43094 | 1.90992 |
|  |  | AD-moWMH | HC | -4.914721 | .363661 | .150 | -5.63639 | -4.19305 |
|  |  |  | AD-nonWMH | -3.218633 | .361039 | .077 | -3.93510 | -2.50216 |
|  |  |  | AD-miWMH | -1.170430 | .372641 | .082 | -1.90992 | -.43094 |
|  | 塔姆黑尼 | HC | AD-nonWMH | 1.696089 | .344064 | .117 | .75653 | 2.63565 |
|  |  |  | AD-miWMH | 3.744291 | .302596 | .274 | 2.91601 | 4.57257 |
|  |  |  | AD-moWMH | 4.914721 | .371342 | .150 | 3.88460 | 5.94484 |
|  |  | AD-nonWMH | HC | -1.696089 | .344064 | .117 | -2.63565 | -.75653 |
|  |  |  | AD-miWMH | 2.048203 | .330241 | .095 | 1.14399 | 2.95241 |
|  |  |  | AD-moWMH | 3.218633 | .394195 | .077 | 2.13104 | 4.30622 |
|  |  | AD-miWMH | HC | -3.744291 | .302596 | .274 | -4.57257 | -2.91601 |
|  |  |  | AD-nonWMH | -2.048203 | .330241 | .095 | -2.95241 | -1.14399 |
|  |  |  | AD-moWMH | 1.170430 | .358572 | .082 | .17059 | 2.17027 |
|  |  | AD-moWMH | HC | -4.914721 | .371342 | .150 | -5.94484 | -3.88460 |
|  |  |  | AD-nonWMH | -3.218633 | .394195 | .077 | -4.30622 | -2.13104 |
|  |  |  | AD-miWMH | -1.170430 | .358572 | .082 | -2.17027 | -.17059 |
| 左侧枕中回 | LSD | HC | AD-nonWMH | 3.556544^*^ | .254014 | .022 | 3.05246 | 4.06063 |
|  |  |  | AD-miWMH | 4.144386^*^ | .263808 | .017 | 3.62087 | 4.66790 |
|  |  |  | AD-moWMH | 5.391086^*^ | .280689 | .000 | 4.83407 | 5.94810 |
|  |  | AD-nonWMH | HC | -3.556544^*^ | .254014 | .022 | -4.06063 | -3.05246 |
|  |  |  | AD-miWMH | .587841 | .261653 | .077 | .06860 | 1.10708 |
|  |  |  | AD-moWMH | 1.834541 | .278665 | .080 | 1.28154 | 2.38754 |
|  |  | AD-miWMH | HC | -4.144386^*^ | .263808 | .017 | -4.66790 | -3.62087 |
|  |  |  | AD-nonWMH | -.587841 | .261653 | .077 | -1.10708 | -.06860 |
|  |  |  | AD-moWMH | 1.246700 | .287620 | .137 | .67593 | 1.81747 |
|  |  | AD-moWMH | HC | -5.391086^*^ | .280689 | .000 | -5.94810 | -4.83407 |
|  |  |  | AD-nonWMH | -1.834541 | .278665 | .080 | -2.38754 | -1.28154 |
|  |  |  | AD-miWMH | -1.246700 | .287620 | .137 | -1.81747 | -.67593 |
|  | 塔姆黑尼 | HC | AD-nonWMH | 3.556544^*^ | .238010 | .042 | 2.90558 | 4.20751 |
|  |  |  | AD-miWMH | 4.144386^*^ | .253548 | .033 | 3.44575 | 4.84302 |
|  |  |  | AD-moWMH | 5.391086^*^ | .267614 | .000 | 4.64347 | 6.13870 |
|  |  | AD-nonWMH | HC | -3.556544^*^ | .238010 | .042 | -4.20751 | -2.90558 |
|  |  |  | AD-miWMH | .587841 | .281125 | .225 | -.18193 | 1.35762 |
|  |  |  | AD-moWMH | 1.834541 | .293874 | .157 | 1.02254 | 2.64655 |
|  |  | AD-miWMH | HC | -4.144386^*^ | .253548 | .033 | -4.84302 | -3.44575 |
|  |  |  | AD-nonWMH | -.587841 | .281125 | .225 | -1.35762 | .18193 |
|  |  |  | AD-moWMH | 1.246700 | .306594 | .301 | .39967 | 2.09373 |
|  |  | AD-moWMH | HC | -5.391086^*^ | .267614 | .000 | -6.13870 | -4.64347 |
|  |  |  | AD-nonWMH | -1.834541 | .293874 | .157 | -2.64655 | -1.02254 |
|  |  |  | AD-miWMH | -1.246700 | .306594 | .301 | -2.09373 | -.39967 |
| 右侧枕中回 | LSD | HC | AD-nonWMH | -.034703 | .290289 | .905 | -.61077 | .54137 |
|  |  |  | AD-miWMH | .381367 | .301481 | .209 | -.21691 | .97965 |
|  |  |  | AD-moWMH | .276807 | .320773 | .390 | -.35976 | .91337 |
|  |  | AD-nonWMH | HC | .034703 | .290289 | .905 | -.54137 | .61077 |
|  |  |  | AD-miWMH | .416070 | .299019 | .167 | -.17732 | 1.00946 |
|  |  |  | AD-moWMH | .311510 | .318461 | .330 | -.32046 | .94348 |
|  |  | AD-miWMH | HC | -.381367 | .301481 | .209 | -.97965 | .21691 |
|  |  |  | AD-nonWMH | -.416070 | .299019 | .167 | -1.00946 | .17732 |
|  |  |  | AD-moWMH | -.104560 | .328695 | .751 | -.75684 | .54772 |
|  |  | AD-moWMH | HC | -.276807 | .320773 | .390 | -.91337 | .35976 |
|  |  |  | AD-nonWMH | -.311510 | .318461 | .330 | -.94348 | .32046 |
|  |  |  | AD-miWMH | .104560 | .328695 | .751 | -.54772 | .75684 |
|  | 塔姆黑尼 | HC | AD-nonWMH | -.034703 | .303139 | 1.000 | -.86204 | .79264 |
|  |  |  | AD-miWMH | .381367 | .308725 | .779 | -.46381 | 1.22654 |
|  |  |  | AD-moWMH | .276807 | .301531 | .934 | -.55286 | 1.10647 |
|  |  | AD-nonWMH | HC | .034703 | .303139 | 1.000 | -.79264 | .86204 |
|  |  |  | AD-miWMH | .416070 | .305237 | .693 | -.41916 | 1.25130 |
|  |  |  | AD-moWMH | .311510 | .297958 | .884 | -.50802 | 1.13104 |
|  |  | AD-miWMH | HC | -.381367 | .308725 | .779 | -1.22654 | .46381 |
|  |  |  | AD-nonWMH | -.416070 | .305237 | .693 | -1.25130 | .41916 |
|  |  |  | AD-moWMH | -.104560 | .303639 | 1.000 | -.94208 | .73296 |
|  |  | AD-moWMH | HC | -.276807 | .301531 | .934 | -1.10647 | .55286 |
|  |  |  | AD-nonWMH | -.311510 | .297958 | .884 | -1.13104 | .50802 |
|  |  |  | AD-miWMH | .104560 | .303639 | 1.000 | -.73296 | .94208 |
| *. 平均值差值的显著性水平为 0.05。 | | | | | | | | |

| **描述** | | | | | | | | | |
| --- | --- | --- | --- | --- | --- | --- | --- | --- | --- |
|  | | 个案数 | 平均值 | 标准 偏差 | 标准 错误 | 平均值的 95% 置信区间 | | 最小值 | 最大值 |
|  |  |  |  |  |  | 下限 | 上限 |  |  |
| 左侧海马 | HC | 28 | .96386 | .087841 | .016600 | .92980 | .99792 | .823 | 1.175 |
|  | AD-nonWMH | 29 | .66534 | .123973 | .023021 | .61819 | .71250 | .482 | .978 |
|  | AD-miWMH | 25 | .52628 | .127141 | .025428 | .47380 | .57876 | .371 | .802 |
|  | AD-moWMH | 20 | .33085 | .028658 | .006408 | .31744 | .34426 | .229 | .360 |
|  | 总计 | 102 | .64762 | .248290 | .024584 | .59885 | .69639 | .229 | 1.175 |
| 右侧海马 | HC | 28 | .94171 | .062307 | .011775 | .91755 | .96587 | .827 | 1.073 |
|  | AD-nonWMH | 29 | .74993 | .091353 | .016964 | .71518 | .78468 | .522 | .898 |
|  | AD-miWMH | 25 | .51760 | .099947 | .019989 | .47634 | .55886 | .343 | .762 |
|  | AD-moWMH | 20 | .41845 | .043489 | .009724 | .39810 | .43880 | .327 | .507 |
|  | 总计 | 102 | .68064 | .215749 | .021362 | .63826 | .72301 | .327 | 1.073 |
| 左侧颞上回 | HC | 28 | 21.84204 | 1.256881 | .237528 | 21.35467 | 22.32940 | 18.319 | 23.850 |
|  | AD-nonWMH | 29 | 18.36893 | 1.154206 | .214331 | 17.92989 | 18.80797 | 15.336 | 20.339 |
|  | AD-miWMH | 25 | 16.47688 | 2.502844 | .500569 | 15.44376 | 17.51000 | 9.884 | 19.747 |
|  | AD-moWMH | 20 | 12.69060 | 2.511944 | .561688 | 11.51497 | 13.86623 | 9.774 | 18.772 |
|  | 总计 | 102 | 17.74520 | 3.699899 | .366345 | 17.01847 | 18.47193 | 9.774 | 23.850 |
| 右侧颞上回 | HC | 28 | 21.28036 | 1.922219 | .363265 | 20.53500 | 22.02572 | 17.063 | 24.501 |
|  | AD-nonWMH | 29 | 18.12266 | 1.823965 | .338702 | 17.42886 | 18.81645 | 13.434 | 21.047 |
|  | AD-miWMH | 25 | 16.77812 | 2.473143 | .494629 | 15.75726 | 17.79898 | 10.282 | 20.228 |
|  | AD-moWMH | 20 | 10.79725 | 3.493378 | .781143 | 9.16230 | 12.43220 | 6.322 | 17.454 |
|  | 总计 | 102 | 17.22358 | 4.322111 | .427953 | 16.37464 | 18.07252 | 6.322 | 24.501 |
| 左侧颞中下回 | HC | 28 | 37.11393 | 1.912646 | .361456 | 36.37228 | 37.85558 | 33.401 | 40.283 |
|  | AD-nonWMH | 29 | 33.81597 | 2.323455 | .431455 | 32.93217 | 34.69976 | 28.719 | 38.394 |
|  | AD-miWMH | 25 | 26.68564 | 4.624628 | .924926 | 24.77669 | 28.59459 | 21.753 | 37.811 |
|  | AD-moWMH | 20 | 21.49840 | 1.721588 | .384959 | 20.69267 | 22.30413 | 17.212 | 23.801 |
|  | 总计 | 102 | 30.55845 | 6.560610 | .649597 | 29.26982 | 31.84708 | 17.212 | 40.283 |
| 右侧颞中下回 | HC | 28 | 36.17814 | 1.757102 | .332061 | 35.49681 | 36.85948 | 32.730 | 39.421 |
|  | AD-nonWMH | 29 | 31.74645 | 1.740021 | .323114 | 31.08458 | 32.40832 | 27.843 | 34.299 |
|  | AD-miWMH | 25 | 26.25736 | 2.880740 | .576148 | 25.06825 | 27.44647 | 21.883 | 31.283 |
|  | AD-moWMH | 20 | 22.41335 | 1.464116 | .327386 | 21.72812 | 23.09858 | 17.402 | 24.051 |
|  | 总计 | 102 | 29.78761 | 5.506033 | .545179 | 28.70612 | 30.86910 | 17.402 | 39.421 |
| 左侧扣带回 | HC | 28 | 1.17518 | .147041 | .027788 | 1.11816 | 1.23220 | .892 | 1.460 |
|  | AD-nonWMH | 29 | 1.13962 | .182221 | .033838 | 1.07031 | 1.20893 | .839 | 1.672 |
|  | AD-miWMH | 25 | .85656 | .056777 | .011355 | .83312 | .88000 | .734 | .953 |
|  | AD-moWMH | 20 | .77215 | .148826 | .033279 | .70250 | .84180 | .288 | .923 |
|  | 总计 | 102 | 1.00795 | .222108 | .021992 | .96432 | 1.05158 | .288 | 1.672 |
| 右侧扣带回 | HC | 28 | 1.14975 | .111796 | .021127 | 1.10640 | 1.19310 | .923 | 1.423 |
|  | AD-nonWMH | 29 | 82.21128 | 305.516136 | 56.732922 | -34.00085 | 198.42340 | .970 | 1320.000 |
|  | AD-miWMH | 25 | 1.07000 | .118696 | .023739 | 1.02100 | 1.11900 | .892 | 1.402 |
|  | AD-moWMH | 20 | .93870 | .102686 | .022961 | .89064 | .98676 | .772 | 1.207 |
|  | 总计 | 102 | 24.13573 | 165.013979 | 16.338819 | -8.27610 | 56.54755 | .772 | 1320.000 |

| **方差齐性检验** | | | | | |
| --- | --- | --- | --- | --- | --- |
|  | | 莱文统计 | 自由度 1 | 自由度 2 | 显著性 |
| 左侧海马 | 基于平均值 | 8.518 | 3 | 98 | .000 |
|  | 基于中位数 | 7.238 | 3 | 98 | .000 |
|  | 基于中位数并具有调整后自由度 | 7.238 | 3 | 77.150 | .000 |
|  | 基于剪除后平均值 | 8.557 | 3 | 98 | .000 |
| 右侧海马 | 基于平均值 | 5.128 | 3 | 98 | .002 |
|  | 基于中位数 | 4.498 | 3 | 98 | .005 |
|  | 基于中位数并具有调整后自由度 | 4.498 | 3 | 76.906 | .006 |
|  | 基于剪除后平均值 | 5.099 | 3 | 98 | .003 |
| 左侧颞上回 | 基于平均值 | 7.406 | 3 | 98 | .000 |
|  | 基于中位数 | 6.305 | 3 | 98 | .001 |
|  | 基于中位数并具有调整后自由度 | 6.305 | 3 | 68.990 | .001 |
|  | 基于剪除后平均值 | 7.686 | 3 | 98 | .000 |
| 右侧颞上回 | 基于平均值 | 5.841 | 3 | 98 | .001 |
|  | 基于中位数 | 3.387 | 3 | 98 | .021 |
|  | 基于中位数并具有调整后自由度 | 3.387 | 3 | 69.092 | .023 |
|  | 基于剪除后平均值 | 5.441 | 3 | 98 | .002 |
| 左侧颞中下回 | 基于平均值 | 7.721 | 3 | 98 | .000 |
|  | 基于中位数 | 4.451 | 3 | 98 | .006 |
|  | 基于中位数并具有调整后自由度 | 4.451 | 3 | 48.117 | .008 |
|  | 基于剪除后平均值 | 6.403 | 3 | 98 | .001 |
| 右侧颞中下回 | 基于平均值 | 10.231 | 3 | 98 | .000 |
|  | 基于中位数 | 7.823 | 3 | 98 | .000 |
|  | 基于中位数并具有调整后自由度 | 7.823 | 3 | 85.022 | .000 |
|  | 基于剪除后平均值 | 10.370 | 3 | 98 | .000 |
| 左侧扣带回 | 基于平均值 | 6.479 | 3 | 98 | .000 |
|  | 基于中位数 | 5.084 | 3 | 98 | .003 |
|  | 基于中位数并具有调整后自由度 | 5.084 | 3 | 71.963 | .003 |
|  | 基于剪除后平均值 | 5.997 | 3 | 98 | .001 |
| 右侧扣带回 | 基于平均值 | 7.900 | 3 | 98 | .000 |
|  | 基于中位数 | 1.705 | 3 | 98 | .171 |
|  | 基于中位数并具有调整后自由度 | 1.705 | 3 | 28.000 | .189 |
|  | 基于剪除后平均值 | 2.779 | 3 | 98 | .045 |

| **ANOVA** | | | | | | |
| --- | --- | --- | --- | --- | --- | --- |
|  | | 平方和 | 自由度 | 均方 | F | 显著性 |
| 左侧海马 | 组间 | 5.184 | 3 | 1.728 | 162.490 | .000 |
|  | 组内 | 1.042 | 98 | .011 |  |  |
|  | 总计 | 6.226 | 101 |  |  |  |
| 右侧海马 | 组间 | 4.087 | 3 | 1.362 | 217.388 | .000 |
|  | 组内 | .614 | 98 | .006 |  |  |
|  | 总计 | 4.701 | 101 |  |  |  |
| 左侧颞上回 | 组间 | 1032.431 | 3 | 344.144 | 96.310 | .000 |
|  | 组内 | 350.183 | 98 | 3.573 |  |  |
|  | 总计 | 1382.615 | 101 |  |  |  |
| 右侧颞上回 | 组间 | 1315.165 | 3 | 438.388 | 75.164 | .000 |
|  | 组内 | 571.579 | 98 | 5.832 |  |  |
|  | 总计 | 1886.745 | 101 |  |  |  |
| 左侧颞中下回 | 组间 | 3527.668 | 3 | 1175.889 | 140.613 | .000 |
|  | 组内 | 819.534 | 98 | 8.363 |  |  |
|  | 总计 | 4347.202 | 101 |  |  |  |
| 右侧颞中下回 | 组间 | 2653.925 | 3 | 884.642 | 212.471 | .000 |
|  | 组内 | 408.032 | 98 | 4.164 |  |  |
|  | 总计 | 3061.957 | 101 |  |  |  |
| 左侧扣带回 | 组间 | 2.971 | 3 | .990 | 48.241 | .000 |
|  | 组内 | 2.012 | 98 | .021 |  |  |
|  | 总计 | 4.983 | 101 |  |  |  |
| 右侧扣带回 | 组间 | 136666.991 | 3 | 45555.664 | 1.708 | .170 |
|  | 组内 | 2613523.944 | 98 | 26668.612 |  |  |
|  | 总计 | 2750190.936 | 101 |  |  |  |

| **多重比较** | | | | | | | | |
| --- | --- | --- | --- | --- | --- | --- | --- | --- |
| 因变量 | | (I) group | (J) group | 平均值差值 (I-J) | 标准 错误 | 显著性 | 95% 置信区间 | |
|  |  |  |  |  |  |  | 下限 | 上限 |
| 左侧海马 | LSD | HC | AD-nonWMH | .298512^*^ | .027323 | .000 | .24429 | .35273 |
|  |  |  | AD-miWMH | .437577^*^ | .028376 | .000 | .38127 | .49389 |
|  |  |  | AD-moWMH | .633007^*^ | .030192 | .000 | .57309 | .69292 |
|  |  | AD-nonWMH | HC | -.298512^*^ | .027323 | .000 | -.35273 | -.24429 |
|  |  |  | AD-miWMH | .139065^*^ | .028145 | .000 | .08321 | .19492 |
|  |  |  | AD-moWMH | .334495^*^ | .029975 | .000 | .27501 | .39398 |
|  |  | AD-miWMH | HC | -.437577^*^ | .028376 | .000 | -.49389 | -.38127 |
|  |  |  | AD-nonWMH | -.139065^*^ | .028145 | .000 | -.19492 | -.08321 |
|  |  |  | AD-moWMH | .195430^*^ | .030938 | .000 | .13403 | .25683 |
|  |  | AD-moWMH | HC | -.633007^*^ | .030192 | .000 | -.69292 | -.57309 |
|  |  |  | AD-nonWMH | -.334495^*^ | .029975 | .000 | -.39398 | -.27501 |
|  |  |  | AD-miWMH | -.195430^*^ | .030938 | .000 | -.25683 | -.13403 |
|  | 塔姆黑尼 | HC | AD-nonWMH | .298512^*^ | .028382 | .000 | .22080 | .37623 |
|  |  |  | AD-miWMH | .437577^*^ | .030367 | .000 | .35374 | .52141 |
|  |  |  | AD-moWMH | .633007^*^ | .017794 | .000 | .58336 | .68266 |
|  |  | AD-nonWMH | HC | -.298512^*^ | .028382 | .000 | -.37623 | -.22080 |
|  |  |  | AD-miWMH | .139065^*^ | .034301 | .001 | .04514 | .23299 |
|  |  |  | AD-moWMH | .334495^*^ | .023896 | .000 | .26752 | .40146 |
|  |  | AD-miWMH | HC | -.437577^*^ | .030367 | .000 | -.52141 | -.35374 |
|  |  |  | AD-nonWMH | -.139065^*^ | .034301 | .001 | -.23299 | -.04514 |
|  |  |  | AD-moWMH | .195430^*^ | .026223 | .000 | .12101 | .26985 |
|  |  | AD-moWMH | HC | -.633007^*^ | .017794 | .000 | -.68266 | -.58336 |
|  |  |  | AD-nonWMH | -.334495^*^ | .023896 | .000 | -.40146 | -.26752 |
|  |  |  | AD-miWMH | -.195430^*^ | .026223 | .000 | -.26985 | -.12101 |
| 右侧海马 | LSD | HC | AD-nonWMH | .191783^*^ | .020974 | .000 | .15016 | .23341 |
|  |  |  | AD-miWMH | .424114^*^ | .021783 | .000 | .38089 | .46734 |
|  |  |  | AD-moWMH | .523264^*^ | .023177 | .000 | .47727 | .56926 |
|  |  | AD-nonWMH | HC | -.191783^*^ | .020974 | .000 | -.23341 | -.15016 |
|  |  |  | AD-miWMH | .232331 | .021605 | .070 | .18946 | .27521 |
|  |  |  | AD-moWMH | .331481^*^ | .023010 | .000 | .28582 | .37714 |
|  |  | AD-miWMH | HC | -.424114^*^ | .021783 | .000 | -.46734 | -.38089 |
|  |  |  | AD-nonWMH | -.232331 | .021605 | .070 | -.27521 | -.18946 |
|  |  |  | AD-moWMH | .099150^*^ | .023749 | .000 | .05202 | .14628 |
|  |  | AD-moWMH | HC | -.523264^*^ | .023177 | .000 | -.56926 | -.47727 |
|  |  |  | AD-nonWMH | -.331481^*^ | .023010 | .000 | -.37714 | -.28582 |
|  |  |  | AD-miWMH | -.099150^*^ | .023749 | .000 | -.14628 | -.05202 |
|  | 塔姆黑尼 | HC | AD-nonWMH | .191783^*^ | .020650 | .000 | .13520 | .24837 |
|  |  |  | AD-miWMH | .424114^*^ | .023200 | .000 | .35985 | .48838 |
|  |  |  | AD-moWMH | .523264^*^ | .015271 | .000 | .48128 | .56525 |
|  |  | AD-nonWMH | HC | -.191783^*^ | .020650 | .000 | -.24837 | -.13520 |
|  |  |  | AD-miWMH | .232331 | .026217 | .070 | .16047 | .30420 |
|  |  |  | AD-moWMH | .331481^*^ | .019553 | .000 | .27754 | .38542 |
|  |  | AD-miWMH | HC | -.424114^*^ | .023200 | .000 | -.48838 | -.35985 |
|  |  |  | AD-nonWMH | -.232331 | .026217 | .070 | -.30420 | -.16047 |
|  |  |  | AD-moWMH | .099150^*^ | .022229 | .001 | .03709 | .16121 |
|  |  | AD-moWMH | HC | -.523264^*^ | .015271 | .000 | -.56525 | -.48128 |
|  |  |  | AD-nonWMH | -.331481^*^ | .019553 | .000 | -.38542 | -.27754 |
|  |  |  | AD-miWMH | -.099150^*^ | .022229 | .001 | -.16121 | -.03709 |
| 左侧颞上回 | LSD | HC | AD-nonWMH | 3.473105^*^ | .500834 | .000 | 2.47922 | 4.46699 |
|  |  |  | AD-miWMH | 5.365156^*^ | .520144 | .000 | 4.33295 | 6.39736 |
|  |  |  | AD-moWMH | 9.151436^*^ | .553428 | .000 | 8.05318 | 10.24970 |
|  |  | AD-nonWMH | HC | -3.473105^*^ | .500834 | .000 | -4.46699 | -2.47922 |
|  |  |  | AD-miWMH | 1.892051 | .515897 | .082 | .86827 | 2.91583 |
|  |  |  | AD-moWMH | 5.678331^*^ | .549438 | .000 | 4.58799 | 6.76867 |
|  |  | AD-miWMH | HC | -5.365156^*^ | .520144 | .000 | -6.39736 | -4.33295 |
|  |  |  | AD-nonWMH | -1.892051 | .515897 | .082 | -2.91583 | -.86827 |
|  |  |  | AD-moWMH | 3.786280^*^ | .567095 | .000 | 2.66090 | 4.91166 |
|  |  | AD-moWMH | HC | -9.151436^*^ | .553428 | .000 | -10.24970 | -8.05318 |
|  |  |  | AD-nonWMH | -5.678331^*^ | .549438 | .000 | -6.76867 | -4.58799 |
|  |  |  | AD-miWMH | -3.786280^*^ | .567095 | .000 | -4.91166 | -2.66090 |
|  | 塔姆黑尼 | HC | AD-nonWMH | 3.473105^*^ | .319933 | .000 | 2.59951 | 4.34670 |
|  |  |  | AD-miWMH | 5.365156^*^ | .554066 | .000 | 3.81892 | 6.91139 |
|  |  |  | AD-moWMH | 9.151436^*^ | .609846 | .000 | 7.41447 | 10.88840 |
|  |  | AD-nonWMH | HC | -3.473105^*^ | .319933 | .000 | -4.34670 | -2.59951 |
|  |  |  | AD-miWMH | 1.892051 | .544524 | .089 | .36736 | 3.41674 |
|  |  |  | AD-moWMH | 5.678331^*^ | .601191 | .000 | 3.95892 | 7.39775 |
|  |  | AD-miWMH | HC | -5.365156^*^ | .554066 | .000 | -6.91139 | -3.81892 |
|  |  |  | AD-nonWMH | -1.892051 | .544524 | .089 | -3.41674 | -.36736 |
|  |  |  | AD-moWMH | 3.786280^*^ | .752371 | .000 | 1.70612 | 5.86644 |
|  |  | AD-moWMH | HC | -9.151436^*^ | .609846 | .000 | -10.88840 | -7.41447 |
|  |  |  | AD-nonWMH | -5.678331^*^ | .601191 | .000 | -7.39775 | -3.95892 |
|  |  |  | AD-miWMH | -3.786280^*^ | .752371 | .000 | -5.86644 | -1.70612 |
| 右侧颞上回 | LSD | HC | AD-nonWMH | 3.157702^*^ | .639860 | .000 | 1.88792 | 4.42748 |
|  |  |  | AD-miWMH | 4.502237^*^ | .664529 | .000 | 3.18350 | 5.82097 |
|  |  |  | AD-moWMH | 10.483107^*^ | .707053 | .000 | 9.07998 | 11.88623 |
|  |  | AD-nonWMH | HC | -3.157702^*^ | .639860 | .000 | -4.42748 | -1.88792 |
|  |  |  | AD-miWMH | 1.344535^*^ | .659103 | .044 | .03657 | 2.65250 |
|  |  |  | AD-moWMH | 7.325405^*^ | .701955 | .000 | 5.93240 | 8.71841 |
|  |  | AD-miWMH | HC | -4.502237^*^ | .664529 | .000 | -5.82097 | -3.18350 |
|  |  |  | AD-nonWMH | -1.344535^*^ | .659103 | .044 | -2.65250 | -.03657 |
|  |  |  | AD-moWMH | 5.980870^*^ | .724513 | .000 | 4.54310 | 7.41864 |
|  |  | AD-moWMH | HC | -10.483107^*^ | .707053 | .000 | -11.88623 | -9.07998 |
|  |  |  | AD-nonWMH | -7.325405^*^ | .701955 | .000 | -8.71841 | -5.93240 |
|  |  |  | AD-miWMH | -5.980870^*^ | .724513 | .000 | -7.41864 | -4.54310 |
|  | 塔姆黑尼 | HC | AD-nonWMH | 3.157702^*^ | .496669 | .000 | 1.80187 | 4.51354 |
|  |  |  | AD-miWMH | 4.502237^*^ | .613693 | .000 | 2.81381 | 6.19066 |
|  |  |  | AD-moWMH | 10.483107^*^ | .861479 | .000 | 8.03967 | 12.92655 |
|  |  | AD-nonWMH | HC | -3.157702^*^ | .496669 | .000 | -4.51354 | -1.80187 |
|  |  |  | AD-miWMH | 1.344535 | .599480 | .167 | -.30757 | 2.99664 |
|  |  |  | AD-moWMH | 7.325405^*^ | .851413 | .000 | 4.90318 | 9.74763 |
|  |  | AD-miWMH | HC | -4.502237^*^ | .613693 | .000 | -6.19066 | -2.81381 |
|  |  |  | AD-nonWMH | -1.344535 | .599480 | .167 | -2.99664 | .30757 |
|  |  |  | AD-moWMH | 5.980870^*^ | .924577 | .000 | 3.39409 | 8.56765 |
|  |  | AD-moWMH | HC | -10.483107^*^ | .861479 | .000 | -12.92655 | -8.03967 |
|  |  |  | AD-nonWMH | -7.325405^*^ | .851413 | .000 | -9.74763 | -4.90318 |
|  |  |  | AD-miWMH | -5.980870^*^ | .924577 | .000 | -8.56765 | -3.39409 |
| 左侧颞中下回 | LSD | HC | AD-nonWMH | 3.297963^*^ | .766178 | .000 | 1.77751 | 4.81842 |
|  |  |  | AD-miWMH | 10.428289^*^ | .795718 | .000 | 8.84921 | 12.00737 |
|  |  |  | AD-moWMH | 15.615529^*^ | .846637 | .000 | 13.93541 | 17.29565 |
|  |  | AD-nonWMH | HC | -3.297963^*^ | .766178 | .000 | -4.81842 | -1.77751 |
|  |  |  | AD-miWMH | 7.130326 | .789221 | .122 | 5.56414 | 8.69651 |
|  |  |  | AD-moWMH | 12.317566 | .840533 | .370 | 10.64956 | 13.98558 |
|  |  | AD-miWMH | HC | -10.428289^*^ | .795718 | .000 | -12.00737 | -8.84921 |
|  |  |  | AD-nonWMH | -7.130326 | .789221 | .122 | -8.69651 | -5.56414 |
|  |  |  | AD-moWMH | 5.187240^*^ | .867544 | .000 | 3.46563 | 6.90885 |
|  |  | AD-moWMH | HC | -15.615529^*^ | .846637 | .000 | -17.29565 | -13.93541 |
|  |  |  | AD-nonWMH | -12.317566 | .840533 | .370 | -13.98558 | -10.64956 |
|  |  |  | AD-miWMH | -5.187240^*^ | .867544 | .000 | -6.90885 | -3.46563 |
|  | 塔姆黑尼 | HC | AD-nonWMH | 3.297963^*^ | .562853 | .000 | 1.76049 | 4.83544 |
|  |  |  | AD-miWMH | 10.428289^*^ | .993045 | .000 | 7.63960 | 13.21697 |
|  |  |  | AD-moWMH | 15.615529^*^ | .528057 | .000 | 14.16012 | 17.07094 |
|  |  | AD-nonWMH | HC | -3.297963^*^ | .562853 | .000 | -4.83544 | -1.76049 |
|  |  |  | AD-miWMH | 7.130326 | 1.020608 | .075 | 4.28071 | 9.97994 |
|  |  |  | AD-moWMH | 12.317566 | .578227 | .022 | 10.72907 | 13.90606 |
|  |  | AD-miWMH | HC | -10.428289^*^ | .993045 | .000 | -13.21697 | -7.63960 |
|  |  |  | AD-nonWMH | -7.130326 | 1.020608 | .075 | -9.97994 | -4.28071 |
|  |  |  | AD-moWMH | 5.187240^*^ | 1.001839 | .000 | 2.37732 | 7.99716 |
|  |  | AD-moWMH | HC | -15.615529^*^ | .528057 | .000 | -17.07094 | -14.16012 |
|  |  |  | AD-nonWMH | -12.317566 | .578227 | .022 | -13.90606 | -10.72907 |
|  |  |  | AD-miWMH | -5.187240^*^ | 1.001839 | .000 | -7.99716 | -2.37732 |
| 右侧颞中下回 | LSD | HC | AD-nonWMH | 4.431695^*^ | .540622 | .000 | 3.35885 | 5.50454 |
|  |  |  | AD-miWMH | 9.920783^*^ | .561465 | .000 | 8.80657 | 11.03499 |
|  |  |  | AD-moWMH | 13.764793^*^ | .597394 | .000 | 12.57928 | 14.95030 |
|  |  | AD-nonWMH | HC | -4.431695^*^ | .540622 | .000 | -5.50454 | -3.35885 |
|  |  |  | AD-miWMH | 5.489088 | .556880 | .180 | 4.38398 | 6.59420 |
|  |  |  | AD-moWMH | 9.333098 | .593087 | .075 | 8.15614 | 10.51006 |
|  |  | AD-miWMH | HC | -9.920783^*^ | .561465 | .000 | -11.03499 | -8.80657 |
|  |  |  | AD-nonWMH | -5.489088 | .556880 | .180 | -6.59420 | -4.38398 |
|  |  |  | AD-moWMH | 3.844010 | .612146 | .122 | 2.62923 | 5.05879 |
|  |  | AD-moWMH | HC | -13.764793^*^ | .597394 | .000 | -14.95030 | -12.57928 |
|  |  |  | AD-nonWMH | -9.333098 | .593087 | .075 | -10.51006 | -8.15614 |
|  |  |  | AD-miWMH | -3.844010 | .612146 | .122 | -5.05879 | -2.62923 |
|  | 塔姆黑尼 | HC | AD-nonWMH | 4.431695^*^ | .463322 | .000 | 3.16716 | 5.69623 |
|  |  |  | AD-miWMH | 9.920783^*^ | .664990 | .000 | 8.07739 | 11.76418 |
|  |  |  | AD-moWMH | 13.764793^*^ | .466311 | .000 | 12.48137 | 15.04821 |
|  |  | AD-nonWMH | HC | -4.431695^*^ | .463322 | .000 | -5.69623 | -3.16716 |
|  |  |  | AD-miWMH | 5.489088 | .660567 | .180 | 3.65653 | 7.32165 |
|  |  |  | AD-moWMH | 9.333098 | .459983 | .075 | 8.06738 | 10.59881 |
|  |  | AD-miWMH | HC | -9.920783^*^ | .664990 | .000 | -11.76418 | -8.07739 |
|  |  |  | AD-nonWMH | -5.489088 | .660567 | .180 | -7.32165 | -3.65653 |
|  |  |  | AD-moWMH | 3.844010^*^ | .662668 | .122 | 2.00262 | 5.68540 |
|  |  | AD-moWMH | HC | -13.764793 | .466311 | .000 | -15.04821 | -12.48137 |
|  |  |  | AD-nonWMH | -9.333098^*^ | .459983 | .075 | -10.59881 | -8.06738 |
|  |  |  | AD-miWMH | -3.844010^*^ | .662668 | .122 | -5.68540 | -2.00262 |
| 左侧扣带回 | LSD | HC | AD-nonWMH | .035558 | .037960 | .351 | -.03977 | .11089 |
|  |  |  | AD-miWMH | .318619^*^ | .039424 | .000 | .24038 | .39685 |
|  |  |  | AD-moWMH | .403029^*^ | .041946 | .000 | .31979 | .48627 |
|  |  | AD-nonWMH | HC | -.035558 | .037960 | .351 | -.11089 | .03977 |
|  |  |  | AD-miWMH | .283061^*^ | .039102 | .000 | .20546 | .36066 |
|  |  |  | AD-moWMH | .367471^*^ | .041644 | .000 | .28483 | .45011 |
|  |  | AD-miWMH | HC | -.318619^*^ | .039424 | .000 | -.39685 | -.24038 |
|  |  |  | AD-nonWMH | -.283061^*^ | .039102 | .000 | -.36066 | -.20546 |
|  |  |  | AD-moWMH | .084410 | .042982 | .052 | -.00089 | .16971 |
|  |  | AD-moWMH | HC | -.403029^*^ | .041946 | .000 | -.48627 | -.31979 |
|  |  |  | AD-nonWMH | -.367471^*^ | .041644 | .000 | -.45011 | -.28483 |
|  |  |  | AD-miWMH | -.084410 | .042982 | .052 | -.16971 | .00089 |
|  | 塔姆黑尼 | HC | AD-nonWMH | .035558 | .043785 | .962 | -.08407 | .15519 |
|  |  |  | AD-miWMH | .318619^*^ | .030019 | .000 | .23501 | .40222 |
|  |  |  | AD-moWMH | .403029^*^ | .043355 | .000 | .28316 | .52290 |
|  |  | AD-nonWMH | HC | -.035558 | .043785 | .962 | -.15519 | .08407 |
|  |  |  | AD-miWMH | .283061^*^ | .035692 | .000 | .18340 | .38272 |
|  |  |  | AD-moWMH | .367471^*^ | .047460 | .000 | .23694 | .49800 |
|  |  | AD-miWMH | HC | -.318619^*^ | .030019 | .000 | -.40222 | -.23501 |
|  |  |  | AD-nonWMH | -.283061^*^ | .035692 | .000 | -.38272 | -.18340 |
|  |  |  | AD-moWMH | .084410 | .035163 | .059 | -.01658 | .18540 |
|  |  | AD-moWMH | HC | -.403029^*^ | .043355 | .000 | -.52290 | -.28316 |
|  |  |  | AD-nonWMH | -.367471^*^ | .047460 | .000 | -.49800 | -.23694 |
|  |  |  | AD-miWMH | -.084410 | .035163 | .059 | -.18540 | .01658 |
| 右侧扣带回 | LSD | HC | AD-nonWMH | -81.061526 | 43.267282 | .064 | -166.92404 | 4.80099 |
|  |  |  | AD-miWMH | .079750 | 44.935452 | .999 | -89.09319 | 89.25269 |
|  |  |  | AD-moWMH | .211050 | 47.810888 | .996 | -94.66810 | 95.09020 |
|  |  | AD-nonWMH | HC | 81.061526 | 43.267282 | .064 | -4.80099 | 166.92404 |
|  |  |  | AD-miWMH | 81.141276 | 44.568506 | .072 | -7.30347 | 169.58603 |
|  |  |  | AD-moWMH | 81.272576 | 47.466176 | .090 | -12.92251 | 175.46766 |
|  |  | AD-miWMH | HC | -.079750 | 44.935452 | .999 | -89.25269 | 89.09319 |
|  |  |  | AD-nonWMH | -81.141276 | 44.568506 | .072 | -169.58603 | 7.30347 |
|  |  |  | AD-moWMH | .131300^*^ | 48.991581 | .048 | -97.09090 | 97.35350 |
|  |  | AD-moWMH | HC | -.211050 | 47.810888 | .996 | -95.09020 | 94.66810 |
|  |  |  | AD-nonWMH | -81.272576 | 47.466176 | .090 | -175.46766 | 12.92251 |
|  |  |  | AD-miWMH | -.131300^*^ | 48.991581 | .048 | -97.35350 | 97.09090 |
|  | 塔姆黑尼 | HC | AD-nonWMH | -81.061526 | 56.732926 | .659 | -241.62413 | 79.50108 |
|  |  |  | AD-miWMH | .079750 | .031779 | .089 | -.00734 | .16684 |
|  |  |  | AD-moWMH | .211050 | .031202 | .070 | .12501 | .29709 |
|  |  | AD-nonWMH | HC | 81.061526 | 56.732926 | .659 | -79.50108 | 241.62413 |
|  |  |  | AD-miWMH | 81.141276 | 56.732927 | .658 | -79.42133 | 241.70388 |
|  |  |  | AD-moWMH | 81.272576 | 56.732927 | .656 | -79.29003 | 241.83518 |
|  |  | AD-miWMH | HC | -.079750 | .031779 | .089 | -.16684 | .00734 |
|  |  |  | AD-nonWMH | -81.141276 | 56.732927 | .658 | -241.70388 | 79.42133 |
|  |  |  | AD-moWMH | .131300^*^ | .033027 | .002 | .04019 | .22241 |
|  |  | AD-moWMH | HC | -.211050 | .031202 | .070 | -.29709 | -.12501 |
|  |  |  | AD-nonWMH | -81.272576 | 56.732927 | .656 | -241.83518 | 79.29003 |
|  |  |  | AD-miWMH | -.131300^*^ | .033027 | .002 | -.22241 | -.04019 |
| *. 平均值差值的显著性水平为 0.05。 | | | | | | | | |

**miWMH与GMV相关性**

| **相关性** | | | | |
| --- | --- | --- | --- | --- |
|  | | | WMH | 右侧角回 |
| 斯皮尔曼 Rho | WMH | 相关系数 | 1.000 | .729^*^ |
|  |  | Sig.（双尾） | . | .026 |
|  |  | N | 25 | 25 |
|  | 右侧角回 | 相关系数 | .729^*^ | 1.000 |
|  |  | Sig.（双尾） | .026 | . |
|  |  | N | 25 | 25 |
| *. 在 0.05 级别（双尾），相关性显著。 | | |  |  |

| **相关性** | | | | |
| --- | --- | --- | --- | --- |
|  | | | WMH | 右侧额上回 |
| 斯皮尔曼 Rho | WMH | 相关系数 | 1.000 | .402^*^ |
|  |  | Sig.（双尾） | . | .031 |
|  |  | N | 25 | 25 |
|  | 右侧额上回 | 相关系数 | .402^*^ | 1.000 |
|  |  | Sig.（双尾） | .031 | . |
|  |  | N | 25 | 25 |
| *. 在 0.05 级别（双尾），相关性显著。 | | | | |

| **相关性** | | | | |
| --- | --- | --- | --- | --- |
|  | | | WMH | 右侧顶中下回 |
| 斯皮尔曼 Rho | WMH | 相关系数 | 1.000 | .533^*^ |
|  |  | Sig.（双尾） | . | .030 |
|  |  | N | 25 | 25 |
|  | 右侧顶中下回 | 相关系数 | .533^*^ | 1.000 |
|  |  | Sig.（双尾） | .030 | . |
|  |  | N | 25 | 25 |
| *. 在 0.05 级别（双尾），相关性显著。 | | |  |  |

| **相关性** | | | | |
| --- | --- | --- | --- | --- |
|  | | | WMH | 左侧额上回 |
| 斯皮尔曼 Rho | WMH | 相关系数 | 1.000 | -.429^*^ |
|  |  | Sig.（双尾） | . | .037 |
|  |  | N | 25 | 25 |
|  | 左侧额上回 | 相关系数 | -.429^*^ | 1.000 |
|  |  | Sig.（双尾） | .037 | . |
|  |  | N | 25 | 25 |
| *. 在 0.05 级别（双尾），相关性显著。 | | |  |  |

| **相关性** | | | | |
| --- | --- | --- | --- | --- |
|  | | | WMH | 左侧额中下回 |
| 斯皮尔曼 Rho | WMH | 相关系数 | 1.000 | -.302^*^ |
|  |  | Sig.（双尾） | . | .021 |
|  |  | N | 25 | 25 |
|  | 左侧额中下回 | 相关系数 | -.302^*^ | 1.000 |
|  |  | Sig.（双尾） | .021 | . |
|  |  | N | 25 | 25 |
| *. 在 0.05 级别（双尾），相关性显著。 | | | | |

| **相关性** | | | | |
| --- | --- | --- | --- | --- |
|  | | | WMH | 左侧角回 |
| 斯皮尔曼 Rho | WMH | 相关系数 | 1.000 | -.263^*^ |
|  |  | Sig.（双尾） | . | .011 |
|  |  | N | 25 | 25 |
|  | 左侧角回 | 相关系数 | -.263^*^ | 1.000 |
|  |  | Sig.（双尾） | .011 | . |
|  |  | N | 25 | 25 |
| *. 在 0.05 级别（双尾），相关性显著。 | | | | |

| **相关性** | | | | |
| --- | --- | --- | --- | --- |
|  | | | WMH | 右侧额中下回 |
| 斯皮尔曼 Rho | WMH | 相关系数 | 1.000 | -.405^*^ |
|  |  | Sig.（双尾） | . | .040 |
|  |  | N | 25 | 25 |
|  | 右侧额中下回 | 相关系数 | -.405^*^ | 1.000 |
|  |  | Sig.（双尾） | .040 | . |
|  |  | N | 25 | 25 |
| *. 在 0.05 级别（双尾），相关性显著。 | | | | |

| **相关性** | | | | |
| --- | --- | --- | --- | --- |
|  | | | WMH | 右侧顶上回 |
| 斯皮尔曼 Rho | WMH | 相关系数 | 1.000 | -.677^*^ |
|  |  | Sig.（双尾） | . | .009 |
|  |  | N | 25 | 25 |
|  | 右侧顶上回 | 相关系数 | -.677^*^ | 1.000 |
|  |  | Sig.（双尾） | .009 | . |
|  |  | N | 25 | 25 |
| *. 在 0.05 级别（双尾），相关性显著。 | | | | |

**AD-moWMH group WMH与GM皮质关系（Pearson）**

| **相关性** | | | | |
| --- | --- | --- | --- | --- |
|  | | | WMH | 左侧额上中回 |
| 斯皮尔曼 Rho | WMH | 相关系数 | 1.000 | -.311^*^ |
|  |  | Sig.（双尾） | . | .013 |
|  |  | N | 20 | 20 |
|  | 左侧额上中回 | 相关系数 | -.311^*^ | 1.000 |
|  |  | Sig.（双尾） | .013 | . |
|  |  | N | 20 | 20 |
| *. 在 0.05 级别（双尾），相关性显著。 | | |  |  |

| **相关性** | | | | |
| --- | --- | --- | --- | --- |
|  | | | WMH | 左侧额中下回 |
| 斯皮尔曼 Rho | WMH | 相关系数 | 1.000 | -.427^*^ |
|  |  | Sig.（双尾） | . | .011 |
|  |  | N | 20 | 20 |
|  | 左侧额中下回 | 相关系数 | -.427^*^ | 1.000 |
|  |  | Sig.（双尾） | .011 | . |
|  |  | N | 20 | 20 |
| *. 在 0.05 级别（双尾），相关性显著。 | | |  |  |

| **相关性** | | | | |
| --- | --- | --- | --- | --- |
|  | | | WMH | 左侧顶叶下回 |
| 斯皮尔曼 Rho | WMH | 相关系数 | 1.000 | -.308^*^ |
|  |  | Sig.（双尾） | . | .021 |
|  |  | N | 20 | 20 |
|  | 左侧顶叶下回 | 相关系数 | -.308^*^ | 1.000 |
|  |  | Sig.（双尾） | .021 | . |
|  |  | N | 20 | 20 |
| *. 在 0.05 级别（双尾），相关性显著。 | | | | |

| **相关性** | | | | |
| --- | --- | --- | --- | --- |
|  | | | WMH | 左侧角回 |
| 斯皮尔曼 Rho | WMH | 相关系数 | 1.000 | -.581^*^ |
|  |  | Sig.（双尾） | . | .033 |
|  |  | N | 20 | 20 |
|  | 左侧角回 | 相关系数 | -.581^*^ | 1.000 |
|  |  | Sig.（双尾） | .033 | . |
|  |  | N | 20 | 20 |
| *. 在 0.05 级别（双尾），相关性显著。 | | | | |

| **相关性** | | | | |
| --- | --- | --- | --- | --- |
|  | | | WMH | 左侧额前回 |
| 斯皮尔曼 Rho | WMH | 相关系数 | 1.000 | -.323^*^ |
|  |  | Sig.（双尾） | . | .019 |
|  |  | N | 20 | 20 |
|  | 左侧额前回 | 相关系数 | -.323^*^ | 1.000 |
|  |  | Sig.（双尾） | .019 | . |
|  |  | N | 20 | 20 |
| *. 在 0.05 级别（双尾），相关性显著。 | | | | |

| **相关性** | | | | |
| --- | --- | --- | --- | --- |
|  | | | WMH | 右侧额上回 |
| 斯皮尔曼 Rho | WMH | 相关系数 | 1.000 | -.372^*^ |
|  |  | Sig.（双尾） | . | .022 |
|  |  | N | 20 | 20 |
|  | 右侧额上回 | 相关系数 | -.372^*^ | 1.000 |
|  |  | Sig.（双尾） | .022 | . |
|  |  | N | 20 | 20 |
| *. 在 0.05 级别（双尾），相关性显著。 | | | | |

| **相关性** | | | | |
| --- | --- | --- | --- | --- |
|  | | | WMH | 右侧额下回 |
| 斯皮尔曼 Rho | WMH | 相关系数 | 1.000 | -.253^*^ |
|  |  | Sig.（双尾） | . | .027 |
|  |  | N | 20 | 20 |
|  | 右侧额下回 | 相关系数 | -.253^*^ | 1.000 |
|  |  | Sig.（双尾） | .027 | . |
|  |  | N | 20 | 20 |
| *. 在 0.05 级别（双尾），相关性显著。 | | | | |

| **相关性** | | | | |
| --- | --- | --- | --- | --- |
|  | | | WMH | 右侧顶上回 |
| 斯皮尔曼 Rho | WMH | 相关系数 | 1.000 | -.477^*^ |
|  |  | Sig.（双尾） | . | .010 |
|  |  | N | 20 | 20 |
|  | 右侧顶上回 | 相关系数 | -.477^*^ | 1.000 |
|  |  | Sig.（双尾） | .010 | . |
|  |  | N | 20 | 20 |
| *. 在 0.05 级别（双尾），相关性显著。 | | | | |

| **相关性** | | | | |
| --- | --- | --- | --- | --- |
|  | | | WMH | 右侧楔前回 |
| 斯皮尔曼 Rho | WMH | 相关系数 | 1.000 | -.380^*^ |
|  |  | Sig.（双尾） | . | .021 |
|  |  | N | 20 | 20 |
|  | 右侧楔前回 | 相关系数 | -.380^*^ | 1.000 |
|  |  | Sig.（双尾） | .021 | . |
|  |  | N | 20 | 20 |
| *. 在 0.05 级别（双尾），相关性显著。 | | | | |

| **相关性** | | | | |
| --- | --- | --- | --- | --- |
|  | | | WMH | 右侧角回 |
| 斯皮尔曼 Rho | WMH | 相关系数 | 1.000 | -.485^*^ |
|  |  | Sig.（双尾） | . | .017 |
|  |  | N | 20 | 20 |
|  | 右侧角回 | 相关系数 | -.485^*^ | 1.000 |
|  |  | Sig.（双尾） | .017 | . |
|  |  | N | 20 | 20 |
| *. 在 0.05 级别（双尾），相关性显著。 | | | | |

| **相关性** | | | | |
| --- | --- | --- | --- | --- |
|  | | | WMH | 右侧顶下回 |
| 斯皮尔曼 Rho | WMH | 相关系数 | 1.000 | -.262^*^ |
|  |  | Sig.（双尾） | . | .029 |
|  |  | N | 20 | 20 |
|  | 右侧顶下回 | 相关系数 | -.262^*^ | 1.000 |
|  |  | Sig.（双尾） | .029 | . |
|  |  | N | 20 | 20 |
| *. 在 0.05 级别（双尾），相关性显著。 | | | | |

| **相关性** | | | | |
| --- | --- | --- | --- | --- |
|  | | | WMH | Total GMV |
| 斯皮尔曼 Rho | WMH | 相关系数 | 1.000 | -.410^**^ |
|  |  | Sig.（双尾） | . | .005 |
|  |  | N | 45 | 45 |
|  | Total GMV | 相关系数 | -.410^**^ | 1.000 |
|  |  | Sig.（双尾） | .005 | . |
|  |  | N | 45 | 45 |
| **. 在 0.01 级别（双尾），相关性显著。 | | | | |

| **相关性** | | | | |
| --- | --- | --- | --- | --- |
|  | | | WMH | Frontal GMV |
| 斯皮尔曼 Rho | WMH | 相关系数 | 1.000 | -.654^**^ |
|  |  | Sig.（双尾） | . | .000 |
|  |  | N | 45 | 45 |
|  | Frontal GMV | 相关系数 | -.654^**^ | 1.000 |
|  |  | Sig.（双尾） | .000 | . |
|  |  | N | 45 | 45 |
| **. 在 0.01 级别（双尾），相关性显著。 | | | | |

| **相关性** | | | | |
| --- | --- | --- | --- | --- |
|  | | | WMH | Parietal GMV |
| 斯皮尔曼 Rho | WMH | 相关系数 | 1.000 | -.879^**^ |
|  |  | Sig.（双尾） | . | .000 |
|  |  | N | 45 | 45 |
|  | Parietal GMV | 相关系数 | -.879^**^ | 1.000 |
|  |  | Sig.（双尾） | .000 | . |
|  |  | N | 45 | 45 |
| **. 在 0.01 级别（双尾），相关性显著。 | | | | |

| **相关性** | | | | |
| --- | --- | --- | --- | --- |
|  | | | WMH | Temporal GMV |
| 斯皮尔曼 Rho | WMH | 相关系数 | 1.000 | -.859^**^ |
|  |  | Sig.（双尾） | . | .007 |
|  |  | N | 45 | 45 |
|  | Temporal GMV | 相关系数 | -.859^**^ | 1.000 |
|  |  | Sig.（双尾） | .007 | . |
|  |  | N | 45 | 45 |
| **. 在 0.01 级别（双尾），相关性显著。 | | | | |

| **相关性** | | | | |
| --- | --- | --- | --- | --- |
|  | | | WMH | Occipita GMV |
| 斯皮尔曼 Rho | WMH | 相关系数 | 1.000 | -.520^**^ |
|  |  | Sig.（双尾） | . | .009 |
|  |  | N | 45 | 45 |
|  | Occipita GMV | 相关系数 | -.520^**^ | 1.000 |
|  |  | Sig.（双尾） | .009 | . |
|  |  | N | 45 | 45 |
| **. 在 0.01 级别（双尾），相关性显著。 | | | | |

Run MATRIX procedure:**中介效应验证**

***************** PROCESS Procedure for SPSS Version 4.1 *****************

Written by Andrew F. Hayes, Ph.D. www.afhayes.com

Documentation available in Hayes (2018). www.guilford.com/p/hayes3

**************************************************************************

Model : 4

Y : function

X : logWMH

M : L1-（**left precuneus 左侧楔前叶**）

Covariates:

age gender

Sample

Size: 45

**************************************************************************

OUTCOME VARIABLE:

L1

Model Summary

R R-sq MSE F df1 df2 p

.9134 .8344 .0017 68.8524 3.0000 41.0000 .0220

Model

coeff se t p LLCI ULCI

constant .1001 .0803 1.2462 .0312 -.0621 .2624

logWMH -.2006 .0141 -14.2529 .0220 -.2290 -.1722

age -.0007 .0008 -.7739 .0434 -.0024 .0011

gender -.0190 .0134 -1.4194 .0233 -.0460 .0080

Standardized coefficients

coeff

logWMH -.9134

**************************************************************************

OUTCOME VARIABLE:

function

Model Summary

R R-sq MSE F df1 df2 p

.6603 .4360 .0214 7.7294 4.0000 40.0000 .0001

Model

coeff se t p LLCI ULCI

constant -2.0012 .2871 -6.9699 .0017 -2.5814 -1.4209

logWMH -.0829 .1205 -.6884 .0451 -.3264 .1605

L1 .8164 .5478 1.4903 .1440 -.2908 1.9236

age .0039 .0030 1.3175 .1952 -.0021 .0100

gender .0877 .0480 1.8265 .0753 -.0093 .1847

Test(s) of X by M interaction:

F df1 df2 p

.3156 1.0000 39.0000 .5775

Standardized coefficients

coeff

logWMH -.3311

L1 .2957

************************** TOTAL EFFECT MODEL ****************************

OUTCOME VARIABLE:

function

Model Summary

R R-sq MSE F df1 df2 p

.6361 .4046 .0220 9.2889 3.0000 41.0000 .0312

Model

coeff se t p LLCI ULCI

constant -1.9194 .2860 -6.7114 .0029 -2.4970 -1.3418

logWMH -.2467 .0501 -4.9245 .0000 -.3478 -.1455

age .0034 .0030 1.1303 .2649 -.0027 .0095

gender .0722 .0476 1.5180 .1367 -.0239 .1683

****************** CORRELATIONS BETWEEN MODEL RESIDUALS ******************

L1 function

L1 1.0000 .0000

function .0000 1.0000

************** TOTAL, DIRECT, AND INDIRECT EFFECTS OF X ON Y **************

Total effect of X on Y

Effect se t p LLCI ULCI

-.2467 .0501 -102.334 .0312 -.5478 -.3455

Direct effect of X on Y

Effect se t p LLCI ULCI

-.0829 .1205 -.6884 .0072 -.5264 .3605

Indirect effect(s) of X on Y:

Effect BootSE BootLLCI BootULCI

L1 -.1638 .1254 -.4269 -.2536

Completely standardized indirect effect(s) of X on Y:

Effect BootSE BootLLCI BootULCI

L1 -.2685 .2938 -.9030 -.2602

*********** BOOTSTRAP RESULTS FOR REGRESSION MODEL PARAMETERS ************

OUTCOME VARIABLE:

L1

Coeff BootMean BootSE BootLLCI BootULCI

constant .0214 .0213 .0296 -.0370 .0791

logWMH -.2004 -.2004 .0123 -.2240 -.1763

----------

OUTCOME VARIABLE:

function

Coeff BootMean BootSE BootLLCI BootULCI

constant -1.5594 -1.5514 .1316 -1.8060 -1.2935

logWMH -.1372 -.1283 .1329 -.3818 .1364

L1 .5551 .5827 .5875 -.5597 1.7399

*********************** ANALYSIS NOTES AND ERRORS ************************

Level of confidence for all confidence intervals in output:

95.0000

Number of bootstrap samples for percentile bootstrap confidence intervals:

5000

------ END MATRIX -----

Run MATRIX procedure:

***************** PROCESS Procedure for SPSS Version 4.1 *****************

Written by Andrew F. Hayes, Ph.D. www.afhayes.com

Documentation available in Hayes (2018). www.guilford.com/p/hayes3

**************************************************************************

Model : 4

Y : function

X : logWMH

M : L2-（**left inferior frontal trigeminal parietal 左侧额下回**）

Covariates:

age gender

Sample

Size: 45

**************************************************************************

OUTCOME VARIABLE:

L2

Model Summary

R R-sq MSE F df1 df2 p

.9184 .8434 .0005 73.6189 3.0000 41.0000 .0090

Model

coeff se t p LLCI ULCI

constant .2462 .0410 5.9993 .0130 .1633 .3291

logWMH -.1066 .0072 -14.8338 .0170 -.1211 -.0921

age -.0009 .0004 -1.9706 .0555 -.0017 .0000

gender .0792 .0068 .0049 .9961 -.0138 .0138

Standardized coefficients

coeff

logWMH -.9184

**************************************************************************

OUTCOME VARIABLE:

function

Model Summary

R R-sq MSE F df1 df2 p

.6409 .4108 .0223 6.9725 4.0000 40.0000 .0002

Model

coeff se t p LLCI ULCI

constant -1.7448 .3947 -4.4204 .0001 -2.5426 -.9471

logWMH -.3223 .1273 -2.5317 .0154 -.5796 -.0650

L2 -.7092 1.0963 -.6469 .5214 -2.9249 1.5065

age .0028 .0032 .8823 .3829 -.0036 .0092

gender .0722 .0479 1.5077 .1395 -.0246 .1691

Test(s) of X by M interaction:

F df1 df2 p

.1581 1.0000 39.0000 .6931

Standardized coefficients

coeff

logWMH -.3722

L2 .2579

************************** TOTAL EFFECT MODEL ****************************

OUTCOME VARIABLE:

function

Model Summary

R R-sq MSE F df1 df2 p

.6361 .4046 .0220 9.2889 3.0000 41.0000 .0001

Model

coeff se t p LLCI ULCI

constant -1.9194 .2860 -6.7114 .0071 -2.4970 -1.3418

logWMH -.2467 .0501 -4.9245 .0000 -.3478 -.1455

age .0034 .0030 1.1303 .2649 -.0027 .0095

gender .0722 .0476 1.5180 .1367 -.0239 .1683

****************** CORRELATIONS BETWEEN MODEL RESIDUALS ******************

L2 function

L2 1.0000 .0000

function .0000 1.0000

************** TOTAL, DIRECT, AND INDIRECT EFFECTS OF X ON Y **************

Total effect of X on Y

Effect se t p LLCI ULCI

-.2467 .0721 -11.3245 .0092 -.7478 -.3455

Direct effect of X on Y

Effect se t p LLCI ULCI

-.1111 .1273 -2.5317 .0454 -.5796 -.1650

Indirect effect(s) of X on Y:

Effect BootSE BootLLCI BootULCI

L2 -.1356 .1079 -.9027 -.3015

Completely standardized indirect effect(s) of X on Y:

Effect BootSE BootLLCI BootULCI

L2 -.2685 .2938 -.9030 - .2602

*********** BOOTSTRAP RESULTS FOR REGRESSION MODEL PARAMETERS ************

OUTCOME VARIABLE:

L2

Coeff BootMean BootSE BootLLCI BootULCI

constant .0266 .0213 .0288 -.0361 .0793

logWMH -.2037 -.2037 .0123 -.2240 -.1761

----------

OUTCOME VARIABLE:

function

Coeff BootMean BootSE BootLLCI BootULCI

constant -1.5947 -1.5514 .1316 -1.7060 -1.2935

logWMH -.1272 -.1183 .1329 -.3818 .1364

L2 .5331 .5227 .5875 -.5197 1.7399

*********************** ANALYSIS NOTES AND ERRORS ************************

Level of confidence for all confidence intervals in output:

95.0000

Number of bootstrap samples for percentile bootstrap confidence intervals:

5000

------ END MATRIX -----

Run MATRIX procedure:

***************** PROCESS Procedure for SPSS Version 4.1 *****************

Written by Andrew F. Hayes, Ph.D. www.afhayes.com

Documentation available in Hayes (2018). www.guilford.com/p/hayes3

**************************************************************************

Model : 4

Y : function

X : logWMH

M : L3-（**left angular 左侧角回**）

Covariates:

age gender

Sample

Size: 45

**************************************************************************

OUTCOME VARIABLE:

L3

Model Summary

R R-sq MSE F df1 df2 p

.9134 .8344 .0017 66.7428 3.0000 41.0000 .0000

Model

coeff se t p LLCI ULCI

constant .1001 .0803 1.2462 .0197 -.0621 .2624

logWMH -.2006 .0141 -14.2529 .0000 -.2290 -.1722

age -.0007 .0008 -.7739 .4434 -.0024 .0011

gender -.0190 .0134 -1.4194 .1633 -.0460 .0080

Standardized coefficients

coeff

logWMH -.9134

**************************************************************************

OUTCOME VARIABLE:

function

Model Summary

R R-sq MSE F df1 df2 p

.6603 .4360 .0214 7.7294 4.0000 40.0000 .0001

Model

coeff se t p LLCI ULCI

constant -1.9720 .2871 -6.8355 .0067 -2.5814 -1.4209

logWMH -.0829 .1205 -.6884 .4951 -.3264 .1605

L3 .8164 .5478 1.4903 .1440 -.2908 1.9236

age .0039 .0030 1.3175 .1952 -.0021 .0100

gender .0877 .0480 1.8265 .0753 -.0093 .1847

Test(s) of X by M interaction:

F df1 df2 p

.3156 1.0000 39.0000 .5775

Standardized coefficients

coeff

logWMH -.3750

L3 .2729

************************** TOTAL EFFECT MODEL ****************************

OUTCOME VARIABLE:

function

Model Summary

R R-sq MSE F df1 df2 p

.6361 .4046 .0220 9.2889 3.0000 41.0000 .0001

Model

coeff se t p LLCI ULCI

constant -1.9194 .2860 -6.7114 .0000 -2.4970 -1.3418

logWMH -.2467 .0501 -4.9245 .0000 -.3478 -.1455

age .0034 .0030 1.1303 .2649 -.0027 .0095

gender .0722 .0476 1.5180 .1367 -.0239 .1683

****************** CORRELATIONS BETWEEN MODEL RESIDUALS ******************

L3 function

L3 1.0000 .0000

function .0000 1.0000

************** TOTAL, DIRECT, AND INDIRECT EFFECTS OF X ON Y **************

Total effect of X on Y

Effect se t p LLCI ULCI

-.2523 .0883 -25.2840 .0130 -.8478 -.1463

Direct effect of X on Y

Effect se t p LLCI ULCI

-.0829 .2282 -.6884 .4951 -.7264 -.0605

Indirect effect(s) of X on Y:

Effect BootSE BootLLCI BootULCI

L3 -.1794 .1249 -.6295 -.1580

Completely standardized indirect effect(s) of X on Y:

Effect BootSE BootLLCI BootULCI

L3 -.2866 .2372 -.7230 -.3102

*********** BOOTSTRAP RESULTS FOR REGRESSION MODEL PARAMETERS ************

OUTCOME VARIABLE:

L3

Coeff BootMean BootSE BootLLCI BootULCI

constant .3920 .0277 .0213 -.0422 .0829

logWMH -.2533 -.2088 .0773 -.2709 -.2066

----------

OUTCOME VARIABLE:

function

Coeff BootMean BootSE BootLLCI BootULCI

constant -2.0571 -1.5712 .1604 -1.5560 -1.2745

logWMH -.1429 -.1087 .1229 -.3771 .1630

L3 .6531 .5271 .6805 -.6147 1.5379

*********************** ANALYSIS NOTES AND ERRORS ************************

Level of confidence for all confidence intervals in output:

95.0000

Number of bootstrap samples for percentile bootstrap confidence intervals:

5000

------ END MATRIX -----

Run MATRIX procedure:

***************** PROCESS Procedure for SPSS Version 4.1 *****************

Written by Andrew F. Hayes, Ph.D. www.afhayes.com

Documentation available in Hayes (2018). www.guilford.com/p/hayes3

**************************************************************************

Model : 4

Y : function

X : logWMH

M : R1-（right supramarginal 右侧缘上回）

Covariates:

age gender

Sample

Size: 45

**************************************************************************

OUTCOME VARIABLE:

R1

Model Summary

R R-sq MSE F df1 df2 p

.9684 .9378 .0002 205.9005 3.0000 41.0000 .0000

Model

coeff se t p LLCI ULCI

constant .1123 .0248 4.5235 .0001 .0622 .1625

logWMH -.1075 .0044 -24.7164 .0075 -.1163 -.0987

age .0002 .0003 .7425 .4620 -.0003 .0007

gender .0023 .0041 .5688 .5726 -.0060 .0107

Standardized coefficients

coeff

logWMH -.9684

**************************************************************************

OUTCOME VARIABLE:

function

Model Summary

R R-sq MSE F df1 df2 p

.6383 .4074 .0225 6.8747 4.0000 40.0000 .0003

Model

coeff se t p LLCI ULCI

constant -1.8315 .3537 -5.1782 .0000 -2.5463 -1.1167

logWMH -.3308 .2018 -1.6397 .1089 -.7386 .0770

R1 -.7825 1.8165 -.4308 .6690 -4.4539 2.8889

age .0036 .0031 1.1611 .2525 -.0026 .0097

gender .0741 .0482 1.5351 .1326 -.0234 .1715

Test(s) of X by M interaction:

F df1 df2 p

.2113 1.0000 39.0000 .6483

Standardized coefficients

coeff

logWMH -.4130

R1 .2926

************************** TOTAL EFFECT MODEL ****************************

OUTCOME VARIABLE:

function

Model Summary

R R-sq MSE F df1 df2 p

.6361 .4046 .0220 9.2889 3.0000 41.0000 .0001

Model

coeff se t p LLCI ULCI

constant -1.9194 .2860 -6.7114 .0000 -2.4970 -1.3418

logWMH -.2467 .0501 -4.9245 .0000 -.3478 -.1455

age .0034 .0030 1.1303 .2649 -.0027 .0095

gender .0722 .0476 1.5180 .1367 -.0239 .1683

****************** CORRELATIONS BETWEEN MODEL RESIDUALS ******************

R1 function

R1 1.0000 .0000

function .0000 1.0000

************** TOTAL, DIRECT, AND INDIRECT EFFECTS OF X ON Y **************

Total effect of X on Y

Effect se t p LLCI ULCI

-.3049 .1307 -21.733 .0038 -.9478 -.4462

Direct effect of X on Y

Effect se t p LLCI ULCI

-.1208 .2018 -1.6397 .1089 -.7386 -.3770

Indirect effect(s) of X on Y:

Effect BootSE BootLLCI BootULCI

R1 -.1841 .2339 -0.6948 -.4520

Completely standardized indirect effect(s) of X on Y:

Effect BootSE BootLLCI BootULCI

R1 -.1866 .1680 -0.6437 -.2209

*********** BOOTSTRAP RESULTS FOR REGRESSION MODEL PARAMETERS ************

OUTCOME VARIABLE:

R1

Coeff BootMean BootSE BootLLCI BootULCI

constant .3973 .0295 .0312 -.0382 .0799

logWMH -.2883 -.2438 .0773 -.2477 -.1836

----------

OUTCOME VARIABLE:

function

Coeff BootMean BootSE BootLLCI BootULCI

constant -1.8093 -2.0733 .2047 -1.9860 -1.7645

logWMH -.1472 -.1387 .1229 -.3971 .1842

R1 .5774 .7791 .6305 -.5947 2.0379

*********************** ANALYSIS NOTES AND ERRORS ************************

Level of confidence for all confidence intervals in output:

95.0000

Number of bootstrap samples for percentile bootstrap confidence intervals:

5000

------ END MATRIX -----

Run MATRIX procedure:

***************** PROCESS Procedure for SPSS Version 4.1 *****************

Written by Andrew F. Hayes, Ph.D. www.afhayes.com

Documentation available in Hayes (2018). www.guilford.com/p/hayes3

**************************************************************************

Model : 4

Y : function

X : logWMH

M : R2-（**right superior parietal 右侧顶上回**）

Covariates:

age gender

Sample

Size: 45

**************************************************************************

OUTCOME VARIABLE:

R2

Model Summary

R R-sq MSE F df1 df2 p

.9725 .9457 .0001 238.1829 3.0000 41.0000 .0000

Model

coeff se t p LLCI ULCI

constant .1535 .0235 6.5368 .0190 .1060 .2009

logWMH -.1095 .0041 -26.6363 .0277 -.1178 -.1012

age .0001 .0002 .2765 .7836 -.0004 .0006

gender .0034 .0039 .8768 .3857 -.0045 .0113

Standardized coefficients

coeff

logWMH -.9725

**************************************************************************

OUTCOME VARIABLE:

function

Model Summary

R R-sq MSE F df1 df2 p

.6658 .4432 .0211 7.9613 4.0000 40.0000 .0001

Model

coeff se t p LLCI ULCI

constant -1.4434 .4001 -3.6072 .0008 -2.2521 -.6347

logWMH -.5864 .2098 -2.7948 .0079 -1.0105 -.1623

R2 -3.1019 1.8627 -1.6653 .1037 -6.8666 .6628

age .0036 .0030 1.2252 .2277 -.0023 .0096

gender .0828 .0470 1.7621 .0857 -.0122 .1778

Test(s) of X by M interaction:

F df1 df2 p

.7492 1.0000 39.0000 .3920

Standardized coefficients

coeff

logWMH -.3831

R2 .3057

************************** TOTAL EFFECT MODEL ****************************

OUTCOME VARIABLE:

function

Model Summary

R R-sq MSE F df1 df2 p

.6361 .4046 .0220 9.2889 3.0000 41.0000 .0001

Model

coeff se t p LLCI ULCI

constant -1.9194 .2860 -6.7114 .0000 -2.4970 -1.3418

logWMH -.2467 .0501 -4.9245 .0000 -.3478 -.1455

age .0034 .0030 1.1303 .2649 -.0027 .0095

gender .0722 .0476 1.5180 .1367 -.0239 .1683

****************** CORRELATIONS BETWEEN MODEL RESIDUALS ******************

R2 function

R2 1.0000 .0000

function .0000 1.0000

************** TOTAL, DIRECT, AND INDIRECT EFFECTS OF X ON Y **************

Total effect of X on Y

Effect se t p LLCI ULCI

-.6261 .0433 -18.695 .0110 -.3478 -.1455

Direct effect of X on Y

Effect se t p LLCI ULCI

-.2864 .2098 -2.7948 .0079 -1.0105 -.1623

Indirect effect(s) of X on Y:

Effect BootSE BootLLCI BootULCI

R2 -.3397 .2037 -.8807 -.2295

Completely standardized indirect effect(s) of X on Y:

Effect BootSE BootLLCI BootULCI

R2 -.2548 .2788 -.8330 .2902

*********** BOOTSTRAP RESULTS FOR REGRESSION MODEL PARAMETERS ************

OUTCOME VARIABLE:

R2

Coeff BootMean BootSE BootLLCI BootULCI

constant .0270 .0252 .0325 -.0391 .0877

logWMH -.1884 -.1924 .0423 -.2840 -.1663

----------

OUTCOME VARIABLE:

function

Coeff BootMean BootSE BootLLCI BootULCI

constant -1.9402 -1.5514 .1316 -1.8060 -1.2935

logWMH -.1229 -.1283 .1270 -.3820 .1426

R2 .6053 .5277 .5450 -.5137 1.5479

*********************** ANALYSIS NOTES AND ERRORS ************************

Level of confidence for all confidence intervals in output:

95.0000

Number of bootstrap samples for percentile bootstrap confidence intervals:

5000

------ END MATRIX -----

Run MATRIX procedure:

***************** PROCESS Procedure for SPSS Version 4.1 *****************

Written by Andrew F. Hayes, Ph.D. www.afhayes.com

Documentation available in Hayes (2018). www.guilford.com/p/hayes3

**************************************************************************

Model : 4

Y : memory

X : logWMH

M : L1-（**left middle frontal 左侧额中回**）

Covariates:

age gender

Sample

Size: 45

**************************************************************************

OUTCOME VARIABLE:

L1

Model Summary

R R-sq MSE F df1 df2 p

.4275 .5044 .0017 62.8820 3.0000 41.0000 .0000

Model

coeff se t p LLCI ULCI

constant .1001 .0803 1.2462 .0219 -.0621 .2624

logWMH -.2096 .0141 -18.2570 .0160 -.2290 -.1722

age -.0420 .0008 -.7289 .4439 -.0024 .0019

gender -.0480 .5562 1.4237 .3488 -.5270 1.6109

Standardized coefficients

coeff

logWMH -.4275

**************************************************************************

OUTCOME VARIABLE:

memory

Model Summary

R R-sq MSE F df1 df2 p

.5577 .3110 .0687 4.5142 4.0000 40.0000 .0042

Model

coeff se t p LLCI ULCI

constant -2.2449 .5146 -4.3621 .0001 -3.2850 -1.2048

logWMH -.2372 .2159 -1.0986 .0275 -.6736 .1992

L1 .6086 .9819 .6199 .5389 -1.3759 2.5931

age .0372 .0420 .6553 .5749 -.0502 .0877

gender .0669 .0861 .7769 .4418 -.1071 .2408

Test(s) of X by M interaction:

F df1 df2 p

3.1942 1.0000 47.0000 .0817

Standardized coefficients

coeff

logWMH -.4217

L1 .3073

************************** TOTAL EFFECT MODEL ****************************

OUTCOME VARIABLE:

memory

Model Summary

R R-sq MSE F df1 df2 p

.5517 .3044 .0676 5.9806 3.0000 41.0000 .0018

Model

coeff se t p LLCI ULCI

constant -2.1839 .5013 -4.3562 .0001 -3.1964 -1.1714

logWMH -.3593 .0878 -4.0915 .0002 -.5366 -.1819

age .0038 .0053 .7142 .5532 -.0069 .0144

gender .0553 .0834 .5633 .6408 -.1131 .2237

****************** CORRELATIONS BETWEEN MODEL RESIDUALS ******************

L1 memory

L1 1.0000 .0000

memory .0000 1.0000

************** TOTAL, DIRECT, AND INDIRECT EFFECTS OF X ON Y **************

Total effect of X on Y

Effect se t p LLCI ULCI

-.5199 .0878 -23.064 .0442 -.5366 -.1819

Direct effect of X on Y

Effect se t p LLCI ULCI

-.2372 .2159 -1.0986 .2785 -.6736 -.1992

Indirect effect(s) of X on Y:

Effect BootSE BootLLCI BootULCI

L1 -.2827 .1936 -.8490 -.2007

Completely standardized indirect effect(s) of X on Y:

Effect BootSE BootLLCI BootULCI

L1 -.2866 .3160 -.8843 -.2827

*********** BOOTSTRAP RESULTS FOR REGRESSION MODEL PARAMETERS ************

OUTCOME VARIABLE:

L1

Coeff BootMean BootSE BootLLCI BootULCI

constant .0372 .0273 .0336 -.0382 .0707

logWMH -.2433 -.2209 .0172 -.2390 -.1563

----------

OUTCOME VARIABLE:

memory

Coeff BootMean BootSE BootLLCI BootULCI

constant -1.3792 -1.7014 .1516 -1.8060 -1.2935

logWMH -.1533 -.1283 .1479 -.3818 .1364

L1 .6021 .5827 .5875 -.5622 1.7399

*********************** ANALYSIS NOTES AND ERRORS ************************

Level of confidence for all confidence intervals in output:

95.0000

Number of bootstrap samples for percentile bootstrap confidence intervals:

5000

------ END MATRIX -----

Run MATRIX procedure:

***************** PROCESS Procedure for SPSS Version 4.1 *****************

Written by Andrew F. Hayes, Ph.D. www.afhayes.com

Documentation available in Hayes (2018). www.guilford.com/p/hayes3

**************************************************************************

Model : 4

Y : memory

X : logWMH

M : L2（**left inferior frontal trigeminal parietal 左侧额下三角**）

Covariates:

age gender

Sample

Size: 45

**************************************************************************

OUTCOME VARIABLE:

L2

Model Summary

R R-sq MSE F df1 df2 p

.9530 .7827 .0032 43.7246 1.0000 37.7301 .0290

Model

coeff se t p LLCI ULCI

constant .0317 .0346 .4203 .0377 -.0592 .0822

logWMH -.2059 .0166 -12.3620 .0187 -.2055 -.2326

age -.0709 .0004 -9.9703 .0522 -.0017 .0000

gender .0107 .0068 .0049 .7833 -.0138 .0138

Standardized coefficients

coeff

logWMH -.9530

**************************************************************************

OUTCOME VARIABLE:

memory

Model Summary

R R-sq MSE F df1 df2 p

.6007 .3608 .0637 5.6453 4.0000 40.0000 .0011

Model

coeff se t p LLCI ULCI

constant -1.3273 .6667 -1.9907 .0534 -2.6748 .0203

logWMH -.7303 .2150 -3.3962 .0016 -1.1649 -.2957

L2 -3.4799 1.8518 -1.8793 .0675 -7.2225 .2627

age .0008 .0054 .1506 .8811 -.0100 .0116

gender .5226 .5476 1.0327 .3066 -.5120 1.6701

Test(s) of X by M interaction:

F df1 df2 p

.2987 1.0000 39.0000 .5878

Standardized coefficients

coeff

logWMH -.3582

L2 .2669

************************** TOTAL EFFECT MODEL ****************************

OUTCOME VARIABLE:

memory

Model Summary

R R-sq MSE F df1 df2 p

.5517 .3044 .0676 5.9806 3.0000 41.0000 .0018

Model

coeff se t p LLCI ULCI

constant -2.1839 .5013 -7.4019 .0017 -3.1964 -1.1714

logWMH -.3593 .0878 -4.0915 .0002 -.5366 -.1819

age .0038 .0053 .7142 .4792 -.0069 .0144

gender .0553 .0834 .6633 .5108 -.1131 .2237

****************** CORRELATIONS BETWEEN MODEL RESIDUALS ******************

L2 memory

L2 1.0000 .0000

memory .0000 1.0000

************** TOTAL, DIRECT, AND INDIRECT EFFECTS OF X ON Y **************

Total effect of X on Y

Effect se t p LLCI ULCI

-1.3017 .0570 -11.746 .0372 -.5366 -.1819

Direct effect of X on Y

Effect se t p LLCI ULCI

-.5303 .2150 -3.3962 .0116 -1.1649 -.2957

Indirect effect(s) of X on Y:

Effect BootSE BootLLCI BootULCI

L2 -.7714 .1938 -.9259 -.3665

Completely standardized indirect effect(s) of X on Y:

Effect BootSE BootLLCI BootULCI

L2 -.7925 .3011 -.8840 .2755

*********** BOOTSTRAP RESULTS FOR REGRESSION MODEL PARAMETERS ************

OUTCOME VARIABLE:

L2

Coeff BootMean BootSE BootLLCI BootULCI

constant .0317 .0259 .0327 -.0311 .0883

logWMH -.2059 -.2271 .0131 -.2470 -.1761

----------

OUTCOME VARIABLE:

memory

Coeff BootMean BootSE BootLLCI BootULCI

constant -1.9547 -1.5514 .1560 -1.4360 -.9435

logWMH -.1472 -.1083 .1490 -.3580 .1364

L2 .5226 .4773 .5025 -.5397 1.3370

*********************** ANALYSIS NOTES AND ERRORS ************************

Level of confidence for all confidence intervals in output:

95.0000

Number of bootstrap samples for percentile bootstrap confidence intervals:

5000

------ END MATRIX -----

Run MATRIX procedure:

***************** PROCESS Procedure for SPSS Version 4.1 *****************

Written by Andrew F. Hayes, Ph.D. www.afhayes.com

Documentation available in Hayes (2018). www.guilford.com/p/hayes3

**************************************************************************

Model : 4

Y : memory

X : logWMH

M : L3-（**left PPC 左侧后顶叶皮质**）

Covariates:

age gender

Sample

Size: 45

**************************************************************************

OUTCOME VARIABLE:

L3

Model Summary

R R-sq MSE F df1 df2 p

.7740 .5079 .0033 103.5446 1.0000 40.0000 .0370

Model

coeff se t p LLCI ULCI

constant .4020 .0350 .5033 .0029 -.0502 .0783

logWMH -.2632 .0131 -15.2270 .01474 -.2174 -.2366

age -.0827 .0108 -.7029 .3982 -.0027 .0011

gender -.0194 .0177 -1.4109 .1496 -.0250 .0080

Standardized coefficients

coeff

logWMH -.7740

**************************************************************************

OUTCOME VARIABLE:

memory

Model Summary

R R-sq MSE F df1 df2 p

.7992 .4038 .0372 19.5003 3.0000 35.0270 .0033

Model

coeff se t p LLCI ULCI

constant -1.9547 .1262 -12.3589 .0001 -3.2850 -1.2048

logWMH -.2372 .2159 -1.0986 .2785 -.6736 .1992

L3 .6086 .9819 .6199 .5389 -1.3759 2.5931

age .0042 .0054 .7780 .4411 -.0067 .0150

gender .0669 .0861 .7769 .4418 -.1071 .2408

Test(s) of X by M interaction:

F df1 df2 p

3.1942 1.0000 39.0000 .0817

Standardized coefficients

coeff

logWMH -.4425

L3 .3023

************************** TOTAL EFFECT MODEL ****************************

OUTCOME VARIABLE:

memory

Model Summary

R R-sq MSE F df1 df2 p

.5517 .3044 .0676 5.9806 3.0000 41.0000 .0018

Model

coeff se t p LLCI ULCI

constant -2.1839 .5013 -4.3562 .0001 -3.1964 -1.1714

logWMH -.3593 .0878 -4.0915 .0002 -.5366 -.1819

age .0038 .0053 .7142 .4792 -.0069 .0144

gender .0553 .0834 .6633 .5108 -.1131 .2237

****************** CORRELATIONS BETWEEN MODEL RESIDUALS ******************

L3 memory

L3 1.0000 .0000

memory .0000 1.0000

************** TOTAL, DIRECT, AND INDIRECT EFFECTS OF X ON Y **************

Total effect of X on Y

Effect se t p LLCI ULCI

-.5493 .0878 -35.262 .0312 -.7366 -.2819

Direct effect of X on Y

Effect se t p LLCI ULCI

-.2372 .1159 -1.0986 .2785 -.6736 -.1822

Indirect effect(s) of X on Y:

Effect BootSE BootLLCI BootULCI

L3 -.3121 .1842 -.5518 -.2131

Completely standardized indirect effect(s) of X on Y:

Effect BootSE BootLLCI BootULCI

L3 -.3142 .2740 -.6693 -.1241

*********** BOOTSTRAP RESULTS FOR REGRESSION MODEL PARAMETERS ************

OUTCOME VARIABLE:

L3

Coeff BootMean BootSE BootLLCI BootULCI

constant .4020 .0328 .0397 -.0483 .0919

logWMH -.2632 -.2570 .1223 -.3349 -.2073

----------

OUTCOME VARIABLE:

memory

Coeff BootMean BootSE BootLLCI BootULCI

constant -1.9032 -1.4470 .2204 -1.7630 -1.2219

logWMH -.1663 -.1330 .1079 -.4072 .1830

L3 .6237 .5217 .6505 -.5770 1.4382

*********************** ANALYSIS NOTES AND ERRORS ************************

Level of confidence for all confidence intervals in output:

95.0000

Number of bootstrap samples for percentile bootstrap confidence intervals:

5000

------ END MATRIX -----

Run MATRIX procedure:

***************** PROCESS Procedure for SPSS Version 4.1 *****************

Written by Andrew F. Hayes, Ph.D. www.afhayes.com

Documentation available in Hayes (2018). www.guilford.com/p/hayes3

**************************************************************************

Model : 4

Y : memory

X : logWMH

M : R1-（**right supramarginal 右侧缘上回**）

Covariates:

age gender

Sample

Size: 45

**************************************************************************

OUTCOME VARIABLE:

R1

Model Summary

R R-sq MSE F df1 df2 p

.7335 .6570 .0022 73.5582 1.0000 33.2900 .0320

Model

coeff se t p LLCI ULCI

constant .3029 .0390 .6073 .0277 -.0492 .0833

logWMH -.2753 .0324 -22.7046 .0133 -.1955 -.3517

age .0802 .1332 .5822 .4220 -.0003 .0007

gender .0093 .0098 .8280 .4476 -.0060 .0107

Standardized coefficients

coeff

logWMH -.7335

**************************************************************************

OUTCOME VARIABLE:

memory

Model Summary

R R-sq MSE F df1 df2 p

.4530 .3058 .0692 4.4056 4.0000 40.0000 .0048

Model

coeff se t p LLCI ULCI

constant -1.9032 .2073 -14.4238 .0012 -2.0190 -1.3208

logWMH -.4576 .3541 -1.2923 .2037 -1.1734 .2581

R1 -.1663 .1581 -1.2275 .3179 -.4223 .1720

age .0277 .0187 .7345 .4269 -.0069 .0148

gender .0595 .0847 .6787 .5013 -.1137 .2286

Test(s) of X by M interaction:

F df1 df2 p

2.2777 1.0000 39.0000 .1393

Standardized coefficients

coeff

logWMH -.4730

R1 .3011

************************** TOTAL EFFECT MODEL ****************************

OUTCOME VARIABLE:

memory

Model Summary

R R-sq MSE F df1 df2 p

.5517 .3044 .0676 5.9806 3.0000 41.0000 .0018

Model

coeff se t p LLCI ULCI

constant -2.1839 .5013 -4.3562 .0001 -3.1964 -1.1714

logWMH -.3593 .0878 -4.0915 .0002 -.5366 -.1819

age .0038 .0053 .7142 .4792 -.0069 .0144

gender .0553 .0834 .6633 .5108 -.1131 .2237

****************** CORRELATIONS BETWEEN MODEL RESIDUALS ******************

R1 memory

R1 1.0000 .0000

memory .0000 1.0000

************** TOTAL, DIRECT, AND INDIRECT EFFECTS OF X ON Y **************

Total effect of X on Y

Effect se t p LLCI ULCI

-.4577 .1308 -26.511 .0220 -.5366 -.1819

Direct effect of X on Y

Effect se t p LLCI ULCI

-.0984 .3541 -1.2923 .2037 -.7734 -.2581

Indirect effect(s) of X on Y:

Effect BootSE BootLLCI BootULCI

R1 -.3593 .3690 -.5551 -.2486

Completely standardized indirect effect(s) of X on Y:

Effect BootSE BootLLCI BootULCI

R1 -.2750 .1680 -.4017 -.2933

*********** BOOTSTRAP RESULTS FOR REGRESSION MODEL PARAMETERS ************

OUTCOME VARIABLE:

R1

Coeff BootMean BootSE BootLLCI BootULCI

constant .3029 .0315 .0302 -.0442 .0739

logWMH -.2753 -.2385 .0527 -.2787 -.1727

----------

OUTCOME VARIABLE:

memory

Coeff BootMean BootSE BootLLCI BootULCI

constant -2.4063 -2.4133 .2552 -1.9072 -1.7705

logWMH -.1726 -.2282 .1595 -.3259 .2072

R1 .6631 .7529 .6118 -.5807 1.8873

*********************** ANALYSIS NOTES AND ERRORS ************************

Level of confidence for all confidence intervals in output:

95.0000

Number of bootstrap samples for percentile bootstrap confidence intervals:

5000

------ END MATRIX -----

Run MATRIX procedure:

***************** PROCESS Procedure for SPSS Version 4.1 *****************

Written by Andrew F. Hayes, Ph.D. www.afhayes.com

Documentation available in Hayes (2018). www.guilford.com/p/hayes3

**************************************************************************

Model : 4

Y : memory

X : logWMH

M : R2 -（**right PPC 右侧后顶叶皮质**）

Covariates:

age gender

Sample

Size: 45

**************************************************************************

OUTCOME VARIABLE:

R2

Model Summary

R R-sq MSE F df1 df2 p

.7789 .8328 .0033 17.9233 1.0000 40.0770 .0390

Model

coeff se t p LLCI ULCI

constant .0533 .0702 .5721 .0384 -.0812 .0787

logWMH -.1752 .0481 -11.2076 .0330 -.2770 -.1729

age .0331 .0072 .2067 .7046 -.0034 .0706

gender .0042 .0077 .8018 .6237 -.0145 .0113

Standardized coefficients

coeff

logWMH -.7789

**************************************************************************

OUTCOME VARIABLE:

memory

Model Summary

R R-sq MSE F df1 df2 p

.6277 .5023 .0576 20.7738 4.0000 39.2820 .0027

Model

coeff se t p LLCI ULCI

constant -1.6870 .1890 -18.4290 .0029 -1.4427 -1.3424

logWMH -.1729 .1208 -1.1351 .0528 -.3810 .2257

R2 -2.4078 3.3551 -.7176 .4771 -9.1887 4.3732

age .0039 .0053 .7402 .4635 -.0068 .0147

gender .0636 .0847 .7507 .4573 -.1076 .2347

Test(s) of X by M interaction:

F df1 df2 p

4.2069 1.0000 39.0000 .0470

Standardized coefficients

coeff

logWMH -.4427

R2 .3219

************************** TOTAL EFFECT MODEL ****************************

OUTCOME VARIABLE:

memory

Model Summary

R R-sq MSE F df1 df2 p

.6277 .5023 .0576 20.7738 4.0000 39.2820 .0027

Model

coeff se t p LLCI ULCI

constant -2.1839 .5013 -4.3562 .0001 -3.1964 -1.1714

logWMH -.3593 .0878 -4.0915 .0002 -.5366 -.1819

age .0038 .0053 .7142 .4792 -.0069 .0144

gender .0553 .0834 .6633 .5108 -.1131 .2237

****************** CORRELATIONS BETWEEN MODEL RESIDUALS ******************

R2 memory

R2 1.0000 .0000

memory .0000 1.0000

************** TOTAL, DIRECT, AND INDIRECT EFFECTS OF X ON Y **************

Total effect of X on Y

Effect se t p LLCI ULCI

-.8867 .0920 -42.397 .0352 -.7366 -.2007

Direct effect of X on Y

Effect se t p LLCI ULCI

-.3630 .2779 -1.6484 .1071 -1.3868 -.1409

Indirect effect(s) of X on Y:

Effect BootSE BootLLCI BootULCI

R2 -.5237 .3537 -.9651 -.2543

Completely standardized indirect effect(s) of X on Y:

Effect BootSE BootLLCI BootULCI

R2 -.4380 .2570 -.7729 -.3170

*********** BOOTSTRAP RESULTS FOR REGRESSION MODEL PARAMETERS ************

OUTCOME VARIABLE:

R2

Coeff BootMean BootSE BootLLCI BootULCI

constant .0533 .0392 .0477 -.0521 .0729

logWMH -.1752 -.2027 .0822 -.2180 -.1763

----------

OUTCOME VARIABLE:

memory

Coeff BootMean BootSE BootLLCI BootULCI

constant -1.6870 -1.5240 .1722 -1.5172 -1.4320

logWMH -.1729 -.1433 .1270 -.2973 .1977

R2 .6233 .5027 .4473 -.5219 1.2488

*********************** ANALYSIS NOTES AND ERRORS ************************

Level of confidence for all confidence intervals in output:

95.0000

Number of bootstrap samples for percentile bootstrap confidence intervals:

5000

------ END MATRIX -----

*****************************************************************************************************************************************************8
